# BRD4 promotes metastatic potential in oral squamous cell carcinoma through the epigenetic regulation of the *MMP2* gene

**DOI:** 10.1038/s41416-020-0907-6

**Published:** 2020-06-05

**Authors:** Tatsuro Yamamoto, Akiyuki Hirosue, Masafumi Nakamoto, Ryoji Yoshida, Junki Sakata, Yuichiro Matsuoka, Kenta Kawahara, Yuka Nagao, Masashi Nagata, Nozomu Takahashi, Akimitsu Hiraki, Masanori Shinohara, Mitsuyoshi Nakao, Noriko Saitoh, Hideki Nakayama

**Affiliations:** 1 Department of Oral and Maxillofacial Surgery, Faculty of Life Sciences, Kumamoto University, Kumamoto, 860-8556 Japan; 2 Division of Cancer Biology, The Cancer Institute of JFCR, Tokyo, 135-8550 Japan; 3 Department of Medical Cell Biology, Institute of Molecular Embryology and Genetics, Kumamoto University, Kumamoto, 860-0811 Japan; 4 Section of Oral Oncology, Department of Oral and Maxillofacial Surgery, Fukuoka Dental College, Fukuoka, 814-0193 Japan

**Keywords:** Chromatin remodelling, Cell invasion, Oral cancer detection

## Abstract

**Background:**

Oral squamous cell carcinoma (OSCC) has increased morbidity, and its high metastatic potential affects patient survival. Bromodomain containing 4 (BRD4) is a chromatin protein that associates with acetylated histone lysines and facilitates transcription. BRD4 has been implicated in cell proliferation, metastasis, and prognosis in several types of cancer. However, the role of BRD4 in OSCC remains to be elucidated.

**Methods:**

We investigated the role of BRD4 and its potential utility as a therapeutic target in OSCC.

**Results:**

JQ1, the BRD4 inhibitor, suppressed the cell proliferation, migration, and invasion in the OSCC cell lines and in vivo. JQ1 reduced the expression levels of 15 metastasis genes in OSCC, including *matrix metallopeptidase 2* (*MMP2*). Our chromatin immunoprecipitation assay showed that JQ1 reduced the BRD4 binding to the histone H3 lysine 27 acetylation-enriched sites in the *MMP2* locus. Analyses of biopsy specimens from OSCC patients revealed that the *BRD4* and *MMP2* expression levels were correlated in the cancerous regions, and both were highly expressed in lymph node metastasis cases, including delayed metastasis.

**Conclusions:**

BRD4 contributes to metastasis in OSCC, through the epigenetic regulation of the *MMP2* gene, and thus BRD4 may represent a therapeutic target and a novel prediction indicator for metastasis.

## Background

Oral squamous cell carcinoma (OSCC) is a major head and neck cancer and has negative effects on the quality of life and survival of patients.^1^ Although new diagnosis and therapeutic modalities have been developed, morbidity has tended to increase, and 5-year survival rates of 60–65% of the patients have not significantly improved over the past 30 years.^1,2^ A subpopulation of OSCC has high malignant potential and includes strongly invasive types and metastatic cases with a poor prognosis.^3^ To improve these patient outcomes, it is important to identify novel biomarkers for the diagnosis of metastasis and the elucidation of therapeutic targets in OSCC.

Gene expression is regulated by epigenetic mechanisms including DNA methylation and histone modifications, which determine cell fate and identity. Several drugs targeted to epigenetic regulatory factors have been developed, and they are in the initial clinical applications.^4^ Bromodomain containing 4 (BRD4) is a member of the bromodomain and extra-terminal (BET) family.^5^

Intensive studies of BRD4 revealed that it is an epigenetic regulator that facilitates transcriptional activities.^6,7^ It specifically recognises and binds to acetylated histone H3 lysine 27 (H3K27ac) in the enhancer and promoter regions of genes, where it recruits the mediator complex and histone modification enzymes.^8–10^ Therefore, it is considered as an epigenetic ‘reader protein’.^11^ Moreover, BRD4 interacts with positive transcription elongation factor b, phosphorylates the paused RNA polymerase II, and promotes transcriptional elongation.^12,13^ In several types of cancer, BRD4 is overexpressed and plays a key role in carcinogenesis and progression.^8,14^ It activates the transcription of oncogenes, including *c-MYC*, and is involved in cell proliferation.^8,15,16^

Specific BET inhibitors, including small molecule compounds that mimic the structure of acetylated lysine such as JQ1, have been developed as drugs to suppress tumour growth in several types of cancer.^7,17^ JQ1 binding to BRD4 prevents it from binding to acetylated histones, thus suppressing the excessive transcription of the oncogenes. However, the effect of JQ1 on OSCC cells is only beginning to be explored.^18,19^ Furthermore, the role of BRD4 and the inhibitory effect of BET inhibitors on cancer metastasis are generally poorly understood.^20,21^ Above all, the mechanism of the metastasis involving BRD4 in OSCC remains to be elucidated.

In this study, we show that JQ1 suppressed the proliferation and invasion of OSCC cell lines and also suppressed the BRD4-mediated transcriptional activation of a gene involved in metastasis, *matrix metallopeptidase 2* (*MMP2*). Consistently, we found that *BRD4* was upregulated in the OSCC specimens from cases with lymph node metastasis. This is the first demonstration of BRD4 regulation of a metastatic gene, and thus BRD4 may represent a prognostic and therapeutic target in OSCC.

## Methods

### Cell lines

Human OSCC cell line, HOC313, established from oral floor, was kindly provided by the Department of Oral and Maxillofacial Surgery, Graduate School of Medical Science, Kanazawa University (Ishikawa, Japan). Another human OSCC cell line, SAS, established from a human squamous cell carcinoma of the tongue,^22,23^ was obtained from the RIKEN BioResource Center (Ibaraki, Japan). The human OSCC cell line, OSC-19, was obtained from Kanazawa University (Ishikawa, Japan). OSC-19 cells were transfected with the pmR-ZsGreen1 (Takara Bio, Shiga, Japan) vector, and the cell line that stably expresses green fluorescent protein (GFP), OSC-19-GFP, was established. The human keratinocyte line, HaCaT, was kindly provided by Dr. Shirasuna, at Kyushu University (Fukuoka, Japan). The cells were cultured in Dulbecco's Modified Eagle Medium (DMEM; Sigma) supplemented with 10% foetal bovine serum (FBS; Corning) and maintained under a humidified 5% CO_2_ atmosphere at 37 °C.

### Reagents and antibodies

JQ1 (Abcam, ab141498, or Cayman Chemical, CAS:1268524-71-5) was diluted with dimethyl sulfoxide (DMSO; Wako) and used as a BET inhibitor. The following primary antibodies were used: anti-BRD4 (Bethyl Laboratories, A301-985A; dilutions used in immunoblotting (IB): 1:1000, chromatin immunoprecipitation (ChIP): 1:250), anti-β-tubulin (Sigma, T4026; dilution used in IB: 1:1000), anti-H3K27ac (Abcam, ab4729; dilution used in ChIP: 1:250), anti-H3K4me1 (Abcam, ab8895; dilution used in ChIP: 1:250), and anti-H3K4me3 (Abcam, ab8580; dilution used in ChIP: 1:250). The secondary antibodies used were ECL^TM^anti-rabbit immunoglobulin G (IgG; Sigma; dilution used in IB: 1:10,000) and ECL^TM^anti-goat IgG (Sigma; dilution used in IB: 1:4000).

### Cell proliferation assay

The cell proliferation assay was performed using a Cell Counting Kit-8 (CCK-8, Dojindo). Briefly, OSCC and HaCaT cells (2 × 10^3^ cells/100 µl) were seeded in 96-well plates, incubated at 37 °C for 24 h, and treated with various concentrations of JQ1 as indicated in the figures. The CCK-8 reagent was added to each well at a 1:10 dilution, and the plates were incubated for an additional 1–2 h at 37 °C. The absorbance of the samples was measured at 450 or 490 nm with a Microplate Reader (Bio-Rad). The IC_50_ values were calculated as the JQ1 concentrations causing 50% inhibition of cell growth.

### Scratch wound healing assay

The cell migration ability was determined using a scratch wound healing assay. HOC313 and SAS cells (5 × 10^4^ cells/ml) were seeded in 6-well plates and incubated at 37 °C until they were sub-confluent. The monolayered cells were wounded by scratching with pipette tips and incubated further at 37 °C in DMEM supplemented with 0.5% FBS for 24 and 18 h for HOC313 and SAS cells, respectively. Phase contrast images of the cells were captured at the time of the scratching and afterwards during the incubation, using a CKX53 microscope (Olympus) equipped with the CellSens standard program (v. 1.16). The degree of cell migration into the wounded area was calculated as the remaining space, using ImageJ v. 1.49.^24^

### Cell invasion assay

The cell invasion assay was performed using BioCoat Matrigel Invasion Chambers (24 wells, 8 µm pore size (Corning)). Briefly, HOC313 or SAS cells were seeded at a density of 5 × 10^4^ cells per chamber and cultured in serum-free DMEM inside the chamber. The outside of the chamber was filled with DMEM supplemented with 10% FBS. After 48 h, the cells were fixed and stained using a Diff-Quick Stain Kit (Sysmex). The stained membranes of the chambers were mounted on slides, covered, and observed with a BX51 microscope (Olympus) with the CellSens standard program (v. 1.16). Three fields of view were captured at random and analysed using ImageJ v. 1.49.^24^

### Colony-formation assay

HOC313 (1500 cells/well) and SAS cells (500 cells/well) were seeded in 6-well plates and cultured with DMSO or JQ1 for 10 days. The cells were fixed with 80% methanol for 20 min and stained with 0.1% crystal violet solution for 20 min. Colony images were captured by an Amersham Imager 680 (GE Healthcare), and their numbers were calculated with ImageJ v. 1.49.^24^

### Mouse studies

Female BALB/c‐nu/nu nude mice (4–6 weeks old) were purchased from Charles River Japan (Yokohama, Japan) and maintained at the Center for Animal Resources and Development of Kumamoto University. The mice were handled in accordance with the animal care policy of Kumamoto University. OSC-19-GFP cells were harvested and resuspended in DMEM, and then 5 × 10^5^ cells were injected into the mouse tongue. At the same time, an ALZET Osmotic Pump (Charles River, USA) was placed in the intraperitoneal space of each mouse to administer JQ1 (20 mg/kg/day) or DMSO for 2 weeks. The mice were dissected 1 week after the administration. Tumour volumes were measured by sequential calliper measurements of length (*L*) and width (*W*) and calculated using the formula: *LW*2*π*/6. Lymph node metastasis was identified as the GFP expression from the OSC19-GFP cells, using an SZX16 microscope (Olympus) equipped with the CellSens standard program (v. 1.9).

### Gene expression microarray analysis

The cRNA was amplified, labelled, and hybridised to a 60K Agilent 60-mer oligomicroarray, using an Agilent Low-Input QuickAmp Labeling Kit, one colour, according to the manufacturer’s instructions. All hybridised microarray slides were scanned by an Agilent scanner (SurePrint G3 Human Gene Expression Microarray 8×60K v3). Relative hybridisation intensities and background hybridisation values were calculated using the Agilent Feature Extraction Software (9.5.1.1).

Raw signal intensities and flags for each probe were calculated from hybridisation intensities (gProcessedSignal) and spot information (gIsSaturated, etc.), according to the procedures recommended by Agilent. (Flag criteria on GeneSpring Software. Absent (A): ‘Feature is not positive and significant’ and ‘Feature is not above background’. Marginal (M): ‘Feature is not Uniform’, ‘Feature is Saturated’, and ‘Feature is a population outlier’. Present (P): others.) The raw signal intensities of all samples were normalised by the quantile algorithm with the ‘preprocessCore’ library package^25^ in the Bioconductor software.^26^ We selected probes that call ‘P’ flag for at least one sample, excluding lincRNA probes. To identify upregulated or downregulated genes, we calculated the *Z*-scores^27^ and ratios (non-log scaled fold-change) from the normalised signal intensities of each probe for comparison between the control and experiment samples. We then established the criteria for regulated genes: (upregulated genes) *Z*-score ≥2.0 and ratio ≥1.5-fold, (downregulated genes) *Z*-score ≤−2.0 and ratio ≤0.66. The upregulated and downregulated genes are listed in Supplementary Tables 4 and 5, respectively.

For the Gene Ontology and Kyoto Encyclopedia of Genes and Genomes pathway analyses, the genes that were upregulated and downregulated by the JQ1 treatment were selected using the DAVID Bioinformatics Resources 6.8.^28,29^ In total, 159 genes related to OSCC metastasis were extracted from the gene set of Human Cancer Metastasis Database (HCMDB; http://hcmdb.i-sanger.com/index) for metastasis compiled by Zheng et al. and were plotted at the microarray expression levels.^30^

### Quantitative reverse transcription-PCR (qRT-PCR)

Total RNA was isolated from the patient biopsy specimens and the cultured cell lines with TRIzol reagents (Invitrogen) and reverse transcribed into cDNA using a Rever Tra Ace qPCR RT Kit (TOYOBO). Real-time quantitative PCR (qPCR) was performed with SYBR green fluorescence on an ABI Prism 7500 or Step One Plus system (Applied Biosystems). The comparative Ct (∆Ct) values of the target mRNAs were calculated as the fold changes relative to the *36B4* or *GAPDH* mRNA. Each experiment was performed at least three times. Primer sequences are listed in Supplementary Table 1.

### Immunoblotting

To prepare total cell lysates, the cells were dissolved in sodium dodecyl sulfate (SDS) sample buffer. Proteins were separated by SDS polyacrylamide gel electrophoresis and then transferred to a nitrocellulose membrane, Hybond-ECL (GE Healthcare). The membrane was blocked for 30 min in phosphate-buffered saline (PBS) containing 5% non-fat dry milk at room temperature and then incubated with primary antibodies (BRD4 and β-tubulin) in Solution 1 (TOYOBO) at 4 °C overnight. The membrane was washed with PBS containing 0.3% Tween 20 three times for 5 min each and then incubated with horseradish peroxidase-conjugated secondary antibodies in Solution 2 (TOYOBO) for 1 h at room temperature. After the membrane was washed with PBS containing 0.3% Tween 20 three times for 3 min each, specific protein bands were visualised by using the Western Lightning Plus-ECL reagent (PerkinElmer) and detected by an ImageQuant LAS 4000 image analyser (GE Healthcare).

### Patients and tissue specimens

This study used 36 initial OSCC biopsy specimens, each containing a pair of cancerous and non-cancer tissues (Table 1), obtained from patients who had undergone a tumour biopsy at the Kumamoto University Hospital, Kumamoto, Japan between 2012 and 2015. Table 1 provides the clinicopathological details of the patients. All tumours were staged according to the Tumour, Node, Metastasis classification in the eighth edition of the Union for International Cancer Control (2017). This study followed the guidelines of the Ethical Committee of Kumamoto University (project identification code: RINRI No. 1427). We explained the nature and aims of the research to these patients, and they provided the signed informed consents for participation in the study. All tissue samples were placed in sterile tubes, immediately frozen in liquid nitrogen, and stored at −80 °C until analysis.

**Table. 1:** Clinical and pathological characteristics of 36 OSCC patients.

Criteria	Number of patients (*n* = 36)
Age (years)	
<65	14
≥65	22
Sex	
Male	18
Female	18
Primary site	
Tongue	24
Gingiva	9
Others	3
T stage	
T1	5
T2	16
T3	7
T4	8
N stage	
* N* = 0	17
d.m. (delayed metastasis)	6
*N* > 1	13
Clinical stage	
I	5
II	11
III	7
IV	13

### Chromatin immunoprecipitation

ChIP assays were performed according to a modified version of the Upstate Biotechnology protocol, as described previously.^31^ Briefly, HOC313 and SAS cells were cross-linked with 1% formaldehyde for 5 (for histone modification) or 10 (for BRD4) min at room temperature. Twenty μl of Dynabeads M-280 Sheep Anti-Rabbit (Mouse) IgG (Thermo Fisher Scientific) were bound with 2 μg of antibody. Nuclei were isolated and disrupted with a Branson sonicator (3 times, 15 s, output; 70%), and chromatin was digested by 2000 gel units of Micrococcal nuclease (NEB M0247S) for 40 min at 37 °C or by sonication with a Bioruptor (20 times, 30 s ON/30 s OFF, output: high) (COSMO BIO) to generate 200–500 bp DNA fragments. The DNA fragments were incubated overnight at 4 °C with the magnetic beads bound with the antibody. The beads were washed, suspended in elution buffer with 0.25 mol/l NaCl, and incubated at 65 °C for 4 h to reverse the cross-links. The RNA and protein were digested using an RNase cocktail and Proteinase K, respectively, and the DNA was purified using a QIAquick PCR Purification Kit (Qiagen).

### ChIP-sequencing (ChIP-seq) and ChIP-qPCR

For ChIP-seq, ChIP DNA libraries for Illumina sequencing were prepared according to the NEBNext Ultra™ DNA Library Prep Kit for Illumina (NEB, E7370S) protocol. Amplified libraries were size-selected using AMPure XP Beads (Life Sciences) and E-Gel SizeSelect (Thermo Fisher Scientific) to capture 150 bp fragments. Libraries were quantified by qPCR using the KAPA Biosystems Illumina Library Quantification Kit, according to the manufacturer’s protocol. Libraries were sequenced on the Illumina NEXTSeq 500 sequencer for 75 bases in a single read mode. ChIP-seq analysis was performed using the Galaxy platform.^32^ The data were aligned to the human genome (build hg19, GRCh37) using BWA (v. 0.7.12),^33^ and PCR duplicates were removed using MarkDuplicates (Picard v. 1.136.0, http://broadinstitute.github.io/picard/). We used the MACS2 (v. 2.1.0)^34^ peak finding algorithm to identify the ChIP-enriched regions over the input DNA control, with the default parameter settings. A *q* value cut-off for broad/weak regions = 1.00e−01 was used for H3K27ac. We made TDF files normalised with read/million using the Integrative Genomics Viewer (IGV v 2.3.68)^35,36^ tools with parameters set to count -z 5 -w 25 -e 200 and visualised and compared the peak signals using IGV. The ChIP-seq data of other types of cancer were obtained from ENCODE Consortium^37^ and are listed in Supplementary Table 2.

For ChIP-qPCR, the DNA enrichment in the ChIP samples was measured by a qPCR analysis with the Step One Plus system (Applied Biosystems) and SYBR Green fluorescence. The threshold was set to cross a point where the PCR amplification was linear, and the cycle number required to reach the threshold was recorded and analysed using the MS Excel software program. PCR was performed using the precipitated DNA and the input DNA. Primer sequences are listed in Supplementary Table 3.

### Analysis of RNA-Seq

The RNA-seq data in Cancer Cell Line Encyclopedia (CCLE)^38^ were obtained from the Broad Institute data portal (https://portals.broadinstitute.org/ccle). The expression levels of *MMP2* and *BRD4* were obtained from the RNA-seq analyses of the PANC-1, HT-1080, HeLa, HepG2, MDA-MB-231, LNCaP, and MCF7 cell lines.

### Statistical analysis

Statistical analyses were performed with MS Excel and R (v. 3.4.0).^39^ Mean values ± SEM are representative of one of the three independent experiments. *P* values were calculated using unpaired two-tailed Student’s *t* tests. For the statistics of patient specimens, Wilcoxon signed-rank test, analysis of variance followed by Tukey–Kramer post hoc test, and a Pearson correlation analysis were used to validate the correlation. *P < 0.05, **P < 0.01, ***P < 0.001. A value of *P* < 0.05 was considered statistically significant.

## Results

### BET inhibitor JQ1 suppressed OSCC cell proliferation

**Fig. 1:**
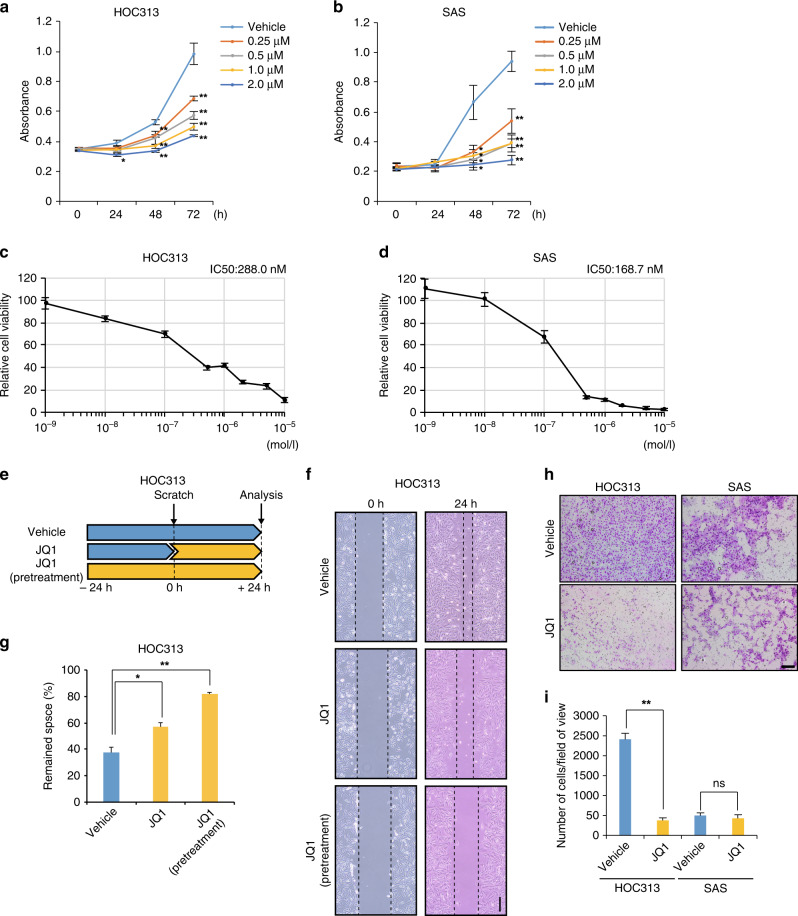
BET inhibitor JQ1 suppressed the proliferation, migration, and invasion of OSCC cells. **a**, **b** JQ1 suppressed the proliferation of OSCC cells. The growth of HOC313 (**a**) and SAS (**b**) cells treated with several concentrations of JQ1 was measured every 24 h. Cells that were treated with the drug solvent, DMSO (vehicle), served as a control. The number of cells was measured as the absorbance at 490 nm, using a CCK-8 kit. **c**, **d** Cell viability of the cells treated with JQ1 at the indicated concentrations for 72 h. The value for the control was set to 100. **e** JQ1 suppressed the migration of OSCC cells. Schematic illustration for the time course of the scratch wound healing assay. HOC313 cells treated with vehicle or JQ1 were incubated for 24 h, wounded by scratching, and incubated further for 24 h. **f** Representative microscopic images of the scratch wound healing assay. Dashed lines are the positions of the cells at the time of the scratch (0 h, left) and 24 h later (right). Scale bar, 200 µm. **g** Quantification of cell migration. The areas that were not healed at 24 h after the scratching were measured. **h** JQ1 suppressed the invasion of OSCC cells. Representative images of the cell invasion assay. The stained cells represent the cells that invaded through a Matrigel-coated membrane. HOC313 and SAS cells were treated with JQ1 and analysed at 48 h after incubation. Scale bar, 200 µm. **i** Quantification of cell invasions in **h**. The number of invaded cells per optical field was counted, using ImageJ. For **a**–**d**, **g**, and **i**, data are mean ± SEM from six (**a**, **b**) or three (**c**, **d**, **g**) independent experiments and three (**i**) independent views of the experiment. *P* values were calculated using the unpaired two-tailed Student’s *t* test (*P < 0.05, **P < 0.01, ns: not significant).

To investigate the effect of JQ1 on the proliferation of OSCC cells, we treated the two cell lines, HOC313 and SAS, with JQ1. HOC313 cells are highly invasive and have strong metastatic potential,^40^ while SAS cells have weak invasion ability.^41^ We also measured the effect of JQ1 on normal HaCaT cells (human keratinocyte). The JQ1 treatment suppressed the proliferation of the two OSCC cell lines significantly in time- and dose-dependent manners (Fig. 1a–d). JQ1 also lowered normal cell proliferation but to a lesser extent (Supplementary Fig. 1a, b). The IC_50_ values, which are the JQ1 concentrations causing 50% inhibition of cell growth, after 72 h treatment were 288.0, 168.7, and 320.1 nM for HOC313, SAS, and HaCaT cells, respectively (Fig. 1c, d, Supplementary Fig. 1b). Our colony-formation assay also demonstrated that the proliferation of HOC313 cells was less sensitive to JQ1, as compared to that of SAS cells (Supplementary Fig. 1c, d). These data demonstrated that JQ1 suppressed the proliferation of OSCC cells, as previously shown for other types of cancer cells.^42^

### JQ1 suppressed the migration of OSCC cells in vitro and in vivo

To investigate whether JQ1 suppresses the metastatic ability of OSCC, we assessed the impact of JQ1 treatment on the migration and invasion of HOC313 and SAS cells. First, we performed a scratch wound healing assay. In this assay, the degree of cell migration is measured as the fulfilment efficiency of the scratched area in monolayer cells. We found that the JQ1 treatment significantly suppressed the migration of HOC313 cells (Fig. 1e–g). Similar results, but with less efficiency, were obtained with SAS cells (Supplementary Fig. 1e–g).

To further explore the JQ1-mediated suppression of invasion, we performed a Matrigel invasion assay with HOC313 and SAS cells. The results showed that the JQ1 treatment for 48 h markedly suppressed the invasion of HOC313 cells but was less effective with SAS cells (Fig. 1h, i).

In principle, both the proliferation and cell migration abilities can contribute to these assays. However, we estimated that, in HOC313 cells, JQ1 primarily targets cell migration, because HOC313 cells are originally highly invasive,^40^ and JQ1 inhibited the proliferation of HOC313 cells less effectively than SAS cells (Fig. 1a–d and Supplementary Fig. 1c, d). Taken together, our results suggest that JQ1 affects not only the proliferation but also the metastatic potential of OSCC cells.

To explore the efficacy of JQ1 in vivo, we generated an orthotopic xenograft mouse model using the OSCC cell line, OSC-19, which was established from a metastatic tumour.^43,44^ GFP-expressing OSC-19-GFP cells were injected into the mouse tongue, and an osmotic pump that allows the continuous 2-week administration of DMSO or JQ1 was implanted in the abdominal cavity of the same mouse. After 3 weeks, the mice were analysed for tumour formation and lymph node metastasis in the head and neck (Supplementary Fig. 2a). The OSC-19-GFP xenograft tumours grew at the originally injected sites and tended to be inhibited by the JQ1 treatment (Supplementary Fig. 2b). More importantly, the tumour xenograft metastasised to the lymph nodes, which was detected as the GFP signal in the necks of mice treated with DMSO. This metastasis was reduced in the mice treated with JQ1 (Supplementary Fig. 2c).

These data suggest that JQ1 has a therapeutic effect by suppressing not only the proliferation but also the metastatic ability of the OSCC cells.

### JQ1 suppressed the expression of metastasis genes in HOC313 cells

Based on the observation that JQ1 suppressed the migration and invasion of HOC313 cells (Fig. 1), we sought to determine the genes with expression levels that are altered by the JQ1 treatment. We performed a microarray analysis and found that JQ1 downregulated 911 genes and upregulated 380 genes (Supplementary Fig. 3a and Supplementary Tables 4 and 5). Our ontology analysis revealed that the genes that were downregulated, but not up-regulated, with JQ1 are involved in cell migration regulation and pathways in cancer (Supplementary Fig. 3b, c). These results support the notion that JQ1 inhibits cancer metastasis in HOC313 cells.

**Fig. 2:**
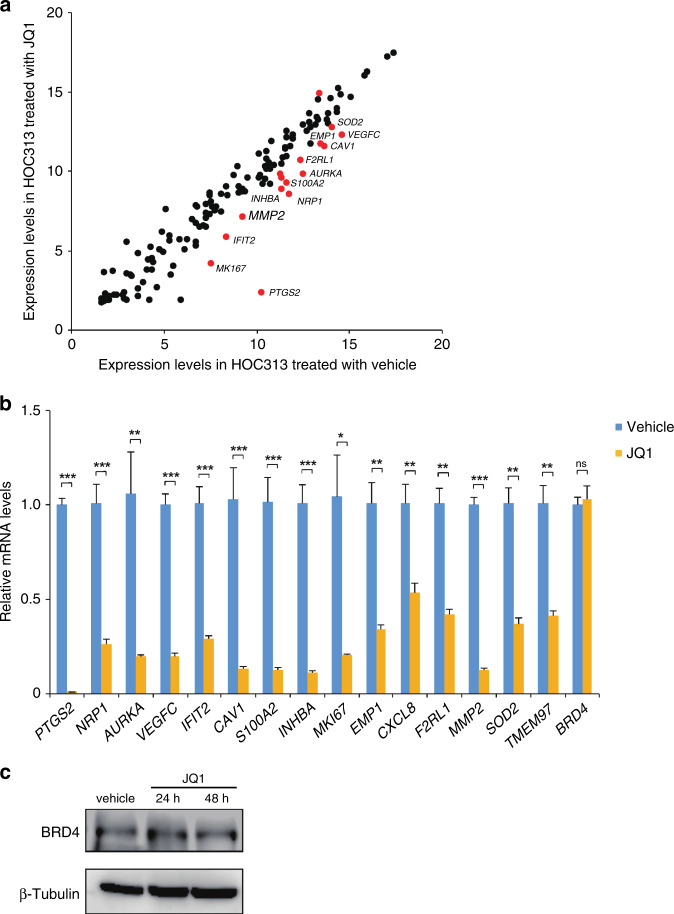
JQ1 suppressed expression levels of metastasis genes in OSCC cells. **a** JQ1 suppressed the genes involved in metastasis in HOC313 cells. Scatter plot showing the microarray analysis of mRNA expression levels of 159 genes involved in the metastasis of OSCC in HOC313 cells treated with JQ1 (1.0 μM for 48 h) or vehicle. Red dots indicate genes with *Z*-score ≥2.0 and ratio ≥1.5-fold or *Z*-score ≤−2.0 and ratio ≤0.66. **b** JQ1 repressed 15 genes involved in metastasis. The expression levels in HOC313 cells treated with JQ1 (1.0 μM for 48 h) were determined by qRT-PCR. The relative mRNA levels to non-treated cells are shown. The values were normalised to *36B4*. Data are mean ± SEM from three independent experiments. *P* values were calculated using the unpaired two-tailed Student’s *t* test (*P < 0.05, **P < 0.01, ***P < 0.001). **c** JQ1 did not affect the BRD4 protein levels. Immunoblotting showing the BRD4 protein levels in HOC313 cells treated with vehicle or JQ1 (1.0 μM for 24 and 48 h).

In parallel, we selected 159 genes that are involved in OSCC metastasis from the HCMDB^30^ and investigated their expression changes by JQ1, using our microarray data (Fig. 2a). As a result, we identified 15 metastasis-related genes with expression that was reduced with JQ1. Subsequently, qRT-PCR analyses confirmed that these genes were repressed by JQ1 (Fig. 2a, b). The *MMP2* gene was one of the genes that was most effectively inhibited by the JQ1 treatment, and its expression was reduced by about 90% in HOC313 cells (Fig. 2b). The MMP2 protein degrades the extracellular matrix during cancer invasion and metastasis, as well as during physiological development.^45–47^ Notably, the expression of the *BRD4* mRNA did not change upon JQ1 treatment (Fig. 2b). IB analysis also confirmed that the BRD4 protein levels did not change with the treatment (Fig. 2c). Collectively, BRD4 may facilitate the transcriptional activity of *MMP2* in OSCC cells and possibly support its invasive property.

### JQ1 reduced the BRD4 binding to the H3K27ac sites in the *MMP2* locus

**Fig. 3:**
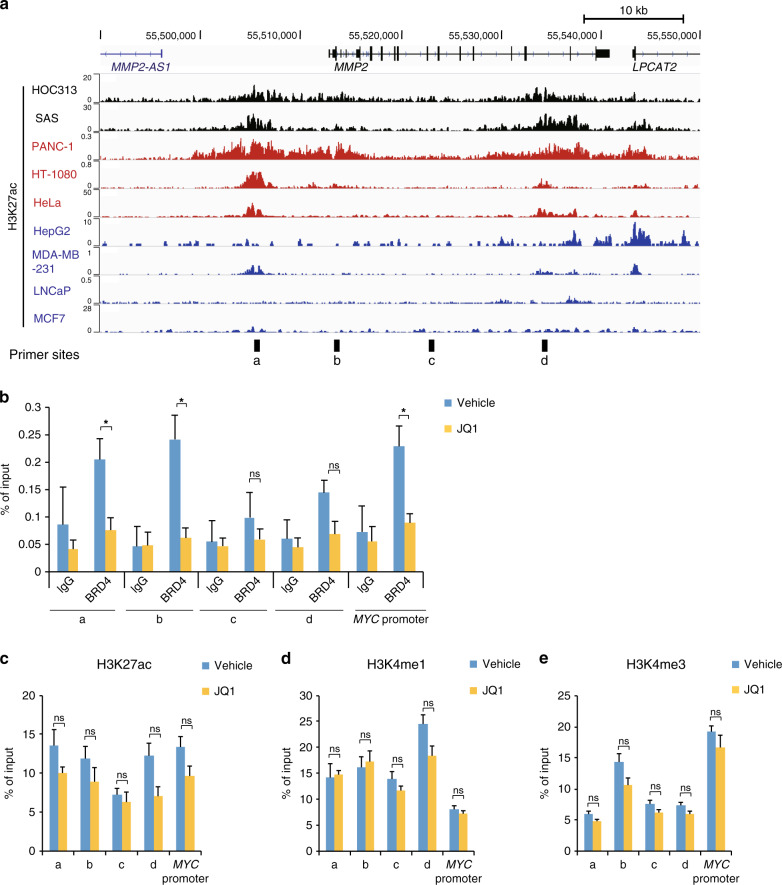
JQ1 inhibited BRD4 binding to the H3K27ac-enriched sites at the *MMP2* locus. **a** The genome browser (hg19) snapshots, showing the H3K27ac ChIP-seq signals at the *MMP2* locus (chr16: 55,490,000–55,549,999) in HOC313 and SAS cells and other types of cancer cell lines (PANC-1: pancreatic cancer,^37^ HT-1080: sarcoma,^65^ HeLa: cervical cancer,^37^ HepG2: liver cancer,^37^ MDA-MB-231: breast cancer,^66^ LNCaP: prostate adenocarcinoma,^67^ MCF7: breast cancer^37^). The *MMP2* expression level in each cell line was analysed (Supplementary Fig. 4a, *MMP2* expression) and indicated as red (high) or blue (low) coloured cell line name. Black bars at the bottom show the sites for the PCR primers used in **b**–**e**. **b** ChIP-qPCR, showing BRD4 binding to the *MMP2* locus in HOC313 cells treated with JQ1 (1.0 μM for 24 h) or vehicle. As a negative control, rabbit IgG was used for the ChIP assay. **c**–**e** ChIP-qPCR, showing the relative levels of the indicated histone modifications at the *MMP2* locus in HOC313 cells treated with JQ1 (1.0 μM for 24 h) or vehicle. The positions of the PCR sites (a–d) are displayed in **a**. The *MYC* promoter served as a positive control, representing an H3K27ac-enriched site (Supplementary Fig. 4b). For **b**–**e**, data are mean ± SEM from three independent experiments. *P* values were calculated using the unpaired two-tailed Student’s *t* test (*P < 0.05, ns: not significant).

The reduction of the *MMP2* gene expression by JQ1 prompted us to investigate whether JQ1 inhibits BRD4 binding to H3K27ac at the *MMP2* locus in OSCC cells. We first performed ChIP-seq using HOC313 and SAS cells to identify the genomic sites with this histone modification. We found several regions around the *MMP2* locus that were enriched in H3K27ac, including the transcription start site (TSS), the promoter, and upstream sites (Fig. 3a). The published ChIP-seq data of H3K27ac in other types of cancer cell lines showed both common and un-common peaks with those in HOC313 cells. According to the RNA-seq data from CCLE,^38^ we evaluated the expression levels of *MMP2* and *BRD4* in these cell lines. There were no significant differences in the *BRD4* expression levels among them; however, the *MMP2* expression level differed depending on the cell line (Supplementary Fig. 4a). The cancer cell lines with high *MMP2* expression (coloured in red) showed more enrichment of H3K27ac at the *MMP2* locus, as compared to those with low *MMP2* expression (coloured in blue) (Fig. 3a and Supplementary Fig. 4a). These results suggest that the *MMP2* gene is regulated by H3K27ac, possibly through BRD4 binding.

We then examined whether BRD4 actually binds to the H3K27ac-enriched sites in the *MMP2* locus. We treated HOC313 cells with JQ1 and performed ChIP-qPCR. We measured the BRD4 binding to the upstream (a), TSS/promoter (b), and gene body (c, d) regions of the *MMP2* locus (Fig. 3b). We used the *MYC* promoter as a positive control, as it is highly enriched in H3K27ac and likely bound by BRD4 (Supplementary Fig. 4b).^8,16,48,49^ We found that BRD4 preferentially bound to the upstream (a) and TSS/promoter (b) sites of *MMP2* in HOC313 cells (Fig. 3b, blue bars). The JQ1 treatment significantly reduced BRD4 binding to these regions (Fig. 3b, yellow bars**)**. Since the (a) site is commonly enriched with H3K27ac among cancers with high *MMP2* expression (Fig. 3a, Supplementary Fig. 4a), and it is about 10 kb upstream of the promoter region, we consider the (a) site to be the *MMP2* enhancer. It should be noted that the levels of H3K27ac, histone H3 lysine 4 mono-methylation (H3K4me1), and histone H3 lysine 4 tri-methylation (H3K4me3), which are not implicated in BRD4 binding, did not change significantly upon the JQ1 treatment (Fig. 3c–e). Collectively, our study showed that JQ1 inhibited BRD4 binding to the regulatory *cis*-elements with the H3K27ac modification in the *MMP*2 locus (Fig. 3), leading to the repression of *MMP2* transcription (Fig. 2). We suggest that cell migration and invasion are suppressed by JQ1 via the epigenetic regulation in highly invasive OSCC.

### *BRD4* and *MMP2* expression levels were correlatively elevated in patients with lymph node metastasis

**Fig. 4:**
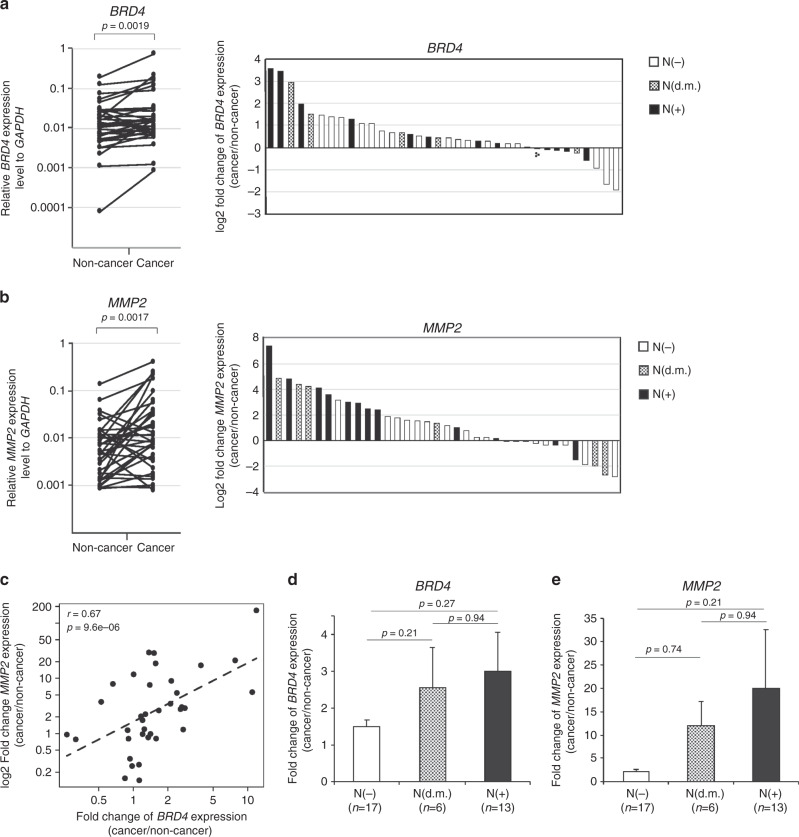
*BRD4* and *MMP2* are highly expressed in OSCC patients with metastasis. **a**, **b** *BRD4* and *MMP2* are highly expressed in the cancer regions of OSCC tissues. qRT-PCR analyses showing the expression levels of *BRD4* (**a**) and *MMP2* (**b**) mRNAs in cancer and non-cancer tissues collected from OSCC patients (*n* = 36) are on the left. Fold changes (cancer/non-cancer regions) in individual specimens are on the right. The specimens were derived from OSCC patients without (N(−)) or with (N(+)) lymph node metastasis or with delayed lymph node metastasis (N(d.m.)). **c** The expression of *BRD4* and *MMP2* is correlated in OSCC patient-derived cells. Scatter plots showing the fold change of *BRD4* and *MMP2* mRNA expression (cancer/non-cancer regions) in each specimen. **d**, **e** *BRD4* and *MMP2* are highly expressed in lymph node metastases. qRT-PCR analyses showing the fold change of *BRD4* and *MMP2* mRNA expression (cancer/non-cancer regions) in the indicated OSCC specimens, OSCC without (N(−)) or with (N(+)) lymph node metastasis or with delayed lymph node metastasis (N(d.m.)). The correlations (*r* values) and *P* values were calculated using Pearson’s correlation coefficient test. For **a**, **b**, *P* values were calculated using the Wilcoxon signed-rank test.

To clarify whether BRD4 is associated with OSCC, as in many other cancers,^14,21^ we performed qRT-PCR and examined the *BRD4* expression levels in tissues from biopsy specimens of OSCC patients (Table 1). The level of *BRD4* expression was increased in the cancerous regions, as compared to the non-cancer regions, within each individual patient (Fig. 4a). As previously reported,^50^ the *MMP2* expression level was also significantly increased in OSCC tissues (Fig. 4b). Consistently, our correlation analyses confirmed that the normalised *BRD4* and *MMP2* expression levels were correlated in cancer tissues but not in non-cancer tissues (Fig. 4c and Supplementary Fig. 5a, b). These results suggest that *BRD4* might play a role in the activation of the *MMP2* gene, which is involved in the metastasis of OSCC.

To explore the role of *BRD4* in OSCC metastasis, we classified the patients into groups with and without lymph node metastasis at the time of the initial diagnosis and treatment. We further classified the patient group into the one who did not exhibit metastasis at the initial diagnosis and treatment but did at a subsequent diagnosis, representing delayed metastasis. Interestingly, our qRT-PCR analysis demonstrated that the *BRD4* expression level was slightly higher in the cancerous tissues with lymph node metastasis (N(+)) than in those without metastasis (N(−)) (Fig. 4d). Furthermore, both *BRD4* and *MMP2* were highly expressed, not only in the cancerous tissues of patients with lymph node metastasis (N(+)) but also in cases with delayed metastasis (N(d.m.)) (Fig. 4e). In contrast, when we subdivided the patients according to other characteristics, including T stage (T1–T4) or clinical stage (I–IV), there were no differences (Supplementary Fig. 5c–f). These findings suggest that the high expression levels of *BRD4* and *MMP2* are specifically correlated with OSCC metastasis and thus can serve as a predictive biomarker.

## Discussion

**Fig. 5:**
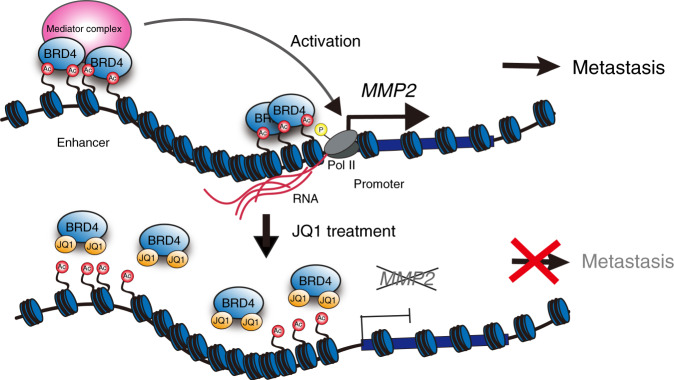
Models of OSCC metastasis through regulation of the *MMP2* locus with BRD4 binding. In OSCC cells highly expressing *MMP2*, the *MMP2* gene is activated by BRD4 binding to the promoter and enhancer, which are enriched with H3K27ac. BRD4 may recruit regulatory factors, including the mediator complex and HATs. The BET inhibitor JQ1 competes with BRD4 and reduces the *MMP2* transcription that is regulated by the H3K27ac accumulation but not by the BRD4 expression (Figs. 2b and 3 and Supplementary Fig. 4a).

Metastasis is part of tumour progression and involves several events, including gaining of cellular motility and invasion abilities, as well as the epithelial-to-mesenchymal transition (EMT) and angiogenesis. The elucidation of the molecular mechanism involving epigenetic regulation in OSCC metastasis has long been awaited,^18–21,46,47^ because it is well correlated with a poor prognosis.^3^ In this study, we demonstrated that the BET inhibitor JQ1 repressed the *MMP2* gene, one of the critical genes for metastasis, in OSCC cells (Fig. 2). JQ1 prevented BRD4 from binding to the enhancer and promoter of the *MMP2* locus, which are enriched with H3K27ac (Fig. 3). JQ1 also suppressed the proliferation, migration, and invasion of OSCC cells (Fig. 1 and Supplementary Figs. 1 and 2). Therefore, we propose that BRD4 contributes to the metastasis of these cells by activating the *MMP2* expression, through binding to the *cis*-elements in the *MMP2* gene and recruiting histone modifiers and/or mediator complexes for the gene activation (Fig. 5, see below).

Although many studies have investigated the transcriptional regulation of metastasis-related genes, this study is the first to demonstrate the detailed epigenetic mechanism in OSCC. MMPs are associated with EMT in many types of cancer,^46^ including OSCC.^45,50,51^ Overexpression of MMPs is one of the metastasis biomarkers. There are several subtypes of MMPs. Among them is the gelatinase group (MMP2, MMP9), which selectively cleaves type IV basement membrane collagen, leading to the promotion of cell proliferation, invasion, and metastasis.^46^ The epigenetic regulation of *MMP2* in OSCC, as shown in this and other studies, including DNA methylation at the promoter region and histone modifications, was shared with other cancers.^52–54^

*BRD4* expression was significantly correlated with *MMP2* expression in cancerous areas, but not in non-cancerous regions, among the OSCC patients (Supplementary Fig. 5a, b). Furthermore, *BRD4* was highly expressed in OSCC patients with lymph node metastasis, including patients who exhibited metastasis after the initial diagnosis (Fig. 4). These findings suggest that BRD4 can serve as a therapeutic target and diagnostic marker for the metastatic potential of OSCC.

It has been postulated that BRD4 functions as a foothold that binds to H3K27ac, recruits a mediator complex containing histone acetyltransferases (HATs), and further increases the H3K27ac level. This positive feedback then establishes super-enhancer regions enriched with H3K27ac for many genes.^55,56^ This phenomenon may also occur for the *MMP2* gene in OSCC (Fig. 5), since our ChIP-qPCR data showed slight but consistent declines of the H3K27ac levels by the JQ1 treatment at multiple regions in the *MMP2* locus (Fig. 3c). This implied that the inhibition of the BRD4 binding can result in the reduction of the H3K27ac level.

Consistently, our combined analyses of the RNA-Seq and H3K27ac ChIP-seq data for *MMP2* showed varied degrees of *MMP2* expression among several types of cancer cell lines, despite the equally high expression of *BRD4* among them (Fig. 3a and Supplementary Fig. 4a). The accumulation of H3K27ac at the *MMP2* locus may directly influence it, as it may be critical for the *MMP2* gene activation in which BRD4 binds to the locus, recruits HATs, and establishes the positive feedback loop for H3K27ac enrichment at the locus (Fig. 5).

BRD4 has been implicated in several aspects of cancer. Previous reports have shown that BRD4 induces the overexpression of oncogenes, such as c-*MYC*, to promote cell proliferation.^8,15,16^ The expression of c-*MYC* and *FOSL1* was suppressed by JQ1 through the inhibition of BRD4 binding to the acetylated histone.^8,16,48,49,57^ A few studies have shown the role of BRD4 in cancer metastasis.^18–21^ Wang et al. reported that BRD4 induces cell migration and invasion in hepatocellular carcinoma cells, by increasing the expression levels of MMP2 and MMP9 via the SHH signalling pathway.^47^ In the present study, we showed that JQ1 did not suppress *BRD4* expression (Fig. 2b) but instead inhibited BRD4 accumulation at the H3K27ac-enriched sites at the *MMP2* locus (Fig. 3b). This is the first demonstration of the critical epigenetic regulatory mechanism of BRD4 at the *MMP2* locus in OSCC.

HIF-1α, SOX2, E-cadherin, vimentin, MALAT1, TP53, and NOTCH1 have been reported as prediction markers for head and neck cancer. However, these are only a few prediction markers, as compared to those for other cancers, and they have not worked well for early diagnosis, prognosis, and individualised treatments.^58^ In this study, we found BRD4 as a possible marker for not only tumour growth but also metastasis (Fig. 4). In agreement with this, the *BRD4* expression level correlated with the prognoses in head and neck cancer, ovarian cancer, and urothelial carcinoma of the bladder.^21,59,60^ BRD4 may regulate various genes, including those reported as biomarkers, such as *HIF1A*, *VIM*, *MALAT1*, *TP53*, and *NOTCH1*. Our ChIP-seq analysis of HOC313 cells confirmed the enrichment of H3K27ac at these loci (data not shown). Genome-wide and single-cell analyses of the H3K27ac distribution with each patient may pave the way toward individualised treatments.

As shown in Fig. 2b, JQ1 suppressed the expression of *MMP2*. Although there are >50 MMP inhibitors, they are not applicable for clinical cancer patients due to their side effects.^61^ The JQ1 treatment also had a slightly toxic effect on normal cells (Supplementary Fig. 1a, b). This may be inevitable, because JQ1 also inhibits the cell cycle.^16,48,49^ Therefore, a combination therapy using JQ1 and an MMP inhibitor at lower doses may reduce the side effects. As another example, the combination of JQ1 and a HAT inhibitor may have an additive and stronger effect than the single agent treatment. The combination of JQ1 with other epigenetic therapeutic drugs, including DNMT, HDAC, LSD1, and EZH2 inhibitors, may also be more potent than the single treatment.^62^ It is noteworthy that the major difference between these epigenetic drugs and the BET inhibitors is that the BET inhibitors have the advantage of modulating gene expression without editing histone modifications. Furthermore, BET inhibitors reportedly suppress the expression of genes involved in immune checkpoints, and the combination of a PD-1 monoclonal antibody drug and JQ1 was effective in Eμ-Myc lymphoma mice.^63,64^ Since immunotherapy is performed in the treatment of head and neck cancer, the combination with a BET inhibitor may have further beneficial effects.

Collectively, our analyses revealed an epigenetic regulatory mechanism of BRD4 at the *MMP2* locus and showed that JQ1 inhibited the metastasis of OSCC. Thus BRD4 may represent a new therapeutic target and prognostic marker for OSCC.

## Supplementary information

Supplementary information

## Data Availability

Our original ChIP-seq data sets have been deposited in the DNA Data Bank of Japan (DDBJ) Sequence Read Archive with the accession number DRA005541. Our Microarray data sets have been deposited in the NCBI with the accession number GSE140858. The ChIP-seq data sets used in this work are publicly available.^37^ Other data that support the findings of this study are available from the corresponding author upon reasonable request.
