# Supplementary material for: BRD4 promotes metastatic potential in oral squamous cell carcinoma through the epigenetic regulation of the MMP2 gene
Source: Br J Cancer. 2020 Jun 5;123(4):580–90. doi: 10.1038/s41416-020-0907-6 (PMC7435185; doi:10.1038/s41416-020-0907-6)
Supplement: Supplementary file 1 — Supplementary information [file 41416_2020_907_MOESM1_ESM.pdf]

Supplementary information

BRD4 promotes metastatic potential in oral squamous cell carcinoma through the epigenetic regulation of the *MMP2* gene

Figure Legends

Supplementary Figure 1. JQ1 suppressed OSCC cell proliferation and migration

(a) Effects of JQ1 on the proliferation of HaCaT normal keratinocyte cells. Proliferation of the cells treated with JQ1 at the indicated concentrations was measured every 24 h. The number of cells was measured as the absorbance at 450 nm, using a CCK-8 kit. **(b)** Cell viability of HaCaT cells treated with JQ1 at the indicated concentrations for 72 h. The value for the control was set to 100. **(c, d)** Colony formation assays of HOC313 **(c)** and SAS **(d)** cells. JQ1 suppressed cell proliferation in a dose-dependent manner. The relative numbers of colonies are shown in each graph. The colony number for the vehicle (DMSO) treatment was set to 1. **(e)** Schematic illustration for the time course of the scratch wound healing assay. **(f)** Representative microscopic images of the scratch wound healing assay. SAS cells treated with vehicle or JQ1 are shown. Dashed lines are the positions of the cells at the time of the scratch (0 h, left) or 18 h later (right). Scale bar, 200 μm. **(g)** Quantification of SAS cell migration in **(f)**. The areas that were not healed at 18 h after the scratch were measured. For **(a), (b), (c), (d)** and **(g)**, data are mean ± SEM from three independent experiments. *P*-values were calculated using the unpaired two-tailed Student's t-test (**P* < 0.05, ***P* < 0.01).

Supplementary Figure 2. JQ1 suppressed OSCC cell growth and metastasis *in vivo*

(a) Time course of the mouse orthotopic xenograft experiment. GFP-expressing OSC-19 (OSC-19-GFP) cells were injected into the mouse tongue, and at the same time, an osmotic pump (ALZET Osmotic Pump) that allows the continuous 2-week administration of DMSO or JQ1 was implanted in the abdominal cavity. After three

weeks of these treatments, the mice were analyzed for tumor formation (**b**) and metastasis of the OSC-19-GFP cells (**c**). **(b)** JQ1 suppressed tumor growth. Representative OSC-19-GFP xenograft tumors treated with DMSO or JQ1. Scale bar, 5 mm. Data are mean ± SEM from four mice. **(c)** JQ1 suppressed lymph node metastasis. OSC-19-GFP cells that were originally injected in the tongue and grown in the oral cavity were detected in each mouse (shown with *, left). In addition, their lymph node metastases were detected as the GFP signals in the neck (arrows, left). The frequencies of the lymph node metastases (2 possible metastasis sites in each mouse neck) were reduced with the JQ1-treatment (right). Data are from four mice.

Supplementary Figure 3. JQ1 suppressed the expression levels of *BRD4* and *MMPs* in OSCC cells

(a) JQ1 affected RNA expression in HOC313 cells. The MA-plot from the microarray expression analysis identified 380 up-regulated and 911 down-regulated genes by the JQ1 treatment (1.0 μM for 48 h). Red dots indicate up-regulated genes: Z-score ≥ 2.0 and ratio ≥ 1.5-fold, and down-regulated genes: Z-score ≤ -2.0 and ratio ≤ 0.66. The up- and down-regulated genes are listed in **Supplementary Tables 4 and 5**, respectively. **(b)** Gene ontology enrichment analysis for sets of genes that were down- (left) and up-regulated (right) by the JQ1 treatment. The bar graph shows the enrichment score obtained from DAVID Bioinformatics Resources. Cell migration regulating genes were suppressed by the JQ1 treatment. **(c)** KEGG pathway analysis for genes that were down- (left) and up-regulated (right) by the JQ1 treatment. The bar graph shows the P values obtained from DAVID Bioinformatics Resources. JQ1 affected the Pathways in cancer, which include the *MMP2* gene.

Supplementary Figure 4. JQ1 inhibited BRD4 binding to the H3K27ac-enriched sites in the *MMP2* locus

(a) Analysis of the expression levels of *MMP2* and *BRD4*, based on the RNA-seq data obtained from CCLE[1]. The *MMP2* expression levels were classified into "high" (colored in red) or "low" (colored in blue). ChIP-seq profiles of H3K27ac at the *MMP2* locus in these cell lines are shown in **Fig. 3a**. **(b)** Genome browser (hg19) snapshots, showing H3K27ac ChIP-seq signals at the *MYC* locus (chr16:128,730,000-128,780,000) in HOC313 and SAS cells, and other types of cancer cell lines. The black bar at the bottom shows the sites for the PCR primers used in **Fig. 3b-e**.

Supplementary Figure 5. *BRD4* and *MMP2* are highly expressed in OSCC tissues

(a, b) Expression levels of *BRD4* and *MMP2* were correlated in cancer tissues. Scatter plots showing the *BRD4* and *MMP2* expression levels in cancer **(a)** and non-cancer **(b)** tissues in each specimen. The correlations (r-values) and *P*-values were calculated using Pearson's correlation coefficient test. **(c-f)** qRT-PCR analyses showing the fold changes of *BRD4* **(c, d)** and *MMP2* **(e, f)** mRNA expression in cancer, as compared to non-cancer (Cancer / Non-cancer) tissue specimens. The OSCC patients were classified according to the T-stage (T1-4) **(c, e)** and clinical stage (I-IV) **(d, f)**.

Supplementary references

1. The Cancer Cell Line Encyclopedia C, Stransky N, Ghandi M, Kryukov GV, Garraway LA, Lehár J *et al.* Pharmacogenomic agreement between two cancer cell line data sets. *Nature* 2015; **528**: 84

2. Consortium EP. An integrated encyclopedia of DNA elements in the human genome. *Nature* 2012; **489**(7414): 57-74

3. Kelsey AD, Yang C, Leung D, Minks J, Dixon-McDougall T, Baldry SEL *et al.* Impact of flanking chromosomal sequences on localization and silencing by the human non-coding RNA XIST. *Genome biology* 2015; **16**: 208-208

4. Rhie SK, Hazelett DJ, Coetzee SG, Yan C, Noushmehr H, Coetzee GA. Nucleosome positioning and histone modifications define relationships between regulatory elements and nearby gene expression in breast epithelial cells. *BMC genomics* 2014; **15**(1): 331-331

5. Hazelett DJ, Rhie SK, Gaddis M, Yan C, Lakeland DL, Coetzee SG *et al.* Comprehensive functional annotation of 77 prostate cancer risk loci. *PLoS genetics* 2014; **10**(1): e1004102-e1004102

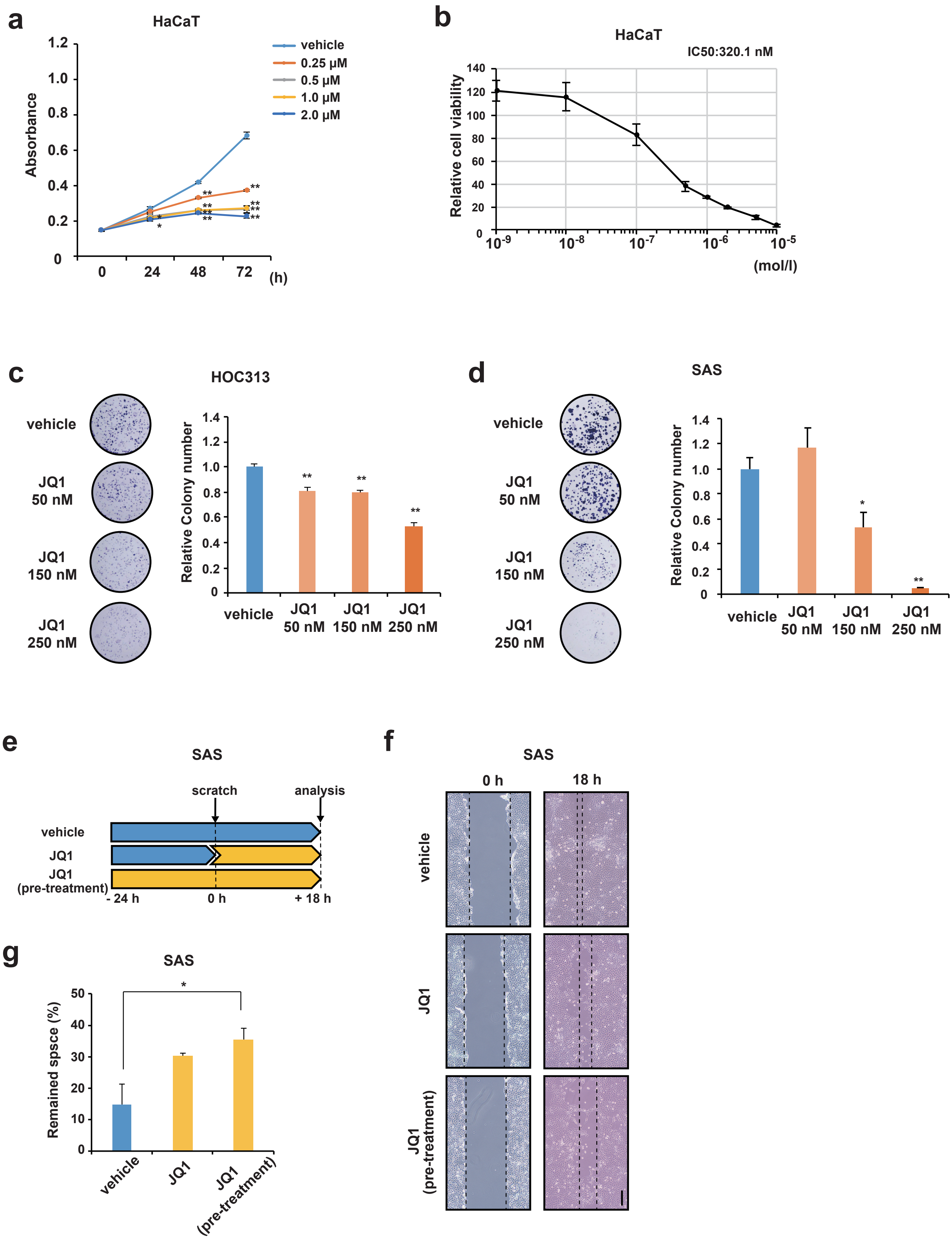

Yamamoto et al. Supplementary Figure 1

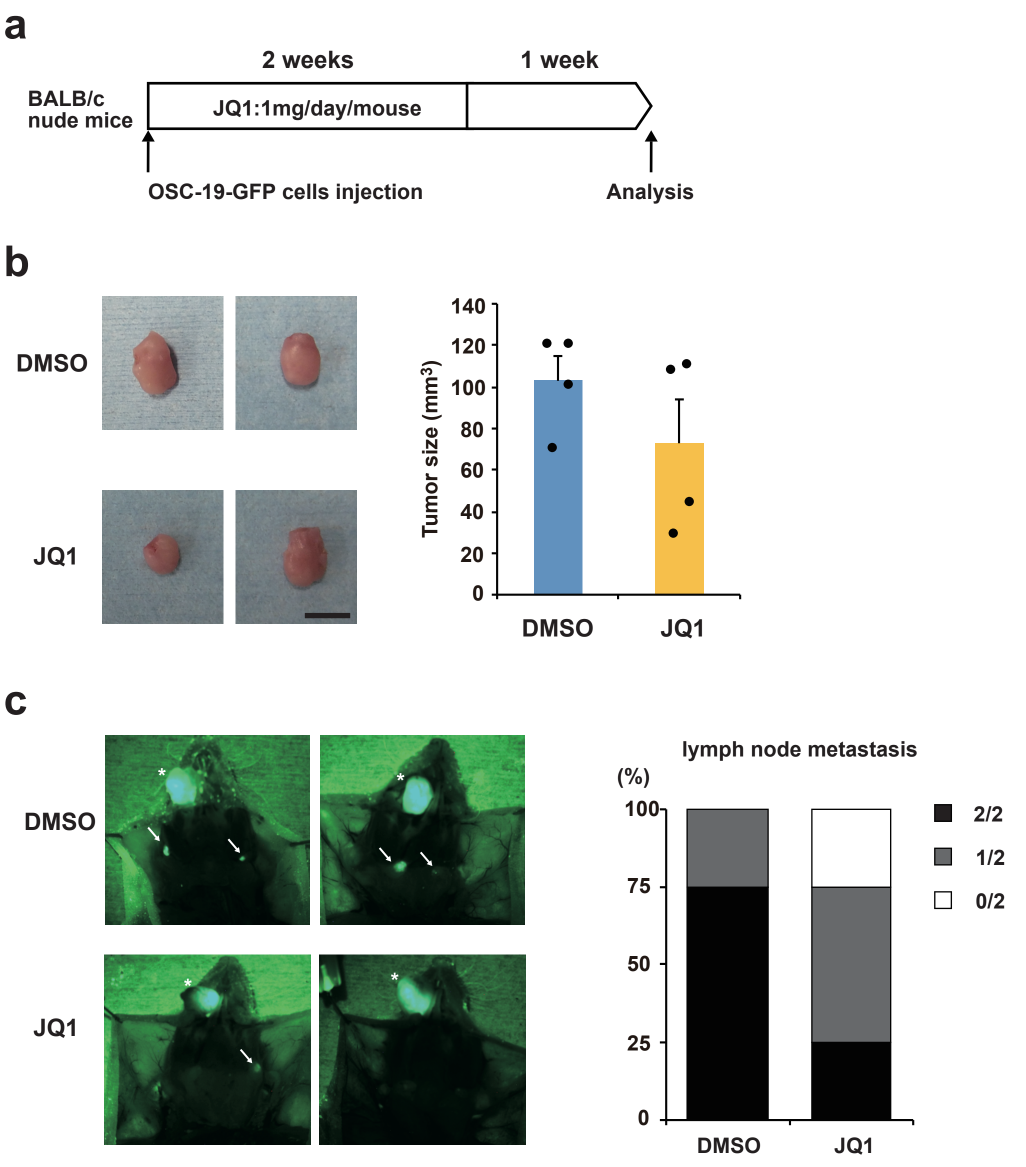

Yamamoto et al. Supplementary Figure 2

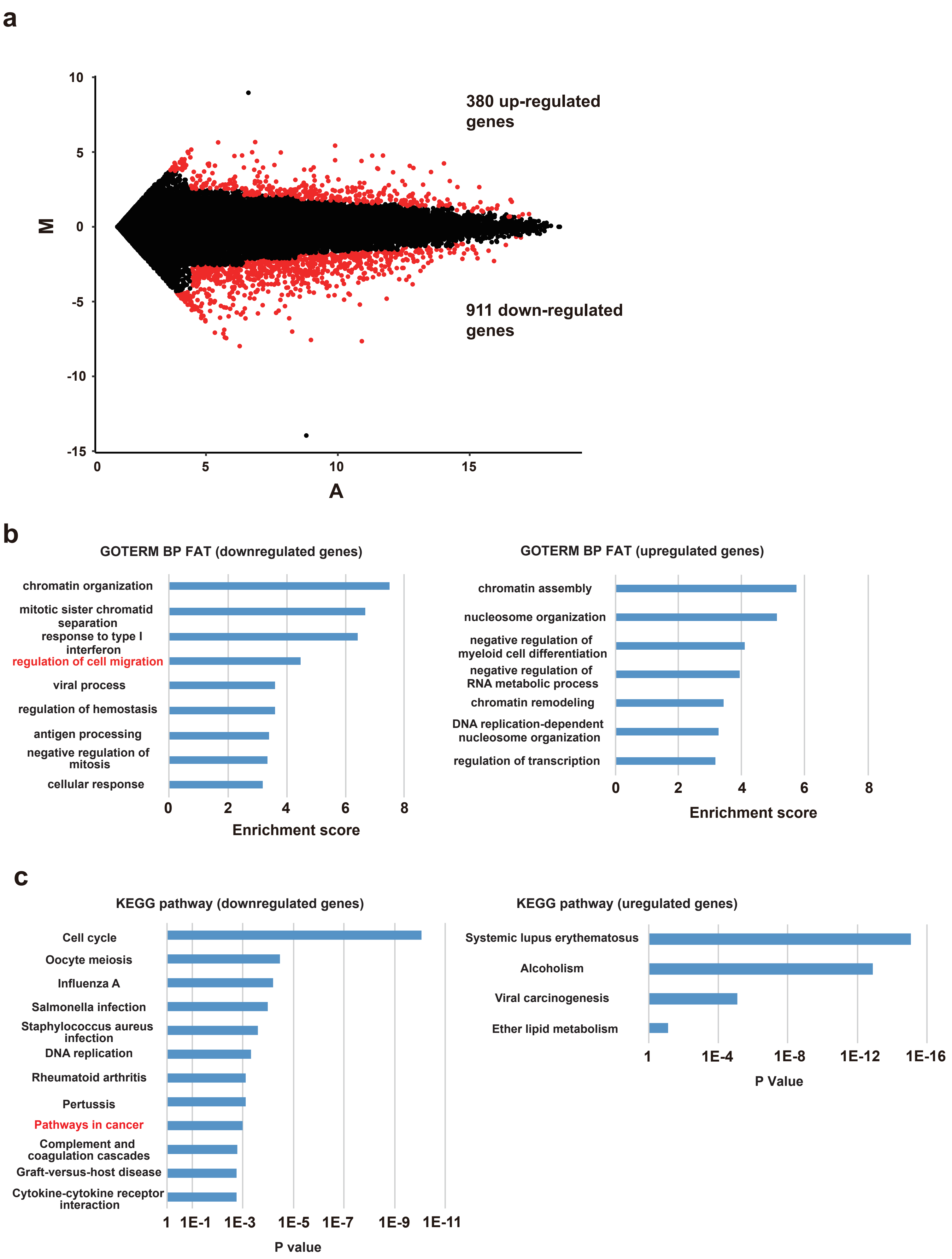

Yamamoto et al. Supplementary Figure 3

a

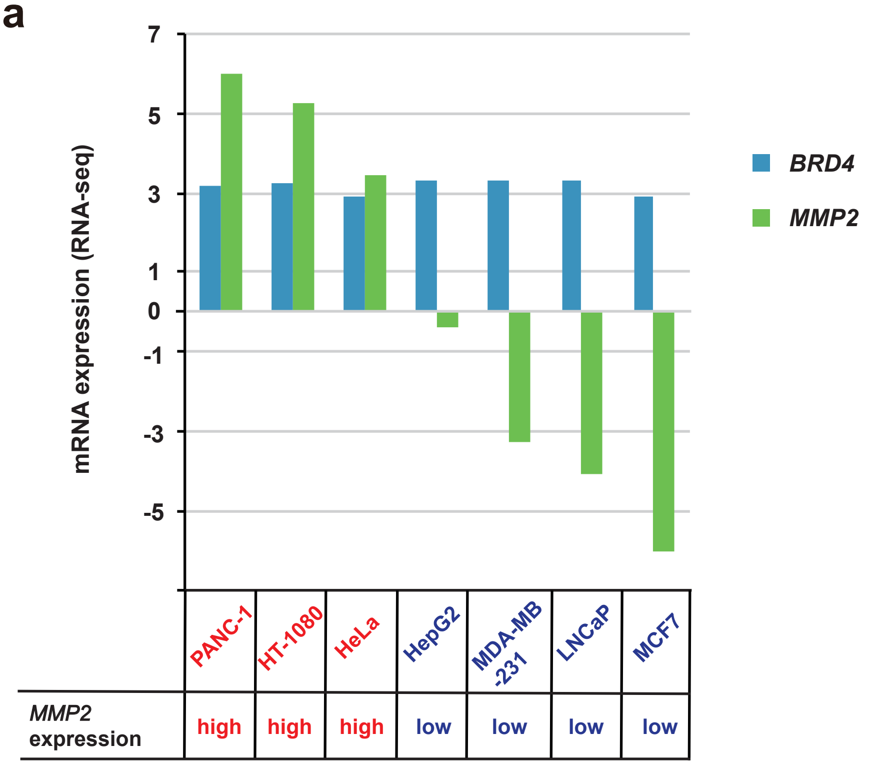

b

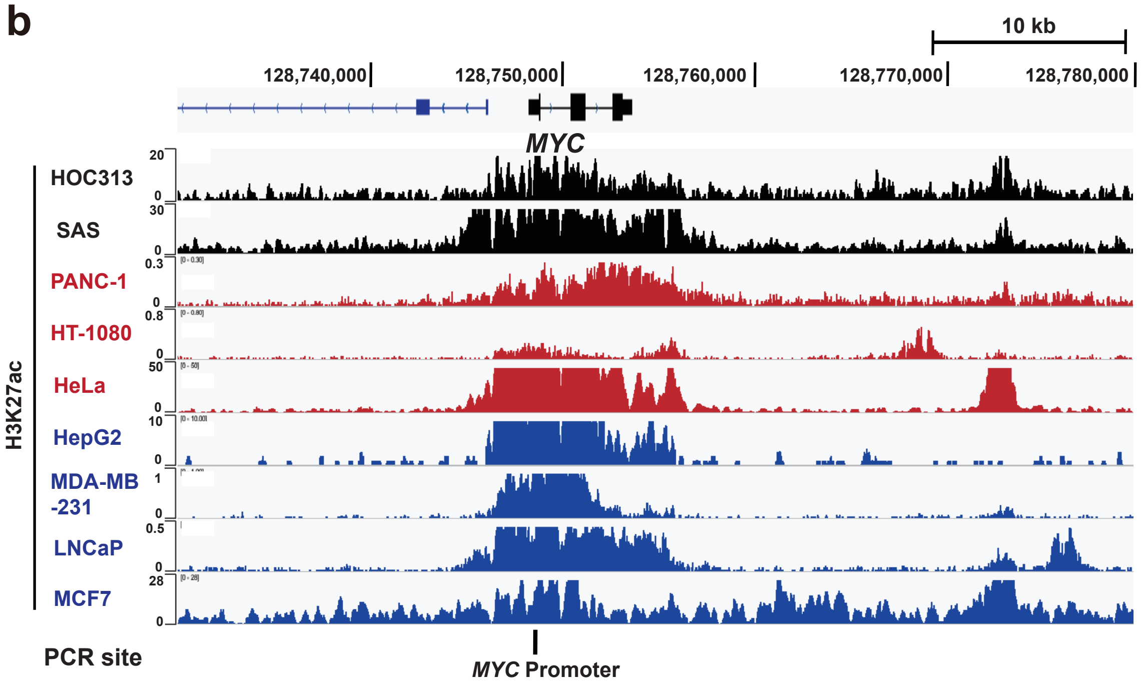

Yamamoto et al. Supplementary Figure 4

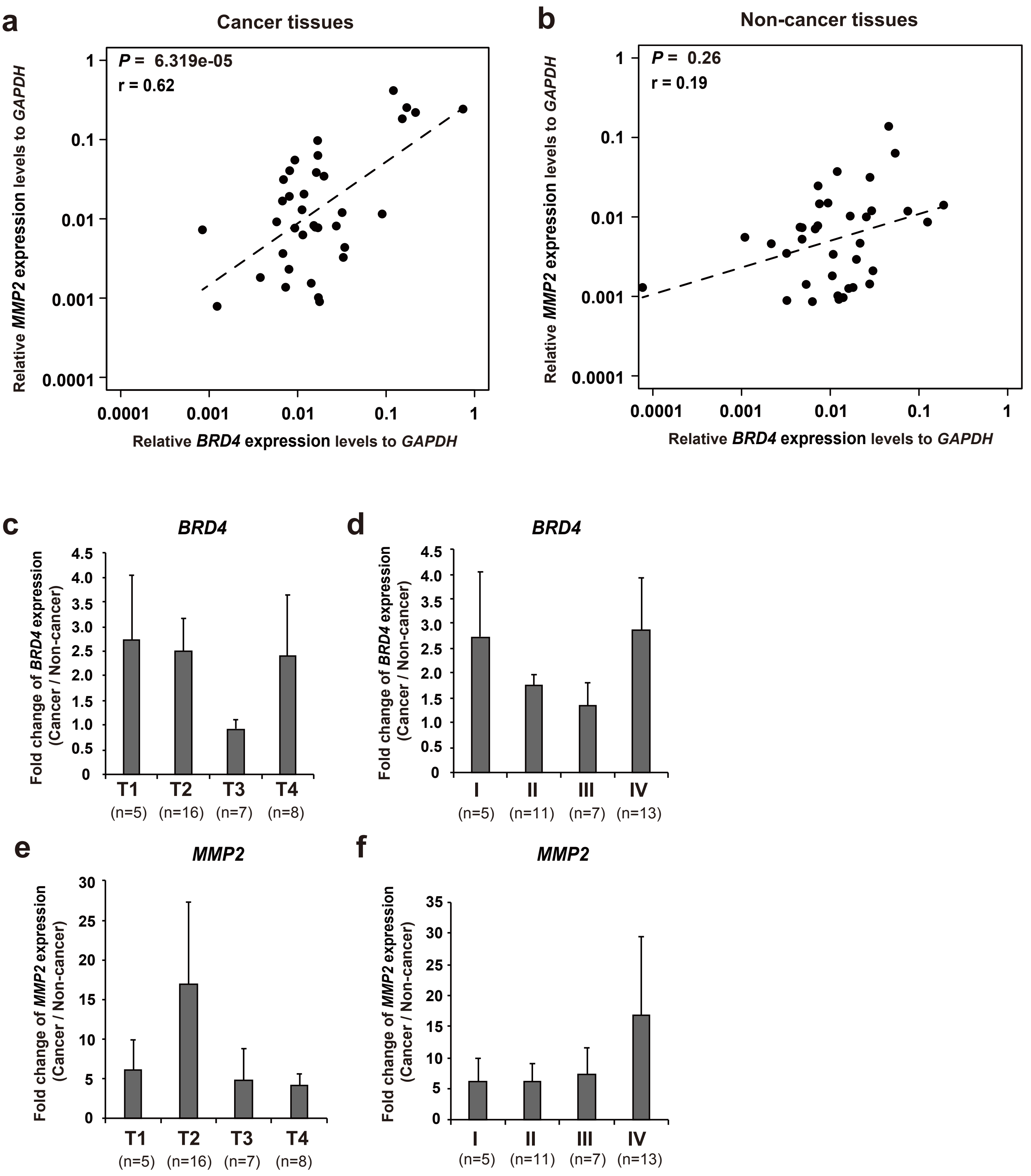

Yamamoto et al. Supplementary Figure 5

Supplementary Table 1. qRT-PCR Primers

genes	sequence
PTGS2-F	5'-GCAAATTGCTGGCAGGGTTG-3'
PTGS2-R	5'-GCTCTGGTCAATGGAAGCCT-3'
NRP1-F	5'-GAAGTGGAAGCCCCTACAGC-3'
NRP1-R	5'-CCACCTGTGAGCTGGAAGTC-3'
AURKA-F	5'-TACAGCTAGAGGCATCATGGAC-3'
AURKA-R	5'-ACGAGAACACGTTTTGGACCT-3'
VEGFC-F	5'-CTTCCTGCCGATGCATGTCT-3'
VEGFC-R	5'-CTGCCTGACACTGTGGTAGT-3'
IFIT2-F	5'-GCACTGCAACCATGAGTGAGA-3'
IFIT2-R	5'-CAAGTTCCAGGTGAAATGGCA-3'
CAV1-F	5'-CGTAGACTCGGAGGGACATCT-3'
CAV1-R	5'-CTTCTCGCTCAGCTCGTCTG-3'
S100A2-F	5'-TTAAAATGCCAAGTTGGGGGC-3'
S100A2-R	5'-ATCATGGATCTGTGGCAGAC-3'
INHBA-F	5'-ATGAAGAGGAGCTCAGACAGC-3'
INHBA-R	5'-CAAATTCTCTTTCTGGTCCCCA-3'
MKI67-F	5'-CGTCCCAGTGGAAGAGTTGT-3'
MKI67-R	5'-TCTCGTGGGCCACATTTTCT-3'
EMP1-F	5'-ATGCCAGTGAAGATGCCCTC-3'
EMP1-R	5'-TCCATGGTGAAGAGCTGGAAC-3'
CXCL8-F	5'-GAAGTTTTTGAAGAGGGCTGAGA-3'
CXCL8-R	5'-TTTGCTTGAAGTTTCACTGGCA-3'
F2RL1-F	5'-AGTGGCACCATCCAAGGAAC-3'
F2RL1-R	5'-TCCTTTTCCAGTGACGTGGG-3'
MMP2-F	5'-TACAGGATCATTGGCTACACACC-3'
MMP2-R	5'-GGTCACATCGCTCCAGACT-3'
SOD2-F	5'-GTGGAGAACCCAAAGGGGAG-3'
SOD2-R	5'-GAGCCTTGGACACCAACAGA-3'
TMEM97-F	5'-CCAGTCGAGTTTAGAAACCTGC-3'
TMEM97-R	5'-GCTGAAACACAAGCTCGCAA-3'
BRD4-F	5'-AGCAGCAACAGCAATGTGAG-3'
BRD4-R	5'-GCTTGCACTTGTCCTCTTCC-3'
36B4-F	5'-GATGCCCAGGGAAGACAG-3'
36B4-R	5'-TCTGCTCCCACAATGAAACAT-3'
GAPDH-F	5'-ACACCCACTCCTCCACCTTT-3'
GAPDH-R	5'-TAGCCAAATTCGTTGTCATACC-3'

Supplementary Table 2. ChIP-seq data used in this study

Cell lines	acccession number
PANC-1 H3K27ac	GSM818826
HT-1080 H3K27ac	GSM1663107
HeLa-S3 H3K27ac	GSM733684
HepG2 H3K27ac	GSM733743
MDA-MB-231 H3K2	GSM1204474
LNCaP H3K27ac	GSM1249448
MCF7 H3K27ac	GSM945854

Supplementary Table 3. ChIP-qPCR Primers

MMP2 locus	sequence
MMP2_a_F	5'-AAGATGGGAGGTTTCTCGGC-3'
MMP2_a_R	5'-TGCCTGGGTTGAGTTTCTGT-3'
MMP2_b_F	5'-TCGCCCATCATCAAGTTCCC-3'
MMP2_b_R	5'-CATCCTTTGCCTCCGAAGTT-3'
MMP2_c_F	5'-AGGGCACATCCTATGACAGC-3'
MMP2_c_R	5'-AAGCCATACTTCTTGTCGCG-3'
MMP2_d_F	5'-TCCAGGCAGAGTTATTTGCC-3'
MMP2_d_R	5'-CAGGGTGGTTCCTCTGTAGA-3'
**MYC locus**	**sequence**
MYC_promotor_R	5'-ACCGAAGTCCACTTGCCTTT-3'
MYC_promotor_F	5'-AGCGCAGGAATGGGAGAAAA-3'

Supplementary Table 4. Genes upregulated by JQ1

PrimaryAccession	GeneSymbol	HOC313_signal	HOC313JQ1_signal	compare1_Zscore	compare1_ratio	RefSeqAccession	GenbankAccession	EnsemblID
NR_109780	LOC100506860	643.76542	17493.955	6.1953547	27.174425	NR_109780		
NM_025249	KIAA1683	145.2944	6252.379	6.1944252	43.032484	NM_025249	NM_025249	ENST00000599638
NR_109780	LOC100506860	486.05645	13167.1	6.1894941	27.089652	NR_109780		
ENST00000607355		3836.996	72429.365	6.1360759	18.876581		L19779	ENST00000607355
NR_119376	FER1L4	1653.7065	27824.185	5.8950126	16.825347	NR_119376	NR_119376	ENST00000616711
NM_001040874	HIST2H2AA4	414.81415	8772.69	5.7251012	21.148483	NM_001040874	NM_001040874	ENST00000607355
NM_006472	TXNIP	1910.4895	29183.21	5.6924722	15.275253	NM_006472	NM_006472	ENST00000582401
NM_000146	FTL	51353.19	179614.2	5.4405019	3.497625	NM_000146	NM_000146	ENST00000622577
lnc-MKLN1-1:3	lnc-MKLN1-1	16.37579	830.9779	5.355184	50.744294			
ENST00000501122		3329.8655	42210.36	5.3016506	12.676296		AF001893	ENST00000501122
NM_006829	ADIRF	9459.0245	59692.55	5.2473018	6.3106455	NM_006829	NM_006829	ENST00000372013
NR_028272	NEAT1	16808.58	105816.2	5.2404302	6.2953682	NR_028272	NR_028272	ENST00000501122
NR_028272	NEAT1	744.5423	11225.702	5.0904079	15.077319	NR_028272	NR_028272	ENST00000499732
NM_014971	EFR3B	203.76445	4460.3235	5.0817986	21.889606	NM_014971	NM_014971	ENST00000403714
NM_000146	FTL	55500.893	177138.2	5.0395172	3.1916279	NM_000146	NM_000146	ENST00000427242
NR_028272	NEAT1	770.93165	11297.858	5.037095	14.654811	NR_028272	NR_028272	ENST00000499732
ENST00000314088	HIST1H2AC	1237.8407	15489.455	4.7407761	12.513287		BC050602	ENST00000314088
NM_003528	HIST2H2BE	19.134445	606.0207	4.7138537	31.671716	NM_003528	NM_003528	ENST00000369155
BC010926	HIST1H4H	40.834475	1279.623	4.6993911	31.33683		BC010926	
NR_034120	LINC-PINT	136.8192	2150.1073	4.5363037	15.714953	NR_034120	NR_034120	ENST00000447307
NR_119376	FER1L4	6.2305855	311.47805	4.5047399	49.991778	NR_119376	NR_119376	ENST00000325076
NR_028272	NEAT1	6729.271	56118.7	4.4241758	8.3394918	NR_028272	NR_028272	ENST00000616315
NR_002819	MALAT1	1938.052	16149.205	4.4224684	8.3326995	NR_002819	NR_002819	ENST00000618227
NR_109780	LOC100506860	112.26745	1591.021	4.3661607	14.171704	NR_109780	NR_109780	
ENST00000501122		134.44765	1883.1765	4.3468906	14.006764			ENST00000501122
ENST00000447430		26.554585	562.6106	4.1668591	21.186948			ENST00000447430
NR_033380	CD99P1	3.6305795	130.17945	4.125128	35.856383	NR_033380	NR_033380	ENST00000435581
NM_021065	HIST1H2AD	3625.781	25568.685	4.0727437	7.051911	NM_021065	NM_021065	ENST00000341023
NR_002819	MALAT1	2264.4855	15811.3	4.051953	6.9822924	NR_002819	NR_002819	ENST00000534336
NM_199461	NANOS1	46.18192	810.2071	3.910145	17.543816	NM_199461	NM_199461	ENST00000340087
NR_027232	LINC00685	167.86375	1777.118	3.8860917	10.586669	NR_027232	NR_027232	
NR_002819	MALAT1	656.1951	5044.041	3.8267726	7.6868008	NR_002819	NR_002819	ENST00000616691
ENST00000621399	CIRBP	742.55785	5666.3125	3.8130582	7.6308028		AK128423	ENST00000621399
ENST00000369167	HIST2H2BF	15.83156	431.95415	3.8130566	27.284371		BC110793	ENST00000369167
lnc-HIST2H2AA3-1:1	lnc-HIST2H2AA3-1	245.64945	2456.9995	3.7925888	10.002056		BX648801	
ENST00000447430		13.09223	348.71205	3.7855439	26.635038			ENST00000447430
ENST00000309775		21.95115	342.134	3.7491665	15.586154	XM_005274417	XM_005274417	ENST00000309775
ENST00000509399		36.357495	565.92385	3.7473654	15.565535			ENST00000509399
ENST00000423967		4.246365	106.10401	3.7125871	24.987021			ENST00000423967
NM_020376	PNPLA2	1487.941	10251.764	3.6214806	6.8898995	NM_020376	NM_020376	ENST00000529255
NM_001831	CLU	347.71915	3123.1505	3.6154948	8.9818191	NM_001831	NM_001831	ENST00000405140
ENST00000447430		30.34134	426.52145	3.6087148	14.057436			ENST00000447430
NM_001163278	TENM1	190.2412	1632.7815	3.5406744	8.5826913	NM_001163278	NM_001163278	ENST00000422452
ENST00000495363		275.9003	2305.9265	3.4969734	8.3578253			ENST00000495363
ENST00000432045		36.896425	461.65615	3.4502842	12.512219			ENST00000432045
ENST00000619960		726.4732	4564.4325	3.4485247	6.2830019	XM_006718754	XM_006718754	ENST00000619960
ENST00000509548		601.1808	3741.378	3.4306412	6.2233824			ENST00000509548
NM_001013642	TRNP1	780.40925	4805.336	3.4106654	6.1574565	NM_001013642	NM_001013642	ENST00000531285
NR_002918	SNORA48	1436.6175	8704.178	3.380369	6.0587999	NR_002918	NR_002918	ENST00000579085
NM_206922	CRIP3	8.0085635	149.59025	3.3802289	18.678787	NM_206922	NM_206922	ENST00000274990
NM_000694	ALDH3B1	663.68135	3984.7875	3.3633477	6.0040673	NM_000694	NM_000694	ENST00000615463
NM_152718	VWCE	150.67185	1159.4156	3.3609594	7.6949712	NM_152718	NM_152718	ENST00000335613
NR_047500	LINC00571	7.846579	143.9597	3.3597452	18.346811	NR_047500		ENST00000434565
ENST00000504402	FLJ36777	9.8296425	167.09475	3.2725946	16.999067			ENST00000504402
NM_007030	TPPP	8.4062725	142.1727	3.2667759	16.912692	NM_007030	NM_007030	ENST00000360578
NM_001554	CYR61	24561.955	76937.215	3.2615083	3.1323734	NM_001554	NM_001554	ENST00000617517
NM_003407	ZFP36	1449.3115	8184.771	3.2484601	5.6473512	NM_003407	NM_003407	ENST00000597629
NM_005090	JMJD7-PLA2G4B	649.3706	3598.6915	3.2130757	5.5418146	NM_005090	NM_005090	ENST00000483748
ENST00000359193	HIST1H2AG	38.524245	401.58665	3.201888	10.424258		L19778	ENST00000359193
NM_001098784	FAM89B	3410.0545	15806.685	3.1934616	4.6353174	NM_001098784	NM_001098784	ENST00000449319
NM_201380	PLEC	5181.0175	23850.98	3.1790424	4.603532	NM_201380	NM_201380	ENST00000322810
NR_002728	KCNQ1OT1	258.96755	1778.298	3.1735455	6.8668758	NR_002728		
NM_005167	PPM1J	49.69635	506.95115	3.1724288	10.200974	NM_005167	NM_005167	ENST00000471106
NM_001008394	EID3	158.0924	1082.8689	3.1693979	6.8495949	NM_001008394	NM_001008394	ENST00000527879
		171.293	1171.8123	3.1673268	6.8409818			
NM_182597	LSMEM1	153.5309	1040.4436	3.1518036	6.77677	NM_182597	NM_182597	ENST00000312849
NM_182847	ASIC4	699.39095	3749.6735	3.1509753	5.3613411	NM_182847	NM_182847	ENST00000347842
NM_001135219	PIP5KL1	208.8128	1402.5305	3.1371451	6.7166883	NM_001135219	NM_001135219	ENST00000300432
NM_000903	NQO1	1985.115	10494.091	3.1245678	5.2863892	NM_000903	NM_000903	ENST00000320623
NR_027033	MIRLET7BHG	1495.1225	7783.695	3.0958463	5.2060584	NR_027033	NR_027033	ENST00000360737
NM_013230	CD24	1078.0599	5536.3665	3.0702475	5.1354909	NM_013230	NM_013230	ENST00000606017
NM_001167676	FAM229A	1003.9915	5103.654	3.0511113	5.083364	NM_001167676	NM_001167676	ENST00000416512
NM_173620	HEXDC	3288.692	14230.02	3.049202	4.3269543	NM_173620	NM_173620	ENST00000337014
NR_120595	LINC01315	661.18785	3346.9585	3.0432259	5.0620387	NR_120595	NR_120595	ENST00000424852
NR_034089	LOC100131564	147.72995	919.06405	3.011017	6.2212439	NR_034089	NR_034089	ENST00000440778
NM_001282533	LZTS3	204.1897	1268.5069	3.0086739	6.2123942	NM_001282533	NM_001282533	ENST00000337576
NM_001076680	LYRM9	528.99265	3262.3045	2.9966055	6.1670129	NM_001076680	NM_001076680	ENST00000379103
NM_005104	BRD2	14951.11	42527.225	2.9881198	2.8444192	NM_005104	NM_005104	ENST00000553146
ENST00000379253	SAT1	113.07039	995.8265	2.9725357	8.8071381		AK310078	ENST00000379253
NR_027051	THAP7-AS1	8.467046	109.63548	2.961698	12.948492	NR_027051	NR_027051	
NM_017514	PLXNA3	3523.677	14517.825	2.9465371	4.1200783	NM_017514	NM_017514	ENST00000369682
NR_033889	LINC00544	6.500403	82.974855	2.9453571	12.764571	NR_033889		ENST00000400540
NM_006086	TUBB3	16718.28	46793.58	2.9424313	2.798947	NM_006086	NM_006086	ENST00000315491
NM_021063	HIST1H2BD	1408.7865	6724.526	2.9330537	4.7732754	NM_021063	NM_021063	ENST00000377777
NM_175065	HIST2H2AB	3265.259	13304.98	2.9233333	4.0747089	NM_175065	NM_175065	ENST00000331128
NR_034172	KANSL1-AS1	181.0491	1065.9058	2.9202252	5.8873852	NR_034172	NR_034172	
NM_002970	SAT1	5212.9965	21088.785	2.9082186	4.0454247	NM_002970	NM_002970	ENST00000474223
AK128004	LOC643551	40.241855	334.5506	2.8940563	8.3134985		AK128004	
ENST00000478375	TFAP2A	53.69355	443.39815	2.8849334	8.2579407		M61156	ENST00000478375
AJ312026		43.621695	358.59455	2.8787599	8.2205552		AJ312026	
NM_001001824	OR2T27	10.934531	131.4761	2.8770802	12.023936	NM_001001824	NM_001001824	ENST00000344889
NM_001034077	HIST2H4B	2266.1855	10437.686	2.8660763	4.605839	NM_001034077	NM_001034077	ENST00000578186
lnc-AF131215.3.1-1:1	lnc-AF131215.3.1-1	70.962195	576.1812	2.8619397	8.1195515		AJ312027	

NR_002819	MALAT1	844.6767	3877.174	2.8596671	4.5901278	NR_002819	NR_002819	ENST00000534336
NM_020317	RSRP1	963.1752	4390.7675	2.846755	4.5586384	NM_020317	NM_020317	ENST00000568212
NM_001161473	ALDH3B1	239.9582	1349.37	2.8446991	5.6233544	NM_001161473	NM_001161473	ENST00000619675
NM_004417	DUSP1	1602.935	7276.6608	2.8388991	4.5395856	NM_004417	NM_004417	ENST00000239223
NM_144650	ADHFE1	47.228465	374.8863	2.8311246	7.9377193	NM_144650	NM_144650	ENST00000496501
NM_000146	FTL	41507.855	111696.4	2.8308967	2.6909702	NM_000146	NM_000146	ENST00000455449
NR_034089	LOC100131564	145.4685	808.5117	2.8254524	5.5579847	NR_034089	NR_034089	ENST00000421202
NM_019112	ABCA7	381.9533	2102.64	2.8096752	5.5049662	NM_019112	NM_019112	ENST00000263094
NM_145754	KIFC2	487.87125	2625.059	2.772074	5.3806388	NM_145754	NM_145754	ENST00000301332
NM_021052	HIST1H2AE	1433.0985	6270.166	2.7697386	4.3752512	NM_021052	NM_021052	
lnc-SPIRE2-1:1	lnc-SPIRE2-1	148.59815	793.30235	2.7591553	5.3385749			
NM_004794	RAB33A	42.252255	316.5477	2.7524714	7.4918534	NM_004794	NM_004794	ENST00000257017
NM_138466	ZNF837	225.2473	1196.9355	2.7515212	5.3138726	NM_138466	NM_138466	ENST00000597582
NR_002819	MALAT1	411.81715	2181.1185	2.7460774	5.2963275	NR_002819	NR_002819	ENST00000534336
NM_005763	AASS	276.58055	1458.1275	2.7384935	5.2719813	NM_005763	NM_005763	ENST00000358954
NM_001032280	TFAP2A	584.3077	3070.6065	2.7332202	5.255119	NM_001032280	NM_001032280	ENST00000379604
NM_017565	FAM20A	7.8961535	83.499055	2.7303698	10.574649	NM_017565	NM_017565	ENST00000590074
NR_038386	LINC01125	8.462783	89.4506	2.7298545	10.56988	NR_038386	NR_038386	
ENST00000624918		801.85605	3433.4625	2.7292825	4.2818939			ENST00000624918
NM_024705	DHRS12	192.66425	1007.8685	2.7257164	5.231217	NM_024705	NM_024705	ENST00000218981
ENST00000374439	MUSK	216.00965	1125.2399	2.7187775	5.209211			ENST00000374439
NM_003540	HIST1H4F	874.55585	3723.4215	2.7185661	4.2575	NM_003540	NM_003540	ENST00000244537
NR_040058	RAD51-AS1	231.05135	1201.7902	2.716307	5.2013985	NR_040058		
NR_104172	LOC283352	471.26015	2449.355	2.7150596	5.1974583	NR_104172	NR_104172	
NM_016201	AMOTL2	6746.5445	24845.725	2.7113804	3.6827334	NM_016201	NM_016201	ENST00000422605
NM_138393	REEP6	1950.7285	8256.3215	2.7074885	4.2324298	NM_138393	NM_138393	ENST00000233596
NM_032872	SYTL1	192.82485	997.37185	2.7071121	5.1724238	NM_032872	NM_032872	ENST00000490170
NM_025008	ADAMTSL4	182.2906	939.5268	2.7012404	5.1540057	NM_025008	NM_025008	ENST00000369041
NM_015526	CLIP3	21.513895	219.60435	2.6900129	10.207559	NM_015526	NM_015526	ENST00000360535
NM_003512	HIST1H2AC	805.02895	3363.2365	2.6831129	4.1777833	NM_003512	NM_003512	ENST00000602637
NM_014818	TRIM66	189.16	961.04255	2.6776218	5.0805802	NM_014818	NM_014818	ENST00000299550
NR_002987	SNORA61	1152.208	4781.5215	2.6705418	4.1498772	NR_002987	NR_002987	
NM_003273	TM7SF2	187.40562	943.73417	2.663044	5.0357838	NM_003273	NM_003273	ENST00000527851
NR_015379	UCA1	1193.6437	4924.378	2.6594915	4.1255009	NR_015379	NR_015379	ENST00000589333
NR_002819	MALAT1	1243.2691	5126.8395	2.6586619	4.1236766	NR_002819	NR_002819	ENST00000618227
XM_006717313	FUBP3	3.4847905	112.39998	2.6468017	32.254443	XM_006717313	XM_006717313	
NM_001031716	NABP1	599.02515	2971.486	2.6382624	4.9605363	NM_001031716	NM_001031716	ENST00000409510
NM_002970	SAT1	9730.017	34398.1	2.625736	3.5352559	NM_002970	NM_002970	ENST00000474223
NM_020127	TUFT1	2072.9205	8396.9405	2.6252065	4.0507779	NM_020127	NM_020127	ENST00000353024
NR_027456	LINC00894	65.11053	437.7242	2.6051053	6.7227866	NR_027456		
ENST00000565449		3.6548	111.04278	2.6037519	30.382723			ENST00000565449
NM_016286	DCXR	4050.043	14156.955	2.6020412	3.4955072	NM_016286	NM_016286	ENST00000578273
ENST00000507324		25381.745	62932.63	2.5988001	2.4794446			ENST00000507324
NR_002999	SCARNA20	239.3519	1156.6899	2.5952498	4.8325913	NR_002999	NR_002999	
NR_034172	KANSL1-AS1	214.85065	1037.4582	2.5939379	4.828741	NR_034172	NR_034172	
NR_040109	PCAT19	7.2956495	68.427935	2.5933493	9.3792794	NR_040109	NR_040109	ENST00000598215
NR_027033	MIRLET7BHG	49.7974	327.54135	2.5753756	6.5774789	NR_027033	NR_027033	ENST00000360737
NR_014636	RALGPS1	21.72474	200.23215	2.573386	9.21678	NM_014636	NM_014636	ENST00000259351
NM_005984	SLC25A1	3129.0805	10783.71	2.5723241	3.4462872	NM_005984	NM_005984	ENST00000470922
THC2576171		55.94227	363.77395	2.5598121	6.5026669			
ENST00000361624	COX1	18255.925	44583.92	2.5558464	2.4421617		HV963900	ENST00000361624
NR_003285	RNA5-8S5	116079.7	209642.55	2.5456368	1.8060225	NR_003285	NR_003285	
NM_003527	HIST1H2BO	3308.9846	11250.401	2.5439605	3.3999556	NM_003527	NM_003527	
NM_006848	CCDC85B	5027.877	17059.05	2.5396031	3.3928933	NM_006848	NM_006848	ENST00000312579
ENST00000272233	RHOB	349.66295	1633.0895	2.5390812	4.6704677		AK309991	ENST00000272233
NR_027033	MIRLET7BHG	237.3276	1108.3163	2.5389109	4.6699847	NR_027033	NR_027033	ENST00000360737
NM_033196	ZNF682	11.69581	104.48406	2.5377228	8.9334604	NM_033196	NM_033196	ENST00000601100
NR_038847	LINC00672	7.61836	67.814995	2.5336317	8.9015215	NR_038847		
BX647358	PDXDC2P	354.202	1646.103	2.5309158	4.6473566		BX647358	
NM_003717	NPFF	270.0462	1252.2244	2.52727	4.6370747	NM_003717	NM_003717	ENST00000267017
NM_001031716	NABP1	2990.607	10083.384	2.5264629	3.3716847	NM_001031716	NM_001031716	ENST00000307834
NM_003519	HIST1H2BL	5912.7475	19931.69	2.5260183	3.3709692	NM_003519	NM_003519	ENST00000377401
NR_108047	RNF139-AS1	20.33117	177.3658	2.5106004	8.7238364	NR_108047	NR_108047	ENST00000499418
NM_001130088	ABLIM2	9.2916885	80.9909	2.5096379	8.7164889	NM_001130088	NM_001130088	ENST00000428004
NR_024248	TSSC2	260.35155	1193.6437	2.5085863	4.584738	NR_024248	NR_024248	ENST00000526488
NM_002046	GAPDH	98868.365	176444.47	2.4934733	1.7846403	NM_002046	NM_002046	ENST00000474249
XM_006710250	SIRPB1	215.5332	978.90855	2.4930976	4.5417994	XM_006710250	XM_006710250	
NM_001031735	IZUMO4	74.47156	460.5417	2.4914766	6.1841285	NM_001031735	NM_001031735	ENST00000481489
ENST00000565617		176.4192	795.1164	2.4804269	4.506972		BX649145	ENST00000565617
NR_103812	INTS6-AS1	224.3947	1008.0729	2.4750998	4.4924096	NR_103812	NR_103812	
ENST00000418546		70.64282	430.25675	2.4707412	6.0905942			ENST00000418546
NM_012268	PLD3	722.54875	2692.6195	2.4687279	3.7265576	NM_012268	NM_012268	ENST00000488311
AI421806		3303.541	10733.522	2.4488525	3.2490959		AI421806	
NM_016175	C5orf45	1066.912	3924.664	2.4443951	3.6785264	NM_016175	NM_016175	ENST00000610475
lnc-GOLPH3L-1:1	lnc-GOLPH3L-1	37.317595	221.43045	2.435228	5.9336742			
NM_002975	CLEC11A	1533.1045	5606.1195	2.433238	3.6567106	NM_002975	NM_002975	ENST00000250340
ENST00000450472		38374.39	89576.63	2.4277611	2.3342815			ENST00000450472
NM_001165967	HES7	37.05006	217.96045	2.4235273	5.8828636	NM_001165967	NM_001165967	ENST00000541682
NM_000034	ALDOA	43558.895	101521.25	2.4233669	2.3306663	NM_000034	NM_000034	ENST00000569545
NM_005319	HIST1H1C	8265.755	26512.965	2.4218953	3.2075672	NM_005319	NM_005319	ENST00000343677
NR_002768	HYMAI	36.34971	211.7509	2.4101676	5.8253807	NR_002768	NR_002768	
ENST00000513369		23855.14	55315.06	2.4088835	2.31879			ENST00000513369
NM_025008	ADAMTSL4	213.7402	921.23335	2.4068929	4.3100612	NM_025008	NM_025008	ENST00000369041
XR_244439	LOC101927372	56.27956	325.34515	2.3997334	5.7808759	XR_244439	XR_244439	
		3.5437165	81.04613	2.3992154	22.870376			
NM_030649	ACAP3	2126.707	7635.028	2.3987398	3.5900705	NM_030649	NM_030649	ENST00000467278
NM_001164389	RAPGEF6	3.8084005	86.983675	2.3982568	22.839949	NM_001164389	NM_001164389	
NM_018440	PAG1	66.05771	381.0813	2.3969154	5.7689148	NM_018440	NM_018440	ENST00000220597
NR_027409	GOLGA8A	337.9695	1447.463	2.3964568	4.2828214	NR_027409	NR_027409	ENST00000484716
NR_026795	LINC00202-1	24.299875	191.56965	2.3949174	7.8835847	NR_026795	NR_026795	ENST00000431296
		14.217405	111.89646	2.3930033	7.8703853			
NM_020376	PNPLA2	3896.2025	12310.487	2.3903286	3.1596117	NM_020376	NM_020376	ENST00000617551
AI421806		3392.1535	10712.538	2.3892824	3.1580346		AI421806	
NM_001039848	GPX4	34890.405	80294.855	2.3874739	2.3013449	NM_001039848	NM_001039848	ENST00000585480
NM_001098482	CRTC1	343.8146	1462.1375	2.384836	4.2526917	NM_001098482	NM_001098482	ENST00000338797
NM_000663	ABAT	94.70177	541.26805	2.3842594	5.7155009	NM_000663	NM_000663	ENST00000396600
NR_003610	PDXDC2P	305.14305	1293.3703	2.3793611	4.2385702	NR_003610	NR_003610	

NM_015490	SEC31B	323.76465	1371.5755	2.3784928	4.2363349	NM_015490	NM_015490	ENST00000462434
ENST00000463343	SF1	140.70545	799.0866	2.3755773	5.6791446		D26121	ENST00000463343
ENST00000361875	TSC22D2	884.0098	3130.8355	2.3732587	3.5416299			ENST00000361875
NM_001291860	HSPG2	22.0143	170.23855	2.3729014	7.7330894	NM_001291860	NM_001291860	ENST00000374676
AF307332	MGEA5	425.07455	1794.11	2.3724046	4.2206949		AF307332	
NM_000203	IDUA	171.534	723.5451	2.3713867	4.2180856	NM_000203	NM_000203	
AK128779		38.725025	219.028	2.3700166	5.6559809		AK128779	
lnc-C21orf58-1:1	lnc-C21orf58-1	255.90525	1077.874	2.3690118	4.2120042			
NM_004277	SLC25A27	124.77673	704.57945	2.3677875	5.6467215	NM_004277	NM_004277	ENST00000411689
AK074459	LOC340335	255.68595	1075.6144	2.3669687	4.2067794		AK074459	
NM_007215	POLG2	1519.9455	5359.025	2.3648566	3.5258008	NM_007215	NM_007215	ENST00000577506
NM_138345	VWA5B2	9.008878	69.039635	2.3625775	7.6635109	NM_138345	NM_138345	ENST00000426955
NM_001032392	PLGLB1	11.75915	90.083735	2.3621637	7.6607353	NM_001032392	NM_001032392	
NR_003003	SCARNA17	1071.4487	3767.537	2.3597965	3.516302	NR_003003	NR_003003	
NR_003942	SNORD76	168.38815	703.48395	2.3555715	4.1777521	NR_003942	NR_003942	
NM_001122607	RUNX1	135.00795	751.2186	2.3477709	5.5642545	NM_001122607	NM_001122607	ENST00000479325
NM_003127	SPTAN1	2192.458	7640.563	2.3429867	3.4849302	NM_003127	NM_003127	ENST00000372739
NM_003524	HIST1H2BH	4644.9645	14318.04	2.338542	3.0824864	NM_003524	NM_003524	
NM_003525	HIST1H2BI	2535.417	8804.3345	2.3363056	3.472539	NM_003525	NM_003525	ENST00000377733
lnc-RP11-1105G2.3.1-3:1	lnc-RP11-1105G2.3.1-3	107.47151	592.1623	2.3344266	5.509947		DA880232	
lnc-LTBP3-1:1	lnc-LTBP3-1	23.17001	172.77615	2.3313573	7.4568872		AK057616	
NM_000216	KAL1	38.00063	208.2553	2.3270891	5.4803118	NM_000216	NM_000216	ENST00000262648
NM_181077	GOLGA8A	168.87505	692.6571	2.3252888	4.1015952	NM_181077	NM_181077	ENST00000432566
NM_001024599	HIST2H2BF	3208.7245	9816.7635	2.3227865	3.0593974	NM_001024599	NM_001024599	ENST00000449108
NR_002819	MALAT1	3452.94	10524.523	2.3149574	3.0479888	NR_002819	NR_002819	ENST00000534336
NM_020995	HPR	21.55354	158.39819	2.3147192	7.3490567	NM_020995	NM_020995	ENST00000356967
ENST00000509530		11.397595	83.59918	2.3125025	7.3348088			ENST00000509530
NM_001286968	JUND	230.31065	937.02325	2.311961	4.068519	NM_001286968	NM_001286968	ENST00000252818
ENST00000427048		3.5864435	72.4102	2.3094481	20.189974			ENST00000427048
ENST00000513755	SCAMP1-AS1	9.123112	66.50474	2.3054559	7.289699			ENST00000513755
ENST00000395383	PHF20L1	219.2339	887.771	2.3042173	4.0494239		AB101203	ENST00000395383
ENST00000460066	OFCC1	4.0083675	80.328385	2.3040853	20.040175	XM_003118558	XM_003118558	ENST00000460066
NM_138463	TLCD1	506.81055	2051.6779	2.3037257	4.0482147	NM_138463	NM_138463	ENST00000394933
ENST00000427048		11.156525	81.174985	2.3033088	7.2760098	XR_241865		ENST00000427048
ENST00000486199	PHF20L1	178.92565	723.0085	2.3007211	4.040832	XM_005250942	XM_005250942	ENST00000486199
NM_021727	FADS3	967.49135	3288.692	2.2962644	3.3991952	NM_021727	NM_021727	ENST00000278829
BX647090	SMCR6	526.34225	2119.3255	2.2948791	4.026516		BX647090	
		3.6396185	71.165595	2.2863644	19.553037			
		14.480585	103.60003	2.2840576	7.1544092			
NM_006014	LAGE3	8990.1985	26992.905	2.2834343	3.0024815	NM_006014	NM_006014	ENST00000621786
ENST00000367203	SOX13	46.689335	247.59885	2.2823707	5.3031137		BC040649	ENST00000367203
NM_001198844	RBM4	39.1881	207.26565	2.2787435	5.2889946	NM_001198844	NM_001198844	
ENST00000449345		3.189132	61.636225	2.2779897	19.326959			ENST00000449345
NR_040058	RAD51-AS1	90.231905	476.65735	2.2770927	5.282581	NR_040058		
NR_040023	ERVK13-1	19.9993	141.75385	2.2733958	7.0879406	NR_040023	NR_040023	
NM_013313	YPEL1	18.75568	132.23925	2.2673663	7.0506241	NM_013313	NM_013313	ENST00000477675
NR_103536	FAM87B	3.892485	74.062975	2.266732	19.02717	NR_103536	NR_103536	ENST00000326734
BG533310		74.722495	391.63845	2.266403	5.2412389		BG533310	
XR_433669	LOC102725127	434.44085	1717.668	2.264858	3.9537442	XR_433669		
ENST00000361567	ND5	4269.5255	12702.34	2.2642483	2.9751175		HV444971	ENST00000361567
ENST00000272233	RHOB	544.1739	2147.896	2.2620797	3.9470765		AK124398	ENST00000272233
NR_033874	FLJ20021	1100.2841	3658.7965	2.2550499	3.3253199	NR_033874	NR_033874	
NR_110473	LINC-PINT	95.506565	495.9901	2.2538901	5.1932566	NR_110473	NR_110473	ENST00000418546
NM_000425	L1CAM	1742.0335	5757.2645	2.2435021	3.3049103	NM_000425	NM_000425	ENST00000361699
NM_014464	TINAG	4.0073635	73.812535	2.2433477	18.419226	NM_014464	NM_014464	ENST00000259782
NR_110157	LOC101927914	4.111268	75.298685	2.239269	18.315197	NR_110157		ENST00000444158
NR_024490	GABPB1-AS1	78.441065	401.92255	2.2355918	5.1238793	NR_024490	NR_024490	
NR_002569	SCARNA9	1615.0867	5303.7485	2.2315273	3.2838786	NR_002569	NR_002569	
ENST00000443426	ABCC10	48.750305	248.37435	2.2278554	5.0948266			ENST00000443426
NM_014818	TRIM66	122.17276	622.3978	2.2277434	5.0944073	NM_014818	NM_014818	ENST00000299550
NM_002507	NGFR	12.228395	83.090875	2.2251693	6.7949126	NM_002507	NM_002507	ENST00000172229
AK094623		551.973	2129.8405	2.2247609	3.8585954		AK094623	
NR_024507	LINC00598	3.379275	60.453605	2.2223346	17.889519	NR_024507	NR_024507	
NR_039981	LINC01004	118.22013	597.42005	2.2167621	5.0534545	NR_039981	NR_039981	
XR_424668	CRTC3-AS1	18.06911	121.56005	2.2137815	6.7275062	XR_424668	XR_424668	
ENST00000604257		15928.665	34472.095	2.2132215	2.1641547			ENST00000604257
NM_006200	PCSK5	8.352363	56.133825	2.2126273	6.7207119	NM_006200	NM_006200	ENST00000376752
BC041650		55.10625	277.43815	2.2116774	5.0346041		BC041650	
NM_033305	VPS13A	778.98025	2530.1555	2.2109418	3.2480355	NM_033305	NM_033305	ENST00000376636
NM_002428	MMP15	375.1281	1434.424	2.2098607	3.8238244	NM_002428	NM_002428	ENST00000219271
NM_003495	HIST1H4I	1613.275	5236.064	2.2095415	3.2456116	NM_003495	NM_003495	ENST00000615353
ENST00000493650	PCID2	354.03705	1350.99	2.2064704	3.8159566		BC008975	ENST00000493650
NM_031917	ANGPTL6	26.08083	174.26085	2.205955	6.6815684	NM_031917	NM_031917	ENST00000253109
NM_177401	MIDN	11381.634	32931.795	2.2058957	2.8934155	NM_177401	NM_177401	ENST00000300952
ENST00000624585		482.37725	1839.281	2.2051736	3.8129514	XR_432952	XR_432952	ENST00000624585
ENST00000608155	LOC100507520	41.3019	206.5069	2.2022766	4.999937	XR_109439	XR_109439	ENST00000608155
		4.558434	79.22857	2.201554	17.380655			
ENST00000485770		95.69054	478.12345	2.2013572	4.9965592			ENST00000485770
NR_037184	FLJ21408	11.63444	77.37664	2.2006578	6.6506544	NR_037184		
ENST00000585065		1226.6646	3961.7595	2.2003237	3.2297008	XR_429999	XR_429999	ENST00000585065
ENST00000585065		1414.8985	4566.688	2.1990875	3.2275729	XR_429999	XR_429999	ENST00000585065
NM_016270	KLF2	549.94624	2080.4697	2.1922109	3.783042	NM_016270	NM_016270	ENST00000248071
AF461897		27.645065	182.42745	2.191737	6.5989156		AF461897	
NM_000854	GSTT2	1139.4885	3657.2225	2.1885725	3.20953	NM_000854	NM_000854	ENST00000616938
AK123605	PRDM2	20.86586	137.11175	2.1869128	6.5711047		AK123605	
XM_006717655	C10orf128	4.238624	72.09542	2.1859951	17.009157	XM_006717655	XM_006717655	ENST00000374148
NM_004277	SLC25A27	45.441285	224.0461	2.1832363	4.9304526	NM_004277	NM_004277	ENST00000411689
ENST00000532805	CYP2R1	57.75702	284.45205	2.1817248	4.924978		AK092584	ENST00000532805
NR_073423	ADAM3A	4.251199	71.73793	2.1802823	16.874752	NR_073423	NR_073423	ENST00000474764
ENST00000419664		8.9734595	58.17079	2.1714126	6.4825378	XR_430375		ENST00000419664
NR_039981	LINC01004	122.35563	597.7756	2.1707911	4.8855586	NR_039981	NR_039981	
XR_424215	LOC102723539	55.78079	271.89315	2.1676564	4.8743152	XR_424215	XR_424215	
NM_016352	CPA4	3374.401	9580.5895	2.1662555	2.8391971	NM_016352	NM_016352	ENST00000222482
BC087859	LOC401317	11.231486	72.423255	2.1653522	6.4482345		BC087859	
		4932.77	13978.54	2.1622767	2.8338114			
NM_001901	CTGF	260.5238	967.5495	2.1618315	3.7138622	NM_001901	NM_001901	ENST00000367976
NR_004400	RNVU1-18	3552.876	10045.337	2.1575167	2.8273818	NR_004400	NR_004400	

NM_001164586	IGFN1	143.17115	692.4183	2.1570031	4.8362977	NM_001164586	NM_001164586	ENST00000437879
NM_003684	MKNK1	269.6909	996.4943	2.1534278	3.6949497	NM_003684	NM_003684	ENST00000371945
NR_125979	LOC101929516	41.24346	198.78905	2.1523802	4.8198927	NR_125979	NR_125979	
AK098015	DBNDD2	4.414621	71.64502	2.1521855	16.229031		AK098015	
NM_001008739	C6orf226	1463.258	4596.733	2.1483497	3.1414371	NM_001008739	NM_001008739	ENST00000408925
NM_133444	ZNF526	30823.065	65099.15	2.1440987	2.1120271	NM_133444	NM_133444	ENST00000301215
NM_031916	ROPN1L	97.677157	467.87332	2.1439151	4.7899973	NM_031916	NM_031916	ENST00000503804
ENST00000512278		9559.1475	26813.26	2.1408504	2.8049844			ENST00000512278
XR_248470	LOC101927757	3.3977385	54.116895	2.1386722	15.927328	XR_248470	XR_248470	
lnc-QSOX2-1:16	lnc-QSOX2-1	3.5725315	56.75077	2.13677	15.88531	XM_006717352	XM_006717352	
NR_003002	SCARNA13	135.8275	646.58495	2.1354648	4.760339	NR_003002	NR_003002	
AA378382	SNORA12	53.593345	254.9307	2.1344416	4.7567604		AA378382	
NM_021216	ZNF71	3.339632	52.79333	2.1332626	15.808128	NM_021216	NM_021216	ENST00000328070
lnc-C21orf58-1:1	lnc-C21orf58-1	98.167945	466.40785	2.1328278	4.7511217			
NM_016564	CEND1	659.94245	2406.847	2.1319525	3.6470559	NM_016564	NM_016564	ENST00000330106
NM_178457	ZNF831	3.68945	58.14578	2.1310675	15.760013	NM_178457	NM_178457	ENST00000371030
ENST00000380201	DDRGK1	312.38795	1137.4876	2.1293372	3.6412658			ENST00000380201
		11.4988	71.79715	2.1285668	6.243882			
NM_005952	MT1X	4083.2115	11381.634	2.1276886	2.787422	NM_005952	NM_005952	ENST00000568370
ENST00000434947		3.727366	58.40737	2.1269371	15.669878			ENST00000434947
NM_001291860	HSPG2	5623.1925	15663.035	2.126194	2.7854346	NM_001291860	NM_001291860	ENST00000374695
NR_040288	AKR7L	50.437905	238.2628	2.1250054	4.7238838	NR_040288	NR_040288	ENST00000457194
NR_038386	LINC01125	79.60494	375.76495	2.1239937	4.7203723	NR_038386		
lnc-AF131215.4.1-1:1	lnc-AF131215.4.1-1	4.5024825	70.155875	2.1228687	15.581599		BC069683	
NM_001143888	BSDC1	219.4048	795.6569	2.1226185	3.6264334	NM_001143888	NM_001143888	ENST00000341071
NM_004861	GAL3ST1	59.532727	280.16962	2.1198866	4.7061446	NM_004861	NM_004861	ENST00000406361
NM_017885	HCFC1R1	8845.6639	24561.955	2.1196294	2.7767226	NM_017885	NM_017885	ENST00000576921
NR_024127	SNHG12	3779.5425	10493.624	2.1194063	2.776427	NR_024127	NR_024127	
NM_152390	TMEM178A	4.6702355	72.36568	2.1188591	15.495082	NM_152390	NM_152390	ENST00000482239
NM_005345	HSPA1A	24165.678	50555.189	2.1171205	2.0920244	NM_005345	NM_005345	ENST00000375651
NM_080593	HIST1H2BK	1689.6725	5218.0965	2.1163082	3.0882295	NM_080593	NM_080593	ENST00000356950
NR_001591	TPTEP1	207.5822	748.8489	2.1139936	3.6074813	NR_001591	NR_001591	ENST00000381646
NM_020928	ZSWIM6	119.66087	555.7127	2.1018196	4.6440639	NM_020928	NM_020928	ENST00000252744
ENST00000571219	LOC283887	17.187265	104.7025	2.1004128	6.0918648	XR_132607	XR_132607	ENST00000571219
NM_006460	HEXIM1	1700.0555	5201.7735	2.0989406	3.0597669	NM_006460	NM_006460	ENST00000332499
NM_178339	C3orf35	11.83589	71.99378	2.0986869	6.0826672	NM_178339	NM_178339	ENST00000624754
NM_001142683	CCDC121	3.928097	58.756955	2.0934618	14.958122	NM_001142683	NM_001142683	ENST00000324364
lnc-INTS9-1:3	lnc-INTS9-1	9.998939	60.518295	2.0930025	6.0524717		BC043205	
		87.555815	403.76135	2.0922382	4.6114738			
HW531573		14.52172	87.628475	2.0895686	6.0343041		HW531573	
NM_170744	UNC5B	4.333386	64.456695	2.0894219	14.874441	NM_170744	NM_170744	ENST00000335350
BC020495	RABL2B	152.23515	699.7815	2.0878766	4.5967144		BC020495	
NM_014971	EFR3B	4.2695815	62.747155	2.0807467	14.696324	NM_014971	NM_014971	ENST00000403714
		1433.8475	4341.6135	2.0793319	3.0279465			
NM_006019	TCIRG1	3070.6065	9285.3935	2.0768613	3.0239607	NM_006019	NM_006019	ENST00000530802
NM_003492	TMEM187	971.5261	2935.277	2.0752135	3.0213053	NM_003492	NM_003492	ENST00000369982
NM_018440	PAG1	33.973085	202.3919	2.0749213	5.9574189	NM_018440	NM_018440	ENST00000523463
NM_030807	SLC2A11	33.793005	201.31515	2.074899	5.9573024	NM_030807	NM_030807	ENST00000461809
NM_145170	CFAP70	27.70342	164.89665	2.0739227	5.9522128	NM_145170	NM_145170	ENST00000394865
NM_007170	TESK2	48.31872	219.56035	2.0721844	4.5440018	NM_007170	NM_007170	ENST00000486676
ENST00000619210	DUXAP10	44.306165	201.3073	2.0720493	4.5435505		AB451460	ENST00000619210
ENST00000488687	PSME4	9.14967	54.29379	2.0704148	5.9339616		AK124923	ENST00000488687
NR_105017	LOC100505878	3.3920915	48.820465	2.0657001	14.392438	NR_105017	NR_105017	ENST00000503843
		55.60379	251.4287	2.0655177	4.5217907			
NM_198443	NRN1L	54.99952	248.45055	2.0641725	4.5173222	NM_198443	NM_198443	ENST00000572067
ENST00000503577		3.879466	55.655245	2.0633784	14.34611			ENST00000503577
NR_103765	CAND1.11	21.55564	127.0748	2.0629289	5.8951996	NR_103765	NR_103765	ENST00000527261
NM_020928	ZSWIM6	532.69155	1859.464	2.0598245	3.4906955	NM_020928	NM_020928	ENST00000252744
NR_024607	MIR503HG	572.12147	1991.896	2.0555282	3.4815963	NR_024607	NR_024607	ENST00000440570
NM_002204	ITGA3	10783.71	29012.69	2.053463	2.6904182	NM_002204	NM_002204	ENST00000505306
NR_024493	LINC00087	362.55235	1260.6591	2.0534379	3.477178	NR_024493	NR_024493	ENST00000417443
NR_002996	SNORA80A	696.8437	2422.0445	2.052755	3.4757357	NR_002996	NR_002996	
XR_109894	LOC100507460	23.76429	138.7846	2.0521925	5.8400482	XR_109894		
NM_198055	MZF1	1460.2765	4357.3335	2.0518526	2.9839099	NM_198055	NM_198055	
NM_000018	ACADVL	11338.873	30471.645	2.0510804	2.687361	NM_000018	NM_000018	ENST00000583858
NM_002292	LAMB2	6234.9215	16751.785	2.0506176	2.6867676	NM_002292	NM_002292	ENST00000305544
AF348999	MT1DP	10.252406	59.459785	2.0442524	5.7995933		AF348999	
lnc-USP8-2:8	lnc-USP8-2	112.71074	501.1579	2.0426447	4.4464077			
ENST00000482019		4.60074	64.0801	2.0420902	13.928216			ENST00000482019
NR_034172	KANSL1-AS1	57.10899	253.72585	2.0415512	4.4428355	NR_034172		
NM_002574	PRDX1	74278.57	119487.45	2.0387265	1.6086396	NM_002574	NM_002574	ENST00000372079
NR_121612	LINC00472	3.378547	46.79835	2.0381191	13.85162	NR_121612	NR_121612	ENST00000426635
NM_015989	CSAD	106.10401	468.8093	2.0340456	4.4183938	NM_015989	NM_015989	ENST00000444623
NM_001010870	TDRD6	26.77978	153.70695	2.0323878	5.7396644	NM_001010870	NM_001010870	ENST00000316081
NM_012232	PTRF	94119.48	151107.2	2.0301229	1.6054827	NM_012232	NM_012232	ENST00000357037
ENST00000416718	LOC101929612	32518.3	65964.825	2.0297629	2.0285447		AK057071	ENST00000416718
NM_020928	ZSWIM6	142.6052	627.9923	2.0295173	4.4037125	NM_020928	NM_020928	ENST00000252744
NM_001080836	MEIG1	48.511415	213.5423	2.0289566	4.401898	NM_001080836	NM_001080836	ENST00000378240
THC2506066		3924.664	10429.414	2.0275884	2.6574029			
AK128032	LOC100128184	53.894705	236.42875	2.0243019	4.3868642		AK128032	
NM_001135865	NPIPB5	8941.2475	23689.62	2.0213281	2.6494759	NM_001135865	NM_001135865	ENST00000451409
NM_001008739	C6orf226	458.47495	1562.5455	2.0204264	3.4081371	NM_001008739	NM_001008739	ENST00000408925
NM_138465	GLI4	648.6301	2207.882	2.0183862	3.4039154	NM_138465	NM_138465	ENST00000340042
AK095428	SAP30L-AS1	42.06697	183.1893	2.0142917	4.3547063		AK095428	
		3.8864225	51.99775	2.0131376	13.379335			
DB221055		52.92745	230.09195	2.0119784	4.3473084		DB221055	
NM_138448	ACYP2	568.7428	1926.7855	2.0105735	3.3877976	NM_138448	NM_138448	ENST00000394666
NM_032039	ITFG3	3933.7625	10353.374	2.0074016	2.6319266	NM_032039	NM_032039	ENST00000301678
ENST00000506686	LUC7L3	112.95008	489.1749	2.0068318	4.3308949		BC056409	ENST00000506686
ENST00000623879		5391.8575	14178.215	2.0055168	2.6295604		M15530	ENST00000623879
ENST00000561287		89.21606	385.9328	2.0052372	4.3258221		NR_024474	ENST00000561287
AK023835	FLJ13773	72429.365	115618.65	2.0049871	1.5962952		AK023835	
NM_014653	WSCD2	73.956685	319.415	2.0030732	4.318947	NM_014653	NM_014653	ENST00000547525

Supplementary Table 5. Genes downregulated by JQ1

PrimaryAccession	GeneSymbol	HOC313_signal	HOC313JQ1_signal	compare1_Zscore	compare1_ratio	RefSeqAccession	GenbankAccession	EnsemblID
NM_017789	SEMA4C	5961.347	2050.1028	-2.000864	0.3438993	NM_017789	NM_017789	ENST00000467747
NM_013431	KLRC4	65.71197	3.3349085	-2.001196	0.0507504	NM_013431	NM_013431	ENST00000309384
NM_030920	ANP32E	678.105	154.23545	-2.002165	0.2274507	NM_030920	NM_030920	ENST00000436748
NM_000204	CFI	130.17945	21.84889	-2.002227	0.1678367	NM_000204	NM_000204	ENST00000618244
NR_040001	LINC01116	2305.9265	682.20965	-2.002593	0.2958506	NR_040001	NR_040001	ENST00000295549
NM_033342	TRIM7	1014.2777	300.0451	-2.002755	0.2958215	NM_033342	NM_033342	ENST00000334421
NM_021101	CLDN1	115.99957	19.45537	-2.003026	0.1677193	NM_021101	NM_021101	ENST00000295522
NM_182487	OLFML2A	779.5751	230.53395	-2.003334	0.2957174	NM_182487	NM_182487	ENST00000373580
NM_001093729	CCDC102B	72.59844	3.668057	-2.004397	0.0505253	NM_001093729	NM_001093729	ENST00000358653
NR_026583	RACGAP1P	119.8516	20.054335	-2.005706	0.1673264	NR_026583	NR_026583	ENST00000550663
ENST00000511422		187.0039	31.28079	-2.006067	0.1672735			ENST00000511422
NM_004438	EPHA4	435.00566	98.540903	-2.007696	0.2265279	NM_004438	NM_004438	ENST00000424339
NM_002535	OAS2	202.6358	45.8101	-2.010442	0.2260711	NM_002535	NM_002535	ENST00000620097
NM_030762	BHLHE41	2196.2786	646.30435	-2.011397	0.2942725	NM_030762	NM_030762	ENST00000242728
NM_032744	ADTRP	318.14015	71.83223	-2.012147	0.225788	NM_032744	NM_032744	ENST00000514824
ENST00000521369		4075.0085	1392.62	-2.012642	0.3417465		BC040619	ENST00000521369
NM_033504	TMEM54	13125.6	5071.418	-2.013256	0.3863761	NM_033504	NM_033504	ENST00000373463
NR_125359	LOC79160	264.58775	59.596145	-2.015444	0.2252415	NR_125359	NR_125359	ENST00000598340
NM_004126	GNG11	9364.1311	3613.6144	-2.015842	0.3858996	NM_004126	NM_004126	ENST00000248564
NR_038955	LINC00963	155.0907	25.709815	-2.016361	0.1657728	NR_038955	NR_038955	ENST00000608369
NR_110184	LOC101927204	74.979635	3.718887	-2.017727	0.0495986	NR_110184	NR_110184	ENST00000501817
ENST00000378979	C20orf196	364.17715	81.781225	-2.01954	0.2245644	XM_006723549	XM_006723549	ENST00000378979
NM_052906	ELFN2	436.86555	98.04584	-2.020354	0.2244302	NM_052906	NM_052906	ENST00000613079
NM_012183	FOXD3	106.05702	17.512175	-2.020865	0.1651204	NM_012183	NM_012183	ENST00000371116
THC2642375		85.559635	14.12157	-2.021356	0.1650494			
NM_005627	SGK1	1643.662	480.54195	-2.022126	0.2923606	NM_005627	NM_005627	ENST00000367857
ENST00000326071	NOL4L	864.03975	252.37975	-2.023635	0.2920928		AK097804	ENST00000326071
NM_030932	DIAPH3	433.61305	97.07846	-2.023677	0.2238827	NM_030932	NM_030932	ENST00000400324
ENST00000333129	FIGN	208.4378	34.31573	-2.024242	0.164633			ENST00000333129
NM_001267595	SENP1	974.77025	284.5062	-2.024891	0.29187	NM_001267595	NM_001267595	ENST00000448372
NM_021219	JAM2	69.66384	11.460625	-2.025073	0.1645133	NM_021219	NM_021219	ENST00000312957
NM_017439	GSAP	170.9078	28.116125	-2.025092	0.1645105	NM_017439	NM_017439	ENST00000482866
NM_001005271	CHD3	2348.792	685.1013	-2.025949	0.2916824	NM_001005271	NM_001005271	ENST00000330494
NM_022159	ELTD1	89.813025	14.746675	-2.027298	0.1641931	NM_022159	NM_022159	ENST00000370742
NM_001459	FLT3LG	152.0869	24.96833	-2.027448	0.1641715	NM_001459	NM_001459	ENST00000593422
ENST00000441085		78.84583	12.94275	-2.027579	0.1641526			ENST00000441085
THC2538882		168.46955	27.649995	-2.027774	0.1641246			
ENST00000619168	CAMK1D	214.72291	47.91436	-2.028167	0.2231451	XM_006717483	XM_006717483	ENST00000619168
NM_000071	CBS	9837.096	3773.302	-2.028482	0.3835789	NM_000071	NM_000071	ENST00000617706
ENST00000444184	LINC00963	918.13415	267.3906	-2.028489	0.2912326			ENST00000444184
NM_003390	WEE1	830.9779	241.74515	-2.030277	0.2909165	NM_003390	NM_003390	ENST00000299613
NM_005192	CDKN3	1424.068	413.83855	-2.032051	0.2906031	NM_005192	NM_005192	ENST00000216414
ENST00000394243	SYNPO	330.56155	73.549685	-2.032111	0.2224992			ENST00000394243
NM_006216	SERPINE2	3377.191	1141.6095	-2.033124	0.3380352	NM_006216	NM_006216	ENST00000478966
NM_002213	ITGB5	2875.4435	971.7225	-2.033662	0.3379383	NM_002213	NM_002213	ENST00000460797
NM_001920	DCN	66.975655	3.2477965	-2.033974	0.0484922	NM_001920	NM_001920	ENST00000552962
NR_040001	LINC01116	1154.452	335.00495	-2.034419	0.2901853	NR_040001	NR_040001	ENST00000295549
NM_144991	TSPEAR	89.409495	4.32604	-2.035574	0.0483846	NM_144991	NM_144991	ENST00000614657
NR_033931	LINC01085	74.307335	3.5950495	-2.035629	0.0483808	NR_033931	NR_033931	ENST00000511200
NM_001024465	SOD2	17878.82	6834.326	-2.035709	0.3822582	NM_001024465	NM_001024465	ENST00000541573
NM_018715	RCC2	116.6339	18.962505	-2.038565	0.1625814	NM_018715	NM_018715	ENST00000375433
NM_020374	C12orf4	641.36235	185.59565	-2.039009	0.2893772	NM_020374	NM_020374	ENST00000545746
NM_001080400	PLIN4	362.15305	80.07604	-2.040626	0.221111	NM_001080400	NM_001080400	ENST00000301286
NR_120392	LINC00403	90.10933	4.3244675	-2.04145	0.0479913	NR_120392		
NM_002121	HLA-DPB1	219.56035	48.48222	-2.042448	0.220815	NM_002121	NM_002121	ENST00000402095
NM_001042550	SMC2	793.0769	228.5762	-2.045637	0.2882144	NM_001042550	NM_001042550	ENST00000286398
NR_024391	LINC00669	70.81656	3.3731975	-2.046849	0.0476329	NR_024391	NR_024391	ENST00000591469
ENST00000284110	HS3ST3A1	358.5344	78.819635	-2.048479	0.2198384		AF105376	ENST00000284110
NM_001194958	KCNJ18	99.8062	16.058125	-2.050489	0.1608931	NM_001194958	NM_001194958	ENST00000567955
NM_080655	MSANTD3	1621.325	465.40405	-2.052291	0.2870517	NM_080655	NM_080655	ENST00000583159
NR_033925	FENDRR	271.0729	59.412735	-2.052583	0.2191762	NR_033925	NR_033925	ENST00000595886
NM_015429	ABI3BP	69.131365	3.263682	-2.053273	0.0472099	NM_015429	NM_015429	ENST00000475896
XR_425103	LOC102723834	75.858857	3.58081	-2.053369	0.0472036	XR_425103	XR_425103	
NM_002727	SRGN	13751.595	5204.506	-2.056604	0.3784656	NM_002727	NM_002727	ENST00000462445
NM_001289	CLIC2	486.39295	106.20011	-2.057771	0.2183422	NM_001289	NM_001289	ENST00000369449
NR_038996	USP30-AS1	104.44675	16.69141	-2.05822	0.1598079	NR_038996	NR_038996	
NM_005375	MYB	76.50963	12.214485	-2.059374	0.1596464	NM_005375	NM_005375	ENST00000341911
ENST00000441360		120.87765	19.28246	-2.060276	0.1595205			ENST00000441360
NM_018071	ARHGEF40	4894.086	1630.572	-2.060305	0.3331719	NM_018071	NM_018071	ENST00000553709
NM_014573	TMEM97	2599.658	865.81035	-2.061004	0.3330478	NM_014573	NM_014573	ENST00000226230
AY358259		73.73283	3.442342	-2.061297	0.0466867		AY358259	
NM_173653	SLC9A9	172.68725	27.521065	-2.061358	0.1593694	NM_173653	NM_173653	ENST00000316549
NM_002053	GBP1	462.64995	100.65526	-2.062638	0.2175625	NM_002053	NM_002053	ENST00000370473
NM_005915	MCM6	3663.6685	1217.26	-2.065493	0.3322517	NM_005915	NM_005915	ENST00000264156
NM_024596	MCPH1	163.02295	25.869175	-2.066286	0.1586833	NM_024596	NM_024596	ENST00000344683
NM_005224	ARID3A	1303.256	370.8821	-2.066519	0.2845812	NM_005224	NM_005224	ENST00000263620
NM_003687	PDLIM4	273.69895	59.327205	-2.067661	0.2167608	NM_003687	NM_003687	ENST00000253754
ENST00000419300	LINC00963	185.74305	40.215485	-2.069227	0.2165114			ENST00000419300
NM_170589	CASC5	695.46751	197.49556	-2.070027	0.2839783	NM_170589	NM_170589	ENST00000527044
NM_153350	FBXL16	108.99199	17.233375	-2.070377	0.158116	NM_153350	NM_153350	ENST00000324361
NM_006215	SERPINA4	85.978355	3.9614785	-2.07079	0.0460753	NM_006215	NM_006215	ENST00000298841
NM_000494	COL17A1	75.74377	3.4899155	-2.07079	0.0460753	NM_000494	NM_000494	ENST00000433822
NR_125805	TMEM92-AS1	191.9505	30.302975	-2.072165	0.1578687	NR_125805		
NM_002214	ITGB8	595.26969	128.57695	-2.072458	0.2159978	NM_002214	NM_002214	ENST00000222573
ENST00000418372	LINC00702	74.408305	3.4199025	-2.072574	0.0459613			ENST00000418372
NM_000358	TGFBI	140300.85	88275.635	-2.072659	0.6291882	NM_000358	NM_000358	ENST00000504411
NM_024680	E2F8	85.53019	3.930444	-2.07269	0.0459539	NM_024680	NM_024680	ENST00000620009

NM_022550	XRCC4	381.13569	82.247896	-2.073724	0.2157969	NM_022550	NM_022550	ENST00000542685
NM_001278381	MPP2	642.9638	182.11395	-2.074287	0.2832414	NM_001278381	NM_001278381	ENST00000269095
ENST00000552466		75.598895	3.4564475	-2.076351	0.0457209			ENST00000552466
NM_001134707	SARDH	3692.3375	1219.3692	-2.076867	0.3302432	NM_001134707	NM_001134707	ENST00000439388
NM_004530	MMP2	618.0943	132.9512	-2.078134	0.2150986	NM_004530	NM_004530	ENST00000219070
NM_006401	ANP32B	7078.3895	2335.4785	-2.078561	0.3299449	NM_006401	NM_006401	ENST00000486769
NM_017631	DDX60	660.7195	186.5642	-2.079386	0.2823652	NM_017631	NM_017631	ENST00000393743
NM_030941	LOC81691	86.266435	13.51734	-2.080704	0.1566929	NM_030941	NM_030941	ENST00000564274
NM_017785	SPDL1	832.9569	234.9388	-2.081202	0.282054	NM_017785	NM_017785	ENST00000265295
NM_001288	CLIC1	31820.22	15140.04	-2.081262	0.4757994	NM_001288	NM_001288	ENST00000614673
NM_001042610	DBNDD1	66.17684	10.36435	-2.081265	0.156616	NM_001042610	NM_001042610	ENST00000623401
NM_003276	TMPO	185.59565	39.80764	-2.082015	0.2144858	NM_003276	NM_003276	ENST00000266732
NM_003598	TEAD2	65.27436	10.196057	-2.08428	0.1562031	NM_003598	NM_003598	ENST00000311227
NM_138554	TLR4	222.36055	47.61196	-2.084335	0.2141205	NM_138554	NM_138554	ENST00000472304
NM_005729	PPIF	14328.19	5351.2385	-2.084414	0.3734762	NM_005729	NM_005729	ENST00000394579
NM_152377	C1orf87	62.443595	9.7500965	-2.084724	0.1561425	NM_152377	NM_152377	ENST00000488027
NM_015184	PLCL2	192.7297	41.233395	-2.085456	0.2139442	NM_015184	NM_015184	ENST00000432376
NR_037889	LOC285626	78.75334	3.5542245	-2.085701	0.0451311	NR_037889	NR_037889	ENST00000515337
NR_030732	WFDC21P	5645.232	1855.538	-2.085702	0.3286912	NR_030732	NR_030732	ENST00000587298
NM_021822	APOBEC3G	521.3759	111.4675	-2.086405	0.2137949	NM_021822	NM_021822	ENST00000480000
ENST00000447056		87.628475	3.9343725	-2.089424	0.0448983			ENST00000447056
NM_138461	TM4SF19	464.84795	99.058445	-2.090844	0.2130986	NM_138461	NM_138461	ENST00000442633
NR_002734	PTTG3P	686.05595	192.23155	-2.092068	0.2801981	NR_002734	NR_002734	ENST00000521862
NM_002849	PTPRR	186.458	28.89143	-2.09349	0.1549487	NM_002849	NM_002849	ENST00000283228
NM_001011724	HNRNPA1L2	31798.14	11821.047	-2.094107	0.3717528	NM_001011724	NM_001011724	ENST00000357495
NM_172088	TNFSF13	72.15968	3.209663	-2.096165	0.04448	NM_172088	NM_172088	ENST00000396542
NM_001018112	FANCA	60.23547	9.3108985	-2.096248	0.154575	NM_001018112	NM_001018112	ENST00000566889
NM_001077693	ECSCR	85.28341	3.784111	-2.097932	0.044371	NM_001077693	NM_001077693	ENST00000618155
NM_018371	CSGALNACT1	244.66515	51.853965	-2.098271	0.2119385	NM_018371	NM_018371	ENST00000454498
NM_000043	FAS	298.0729	63.10441	-2.099751	0.211708	NM_000043	NM_000043	ENST00000612663
NM_153020	RBM24	99.48801	15.286105	-2.103121	0.1536477	NM_153020	NM_153020	ENST00000318204
NM_003113	SP100	30983.215	11444.84	-2.107477	0.3693884	NM_003113	NM_003113	ENST00000264052
NM_001200	BMP2	1387.5649	385.06145	-2.107943	0.2775088	NM_001200	NM_001200	ENST00000378827
ENST00000450890	LOC101929475	179.06505	27.38785	-2.108326	0.1529492	XR_242755	XR_242755	ENST00000450890
NM_007109	TCF19	186.31285	28.49385	-2.108428	0.1529355	NM_007109	NM_007109	ENST00000400401
NM_022908	NT5DC2	2134.37	591.30497	-2.110728	0.2770396	NM_022908	NM_022908	ENST00000478091
NM_001008535	AKAP14	69.955095	10.675528	-2.110896	0.1526054	NM_001008535	NM_001008535	ENST00000371422
ENST00000441085		81.140935	12.37699	-2.111409	0.1525369			ENST00000441085
NM_052941	GBP4	489.856	102.76361	-2.112177	0.2097833	NM_052941	NM_052941	ENST00000471938
NM_015401	HDAC7	730.3949	201.9702	-2.113807	0.2765219	NM_015401	NM_015401	ENST00000459625
NM_004729	ZBED1	372.5625	78.063525	-2.113812	0.2095314	NM_004729	NM_004729	ENST00000515319
NM_001123168	FAM72A	392.18605	82.162625	-2.114021	0.2094991	NM_001123168	NM_001123168	ENST00000470041
NM_017785	SPDL1	1248.1144	344.88679	-2.114972	0.2763263	NM_017785	NM_017785	ENST00000265295
NM_001254	CDC6	72.871725	11.051748	-2.117992	0.1516603	NM_001254	NM_001254	ENST00000209728
NM_001406	EFNB3	699.7815	146.02445	-2.119407	0.2086715	NM_001406	NM_001406	ENST00000226091
NM_148170	CTSC	950.2752	259.85065	-2.132209	0.2734478	NM_148170	NM_148170	ENST00000524463
NM_194255	SLC19A1	731.54555	199.98165	-2.132685	0.2733687	NM_194255	NM_194255	ENST00000477688
NM_002125	HLA-DRB5	1187.7884	324.3698	-2.13438	0.2730872	NM_002125	NM_002125	ENST00000374975
NM_001135844	SIRPB1	103.96313	15.535555	-2.13489	0.1494333	NM_001135844	NM_001135844	ENST00000381596
NM_001540	HSPB1	762.0328	207.92685	-2.135762	0.2728581	NM_001540	NM_001540	ENST00000248553
NM_002121	HLA-DPB1	401.1513	82.686975	-2.136118	0.2061242	NM_002121	NM_002121	ENST00000399500
ENST00000411955	SAMD9L	1358.779	370.48395	-2.136961	0.2726595	XM_005250193	XM_005250193	ENST00000411955
NM_001218	CA12	1072.0648	292.25985	-2.137235	0.272614	NM_001218	NM_001218	ENST00000178638
NR_110162	LOC101928036	798.19255	217.3169	-2.139367	0.2722612	NR_110162		ENST00000451962
NM_001142571	RAD51D	85.142825	12.658635	-2.140699	0.1486753	NM_001142571	NM_001142571	ENST00000345365
NM_153360	APCDD1L	2441.657	663.68135	-2.142061	0.271816	NM_153360	NM_153360	ENST00000371149
NM_002381	MATN3	117.73258	17.478915	-2.142332	0.1484629	NM_002381	NM_002381	ENST00000421259
ENST00000428292		67.356815	9.991385	-2.143315	0.1483352			ENST00000428292
NR_109825	NR2F1-AS1	519.6122	106.41888	-2.144857	0.2048044	NR_109825	NR_109825	
NM_001178056	PARP8	396.1866	80.95858	-2.147915	0.2043446	NM_001178056	NM_001178056	ENST00000440862
NM_007101	SARDH	742.7336	201.06535	-2.148772	0.2707099	NM_007101	NM_007101	ENST00000422262
NM_001114120	DEPDC1	77.5861	3.207888	-2.148777	0.0413462	NM_001114120	NM_001114120	ENST00000370966
NM_001034	RRM2	1498.4365	405.58375	-2.149007	0.2706713	NM_001034	NM_001034	ENST00000360566
NM_012449	STEAP1	1663.297	449.23475	-2.152565	0.2700869	NM_012449	NM_012449	ENST00000475789
NM_017831	RNF125	74.117965	10.905355	-2.152593	0.1471351	NM_017831	NM_017831	ENST00000217740
NM_021643	TRIB2	163.1836	23.98219	-2.153919	0.1469645	NM_021643	NM_021643	ENST00000155926
NM_018181	ZNF532	94.02501	13.81797	-2.153949	0.1469606	NM_018181	NM_018181	ENST00000591230
NM_052947	ALPK2	233.14065	47.426015	-2.154069	0.2034223	NM_052947	NM_052947	ENST00000361673
NM_004503	HOXC6	2489.6541	671.56711	-2.154661	0.2697431	NM_004503	NM_004503	ENST00000243108
NM_001079528	ABCC6	109.77406	16.113975	-2.155259	0.1467922	NM_001079528	NM_001079528	ENST00000542005
NR_026955	LINC00284	93.06477	3.81135	-2.155645	0.0409537	NR_026955	NR_026955	ENST00000423211
		103.69236	4.2335185	-2.157865	0.0408277			
ENST00000496217		85.041805	3.465463	-2.159234	0.0407501			ENST00000496217
NM_001012985	COA6	7754.1615	2447.0805	-2.162038	0.3155829	NM_001012985	NM_001012985	ENST00000619305
ENST00000602736		218.33285	44.134945	-2.162638	0.2021452			ENST00000602736
NR_036533	LOC100499489	589.10665	119.08364	-2.162655	0.2021428	NR_036533	NR_036533	
NM_017637	BNC2	634.66525	128.11605	-2.164532	0.201864	NM_017637	NM_017637	ENST00000380672
NM_001008223	C1QL4	88.57875	12.876535	-2.166393	0.1453682	NM_001008223	NM_001008223	ENST00000334221
NM_001013615	LURAP1	67.424095	9.7954945	-2.167072	0.1452818	NM_001013615	NM_001013615	ENST00000371980
NM_018455	CENPN	6034.8888	1898.9265	-2.167543	0.3146581	NM_018455	NM_018455	ENST00000299572
ENST00000444184	LINC00963	729.7156	195.16375	-2.168703	0.2674518			ENST00000444184
NR_109825	NR2F1-AS1	462.43105	93.02654	-2.169228	0.2011685	NR_109825	NR_109825	
ENST00000439784		102.88839	14.908075	-2.170113	0.1448956			ENST00000439784
NM_001031709	RNLS	58.8201	8.5044095	-2.172577	0.1445834	NM_001031709	NM_001031709	ENST00000466945
NM_000433	NCF2	107.7293	15.559885	-2.173749	0.144435	NM_000433	NM_000433	ENST00000413720
NM_016343	CENPF	340.2538	68.178205	-2.174608	0.2003746	NM_016343	NM_016343	ENST00000614578
NM_020692	GALNT16	721.889	144.5051	-2.175955	0.2001763	NM_020692	NM_020692	ENST00000553471
		88.91998	3.539439	-2.176137	0.0398048			
NM_005320	HIST1H1D	5077.441	1589.687	-2.176924	0.3130882	NM_005320	NM_005320	ENST00000244534
NM_016445	PLEK2	489.0408	97.677157	-2.178977	0.1997321	NM_016445	NM_016445	ENST00000216446
ENST00000568553		6236.8595	1949.6875	-2.179808	0.3126073			ENST00000568553
NM_022842	CDCP1	5847.2539	1824.9146	-2.182868	0.3120977	NM_022842	NM_022842	ENST00000296129

NM_003152	STAT5A	82.57187	11.831105	-2.182901	0.1432825	NM_003152	NM_003152	ENST00000345506
NM_031938	BCO2	422.3634	83.934135	-2.185856	0.1987249	NM_031938	NM_031938	ENST00000438022
XR_110828	LOC284561	89.717755	3.5206585	-2.1864	0.0392415	XR_110828	XR_110828	
NM_201649	SLC6A9	1829.6095	483.12697	-2.189711	0.2640602	NM_201649	NM_201649	ENST00000372310
NM_024036	LRFN4	3690.113	1147.1639	-2.190231	0.310875	NM_024036	NM_024036	ENST00000309602
NR_026915	AADACP1	550.57215	109.05056	-2.190363	0.1980677	NR_026915		ENST00000483636
NM_001080474	C2orf78	93.435325	3.643956	-2.190849	0.0389998	NM_001080474	NM_001080474	ENST00000409561
NM_152243	CDC42EP1	13742.185	4873.433	-2.192903	0.3546331	NM_152243	NM_152243	ENST00000249014
NM_016113	TRPV2	641.0541	126.64165	-2.193908	0.1975522	NM_016113	NM_016113	ENST00000338560
NM_022840	METTL4	359.5986	70.943175	-2.195754	0.1972843	NM_022840	NM_022840	ENST00000573134
NM_005213	CSTA	344.88679	67.94788	-2.197613	0.197015	NM_005213	NM_005213	ENST00000264474
NR_073432	CSAG4	116.16049	16.40459	-2.199434	0.1412235	NR_073432	NR_073432	ENST00000361201
NM_004843	IL27RA	297.67125	58.5527	-2.199772	0.1967026	NM_004843	NM_004843	ENST00000263379
ENST00000604106		88.24631	3.399053	-2.199804	0.0385178			ENST00000604106
NM_170735	BDNF	2561.1336	791.6591	-2.200941	0.309105	NM_170735	NM_170735	ENST00000525528
NM_003810	TNFSF10	101.6428	3.9057655	-2.201515	0.0384264	NM_003810	NM_003810	ENST00000241261
NM_006065	SIRPB1	196.23425	27.655455	-2.201804	0.1409308	NM_006065	NM_006065	ENST00000562014
NM_002852	PTX3	2878.6321	889.10867	-2.202398	0.308865	NM_002852	NM_002852	ENST00000295927
NM_005804	DDX39A	9555.9955	3372.935	-2.202781	0.3529653	NM_005804	NM_005804	ENST00000242776
NM_017637	BNC2	332.61485	65.2311	-2.203836	0.196116	NM_017637	NM_017637	ENST00000545497
NM_021023	CFHR3	81.96104	11.528687	-2.203996	0.1406606	NM_021023	NM_021023	ENST00000367427
NM_001001665	CYP27C1	88.42347	3.3745625	-2.206456	0.0381637	NM_001001665	NM_001001665	ENST00000409327
NM_198468	MMS22L	461.3523	90.284995	-2.20675	0.1956964	NM_198468	NM_198468	ENST00000275053
NM_001826	CKS1B	2391.1205	624.52315	-2.207736	0.2611843	NM_001826	NM_001826	ENST00000600888
NM_000043	FAS	395.56425	77.2914	-2.208845	0.1953953	NM_000043	NM_000043	ENST00000612663
NM_001114734	PABPC4L	166.80465	23.36286	-2.208874	0.1400612	NM_001114734	NM_001114734	ENST00000421491
NM_014783	ARHGAP11A	96.5459	13.501435	-2.210641	0.1398447	NM_014783	NM_014783	ENST00000361627
ENST00000424308		122.39317	17.107255	-2.211227	0.139773			ENST00000424308
NM_000946	PRIM1	607.89705	118.55704	-2.211404	0.1950281	NM_000946	NM_000946	ENST00000338193
NM_152562	CDCA2	2657.35	692.44835	-2.211558	0.2605785	NM_152562	NM_152562	ENST00000521098
ENST00000478294		716.66875	139.7511	-2.211593	0.195001			ENST00000478294
NM_002276	KRT19	164.90595	23.01545	-2.21291	0.1395671	NM_002276	NM_002276	ENST00000361566
NM_133493	CD109	4349.3555	1334.655	-2.214597	0.3068627	NM_133493	NM_133493	ENST00000287097
NR_033878	LINC00944	769.29455	149.56435	-2.21567	0.1944175	NR_033878	NR_033878	ENST00000540684
ENST00000586949	LOC100130938	620.13035	120.49596	-2.216441	0.1943075	XR_110148	XR_110148	ENST00000586949
NM_177403	RAB7B	120.08723	16.69526	-2.217347	0.1390261	NM_177403	NM_177403	ENST00000617991
NM_018186	C1orf112	1775.11	460.84795	-2.217646	0.2596166	NM_018186	NM_018186	ENST00000413811
NM_032704	TUBA1C	161439.3	98247.58	-2.218566	0.6085729	NM_032704	NM_032704	ENST00000552448
NM_002266	KPNA2	39424.995	17869.335	-2.218917	0.4532489	NM_002266	NM_002266	ENST00000582898
NM_030925	CAB39L	194.3735	37.55839	-2.224021	0.1932279	NM_030925	NM_030925	ENST00000410043
NM_001163724	SMIM1	161.12965	22.252945	-2.224933	0.1381058	NM_001163724	NM_001163724	ENST00000444870
NM_004153	ORC1	73.38845	10.126486	-2.225935	0.1379847	NM_004153	NM_004153	ENST00000371566
ENST00000449012	LOC101927787	335.50775	64.61994	-2.228425	0.1926034			ENST00000449012
NM_033446	MVB12B	2171.5915	559.7042	-2.229592	0.2577392	NM_033446	NM_033446	ENST00000361171
NM_006607	PTTG2	9194.935	2798.3445	-2.230109	0.3043354	NM_006607	NM_006607	ENST00000504686
NM_152369	SLC44A3	86.79248	11.931915	-2.230151	0.1374764	NM_152369	NM_152369	ENST00000446120
NM_013401	RAB3IL1	130.786	17.973015	-2.230594	0.1374231	NM_013401	NM_013401	ENST00000394836
NM_001569	IRAK1	3514.447	1067.4991	-2.233746	0.303746	NM_001569	NM_001569	ENST00000369980
NM_032822	FAM136A	4052.1447	1228.5947	-2.237144	0.3031962	NM_032822	NM_032822	ENST00000460307
NM_177963	SYT12	75.364675	10.284775	-2.238571	0.1364668	NM_177963	NM_177963	ENST00000393946
NM_001291774	TNNT1	19853.515	6887.416	-2.239033	0.3469117	NM_001291774	NM_001291774	ENST00000588981
NM_002128	HMGB1	23322.595	8088.4765	-2.239656	0.3468086	NM_002128	NM_002128	ENST00000522557
NM_030641	APOL6	1172.1503	300.2266	-2.239881	0.2561332	NM_030641	NM_030641	ENST00000409652
lnc-TFAP2D-1:1	lnc-TFAP2D-1	82.30509	11.21246	-2.24055	0.1362305			
NM_001042550	SMC2	329.80515	62.909075	-2.241593	0.1907483	NM_001042550	NM_001042550	ENST00000374787
NM_139211	HOPX	87.46287	3.1784585	-2.241703	0.0363407	NM_139211	NM_139211	ENST00000508121
ENST00000222728	HOXA6	374.2033	71.354735	-2.242049	0.1906844	XM_006715716	XM_006715716	ENST00000222728
NM_006120	HLA-DMA	749.7911	191.6215	-2.243527	0.2555665	NM_006120	NM_006120	ENST00000412394
NM_005242	F2RL1	5324.143	1608.0805	-2.244338	0.3020356	NM_005242	NM_005242	ENST00000296677
NM_001011724	HNRNPA1L2	18207.8	6292.591	-2.24698	0.3455986	NM_001011724	NM_001011724	ENST00000504082
NM_002162	ICAM3	1185.052	302.1567	-2.247351	0.2549734	NM_002162	NM_002162	ENST00000587992
NM_020396	BCL2L10	97.455165	3.5126225	-2.247616	0.0360435	NM_020396	NM_020396	ENST00000260442
NM_005101	ISG15	40852.94	18308.34	-2.250976	0.4481523	NM_005101	NM_005101	ENST00000379389
NM_003740	KCNK5	135.2482	18.25237	-2.251298	0.1349546	NM_003740	NM_003740	ENST00000359534
NM_021120	DLG3	711.89895	134.40225	-2.255604	0.188794	NM_021120	NM_021120	ENST00000542398
NM_016229	CYB5R2	217.3169	29.18207	-2.256993	0.1342835	NM_016229	NM_016229	ENST00000526084
NM_173083	LIN9	636.28785	119.8516	-2.258731	0.1883607	NM_173083	NM_173083	ENST00000328205
NM_003686	EXO1	567.00766	106.79321	-2.258842	0.1883453	NM_003686	NM_003686	ENST00000518741
NM_007317	KIF22	1234.4128	312.1353	-2.261043	0.2528614	NM_007317	NM_007317	ENST00000160827
NM_003202	TCF7	524.33579	98.583467	-2.261223	0.1880159	NM_003202	NM_003202	ENST00000522653
NM_000407	GP1BB	578.05905	108.64892	-2.261666	0.1879547	NM_000407	NM_000407	ENST00000366425
NM_007183	PKP3	8363.9025	2497.67	-2.265639	0.298625	NM_007183	NM_007183	ENST00000331563
NM_018154	ASF1B	1165.713	293.27825	-2.269359	0.251587	NM_018154	NM_018154	ENST00000263382
NM_022154	SLC39A8	3988.2655	1188.4208	-2.269698	0.2979794	NM_022154	NM_022154	ENST00000394833
NM_001282431	ARL4C	6828.72	2034.434	-2.270052	0.2979232	NM_001282431	NM_001282431	ENST00000390645
NM_032777	GPR124	214.3014	39.89636	-2.274652	0.1861694	NM_032777	NM_032777	ENST00000412232
NM_213602	SIGLEC15	1095.2483	274.42685	-2.276084	0.2505613	NM_213602	NM_213602	ENST00000389474
NM_006342	TACC3	1023.3565	256.3652	-2.276394	0.2505141	NM_006342	NM_006342	ENST00000470808
NM_001040129	SPINK13	95.004	3.281551	-2.278274	0.0345412	NM_001040129	NM_001040129	ENST00000511106
NM_002508	NID1	108.64892	3.7225235	-2.284119	0.0342619	NM_002508	NM_002508	ENST00000366595
NM_205843	NFIC	2015.2935	502.1834	-2.285142	0.2491862	NM_205843	NM_205843	ENST00000589164
ENST00000455845		112.74773	3.8573655	-2.285162	0.0342124			ENST00000455845
NM_001270408	JAM2	208.6193	38.42728	-2.289135	0.1841981	NM_001270408	NM_001270408	ENST00000460679
NM_018369	DEPDC1B	391.893	72.14848	-2.289841	0.1841025	NM_018369	NM_018369	ENST00000265036
NM_001007231	ARHGAP25	110.00385	14.315265	-2.292844	0.1301342	NM_001007231	NM_001007231	ENST00000409202
ENST00000447329		59.750275	7.772337	-2.293317	0.1300804			ENST00000447329
ENST00000453568	LOC101927787	92.81646	12.06663	-2.293977	0.1300053			ENST00000453568
NM_006495	EVI2B	114.99957	3.8696485	-2.297114	0.0336492	NM_006495	NM_006495	ENST00000577894
NM_004217	AURKB	564.8954	103.31857	-2.298768	0.1828986	NM_004217	NM_004217	ENST00000580390
NM_002278	KRT32	180.1115	23.316085	-2.298834	0.1294536	NM_002278	NM_002278	ENST00000225899
NM_022909	CENPH	732.02402	180.78239	-2.299898	0.2469624	NM_022909	NM_022909	ENST00000283006
NM_014256	B3GNT3	1083.4492	267.4122	-2.300876	0.2468156	NM_014256	NM_014256	ENST00000318683

lnc-MLXIP-1:1	lnc-MLXIP-1	78.02682	10.024091	-2.307548	0.1284698			
NM_001733	C1R	6442.7835	1879.5965	-2.309411	0.2917367	NM_001733	NM_001733	ENST00000540394
NR_026915	AADACP1	687.46255	168.75065	-2.309883	0.2454689	NR_026915		ENST00000483636
NM_002494	NDUFC1	26710.455	8955.5735	-2.310479	0.3352834	NM_002494	NM_002494	ENST00000394223
NM_012310	KIF4A	281.79863	51.0906	-2.310698	0.1813018	NM_012310	NM_012310	ENST00000374403
NM_030920	ANP32E	1406.2415	344.17675	-2.314714	0.2447494	NM_030920	NM_030920	ENST00000616917
NR_039985	FLJ22447	76.556615	9.769674	-2.315185	0.1276137	NR_039985		ENST00000556717
NM_000393	COL5A2	9148.9085	2653.882	-2.320117	0.2900764	NM_000393	NM_000393	ENST00000618828
NM_001040443	PHF11	698.55405	125.76495	-2.320229	0.1800361	NM_001040443	NM_001040443	ENST00000495157
NM_020754	ARHGAP31	64.1743	8.149966	-2.320715	0.1269973	NM_020754	NM_020754	ENST00000264245
NM_205860	NR5A2	62.434525	7.928229	-2.320829	0.1269847	NM_205860	NM_205860	ENST00000544748
NM_148920	PIGQ	63.08144	7.994848	-2.323046	0.1267385	NM_148920	NM_148920	ENST00000409439
NM_021258	IL22RA1	198.95955	25.167621	-2.325232	0.1264962	NM_021258	NM_021258	ENST00000270800
NR_033878	LINC00944	509.6134	91.385625	-2.325626	0.1793234	NR_033878	NR_033878	ENST00000540684
NR_023382	ZNF815P	122.39565	15.47469	-2.325814	0.1264317	NR_023382		ENST00000434898
NM_030771	CCDC34	2322.73	564.0708	-2.32755	0.2428482	NM_030771	NM_030771	ENST00000328697
NM_000407	GP1BB	1197.0201	290.45255	-2.328919	0.2426463	NM_000407	NM_000407	ENST00000431044
NM_017669	ERCC6L	366.3944	65.517775	-2.329469	0.1788176	NM_017669	NM_017669	ENST00000334463
NR_002165	HMGB3P1	2512.804	609.07195	-2.330677	0.2423874	NR_002165	NR_002165	ENST00000393368
NM_021105	PLSCR1	1500.0425	363.54385	-2.330892	0.2423557	NM_021105	NM_021105	ENST00000493432
ENST00000377803	HIST1H4C	143.4133	18.044175	-2.33136	0.1258194		BC130558	ENST00000377803
NR_015410	CASC15	196.82135	24.76132	-2.331481	0.1258061	NR_015410	NR_015410	ENST00000607048
NM_001129728	PLEKHG4	761.68265	184.3535	-2.333075	0.2420345	NM_001129728	NM_001129728	ENST00000393966
NM_053064	GNG2	605.62725	107.96692	-2.33362	0.1782729	NM_053064	NM_053064	ENST00000335281
ENST00000513853		97.96474	12.28276	-2.335361	0.1253794			ENST00000513853
NM_001039535	SKA1	267.14195	47.55519	-2.335592	0.1780147	NM_001039535	NM_001039535	ENST00000616604
NM_018342	TMEM144	234.49665	41.61468	-2.339808	0.1774639	NM_018342	NM_018342	ENST00000511532
NM_024781	CCDC102B	93.874055	11.71795	-2.340411	0.1248263	NM_024781	NM_024781	ENST00000360242
NM_145032	FBXL13	116.48791	14.53734	-2.34068	0.124797	NM_145032	NM_145032	ENST00000455112
NR_001587	AURKAPS1	1103.7658	265.84895	-2.341107	0.2408563	NR_001587	NR_001587	ENST00000462353
NM_001174104	CD14	3956.197	1133.6446	-2.343065	0.2865491	NM_001174104	NM_001174104	ENST00000302014
NM_006293	TYRO3	698.68135	168.0676	-2.343204	0.2405497	NM_006293	NM_006293	ENST00000568490
NM_030919	FAM83D	3165.527	906.25655	-2.344766	0.2862893	NM_030919	NM_030919	ENST00000217429
NM_005192	CDKN3	6864.613	1965.1005	-2.344924	0.2862653	NM_005192	NM_005192	ENST00000555837
NM_015651	PHF19	633.9369	112.05531	-2.345208	0.176761	NM_015651	NM_015651	ENST00000373896
NM_006012	CLPP	15831.045	5212.9965	-2.348281	0.3292895	NM_006012	NM_006012	ENST00000245816
NM_152479	TTC9B	66.01087	8.175473	-2.349377	0.1238504	NM_152479	NM_152479	ENST00000311308
NM_001207014	SERPINH1	15422.86	5074.8095	-2.349839	0.3290446	NM_001207014	NM_001207014	ENST00000524558
ENST00000439703	LOC101929484	1831.6645	438.79305	-2.349992	0.2395597	XR_245642	XR_245642	ENST00000439703
NR_049793	LOC100130476	94.63011	11.703555	-2.350978	0.1236769	NR_049793		
NM_001142651	NEURL1B	803.1106	141.1431	-2.353046	0.1757455	NM_001142651	NM_001142651	ENST00000369800
NM_001255	CDC20	1858.77	444.10015	-2.354383	0.2389215	NM_001255	NM_001255	ENST00000372462
NM_001114	ADCY7	615.50225	108.02802	-2.354856	0.175512	NM_001114	NM_001114	ENST00000254235
ENST00000303142	C20orf196	1079.1245	257.70225	-2.355174	0.2388068	XM_006723549	XM_006723549	ENST00000303142
NM_018284	GBP3	696.6837	122.04251	-2.35746	0.1751764	NM_018284	NM_018284	ENST00000394662
NM_178550	C1orf110	95.57935	11.748235	-2.358027	0.122916	NM_178550	NM_178550	ENST00000367910
NR_110826	LOC102724550	105.65273	3.2618665	-2.359111	0.0308735	NR_110826	NR_110826	ENST00000434309
NM_005250	FOXL1	273.4668	47.716655	-2.362817	0.1744879	NM_005250	NM_005250	ENST00000320241
NM_021156	TMX4	9681.8145	3165.527	-2.363184	0.326956	NM_021156	NM_021156	ENST00000246024
NM_006082	TUBA1B	129321.7	55700.17	-2.363521	0.4307102	NM_006082	NM_006082	ENST00000547765
NM_006065	SIRPB1	186.23425	22.74005	-2.365593	0.1221046	NM_006065	NM_006065	ENST00000262929
NM_000245	MET	35099.615	15103.98	-2.366106	0.4303175	NM_000245	NM_000245	ENST00000397752
NM_003537	HIST1H3B	5334.085	1509.1895	-2.366885	0.2829332	NM_003537	NM_003537	ENST00000621411
NM_003896	ST3GAL5	651.5535	113.1526	-2.369242	0.1736659	NM_003896	NM_003896	ENST00000461206
ENST00000449466	LOC101929484	3733.2285	1054.207	-2.370524	0.2823848	XR_245642	XR_245642	ENST00000449466
NM_021156	TMX4	7825.037	2205.32	-2.374221	0.2818287	NM_021156	NM_021156	ENST00000246024
NM_002164	IDO1	91.866198	11.108121	-2.376763	0.1209163	NM_002164	NM_002164	ENST00000523779
NM_001793	CDH3	73.16728	8.845642	-2.376953	0.1208961	NM_001793	NM_001793	ENST00000429102
NM_022648	TNS1	557.9213	96.22723	-2.378608	0.1724746	NM_022648	NM_022648	ENST00000171887
NM_018993	RIN2	2616.9465	735.36415	-2.379739	0.2810008	NM_018993	NM_018993	ENST00000255006
NM_194292	SASS6	72.021725	8.6774335	-2.380858	0.1204836	NM_194292	NM_194292	ENST00000462159
NM_020957	PCDHB16	327.4921	56.338715	-2.382113	0.1720308	NM_020957	NM_020957	ENST00000609684
NM_018248	NEIL3	106.12483	12.769255	-2.382382	0.120323	NM_018248	NM_018248	ENST00000264596
NM_001256270	KIF22	1250.9014	293.6342	-2.38346	0.2347381	NM_001256270	NM_001256270	ENST00000160827
NM_014220	TM4SF1	6914.784	1938.443	-2.384201	0.2803331	NM_014220	NM_014220	ENST00000622124
NM_005574	LMO2	278.82854	47.857029	-2.385238	0.1716361	NM_005574	NM_005574	ENST00000257818
NM_001281773	ZMYND8	3411.5765	955.7742	-2.385386	0.2801562	NM_001281773	NM_001281773	ENST00000461685
NM_001547	IFIT2	331.33395	56.764185	-2.387745	0.1713202	NM_001547	NM_001547	ENST00000611722
NR_015377	PAX8-AS1	152.97995	18.177685	-2.396701	0.118824	NR_015377	NR_015377	
NM_001190839	MGP	67.29275	7.986609	-2.398043	0.1186845	NM_001190839	NM_001190839	ENST00000539261
NM_001624	AIM1	501.1579	85.198755	-2.398239	0.1700038	NM_001624	NM_001624	ENST00000369066
NM_007047	BTN3A2	1334.6935	310.3153	-2.399234	0.2324993	NM_007047	NM_007047	ENST00000356386
NM_000584	CXCL8	2714.412	753.9874	-2.401417	0.2777719	NM_000584	NM_000584	ENST00000307407
NM_044472	CDC42	85.85074	10.156775	-2.401678	0.1183074	NM_044472	NM_044472	ENST00000315554
NM_152365	KDF1	613.1196	103.65247	-2.405833	0.1690575	NM_152365	NM_152365	ENST00000616918
NM_030920	ANP32E	1375.2755	318.3972	-2.406216	0.2315152	NM_030920	NM_030920	ENST00000616917
NM_001142279	RNASEH2B	2200.541	509.1922	-2.407077	0.2313941	NM_001142279	NM_001142279	ENST00000621641
NM_201563	FCGR2C	168.3681	19.81322	-2.407771	0.117678	NM_201563	NM_201563	ENST00000271450
ENST00000589177		3066.027	847.94285	-2.409613	0.2765608			ENST00000589177
NM_005225	E2F1	1019.732	235.38239	-2.411111	0.2308277	NM_005225	NM_005225	ENST00000343380
NM_001008397	GPX8	7878.2685	2176.474	-2.411634	0.276263	NM_001008397	NM_001008397	
NM_198282	TMEM173	9429.485	2602.346	-2.413559	0.2759797	NM_198282	NM_198282	ENST00000330794
NM_001017420	ESCO2	280.25765	47.092705	-2.414098	0.1680336	NM_001017420	NM_001017420	ENST00000397418
NM_001033	RRM1	10297.953	2839.5035	-2.415224	0.2757348	NM_001033	NM_001033	ENST00000532170
NM_001202435	SCN1A	124.50189	3.549173	-2.416539	0.028507	NM_001202435	NM_001202435	ENST00000303395
NM_052867	NALCN	404.207	67.77456	-2.417022	0.1676729	NM_052867	NM_052867	ENST00000251127
NM_001813	CENPE	1402.4976	322.01014	-2.419906	0.2295977	NM_001813	NM_001813	ENST00000611174
lnc-GOLGA8J-3:3	lnc-GOLGA8J-3	103.03959	11.99126	-2.420487	0.1163753			
ENST00000507639	LOC100506827	181.86775	21.15749	-2.420887	0.1163345			ENST00000507639
lnc-RP11-597K23.2.1-2:1	lnc-RP11-597K23.2.1-2	209.9142	24.397925	-2.421932	0.1162281			
NM_031942	CDCA7	1040.6499	238.54285	-2.422581	0.2292249	NM_031942	NM_031942	ENST00000467411
NM_032385	FAXDC2	321.88715	53.62824	-2.425709	0.1666057	NM_032385	NM_032385	ENST00000423554

NM_001006946	SDC1	14482.29	4594.313	-2.426422	0.3172366	NM_001006946	NM_001006946	ENST00000381150
NM_015967	PTPN22	78.986855	9.1414265	-2.426803	0.1157335	NM_015967	NM_015967	ENST00000538253
ENST00000567216	CAPN15	218.53105	25.13667	-2.433811	0.1150256	XM_006720935	XM_006720935	ENST00000567216
NM_177543	PPAP2C	3240.6134	883.90365	-2.435583	0.2727581	NM_177543	NM_177543	ENST00000621795
NM_152654	DAND5	94.380255	10.812558	-2.438407	0.1145638	NM_152654	NM_152654	ENST00000317060
NM_002317	LOX	906.58687	205.61961	-2.440041	0.2268063	NM_002317	NM_002317	ENST00000503759
NM_138461	TM4SF19	268.6207	44.266435	-2.440605	0.1647916	NM_138461	NM_138461	ENST00000273695
NM_002607	PDGFA	254.34093	41.874639	-2.441859	0.1646398	NM_002607	NM_002607	ENST00000402802
NM_057749	CCNE2	666.8267	109.64436	-2.443618	0.1644271	NM_057749	NM_057749	ENST00000308108
NM_004336	BUB1	2285.5635	517.113	-2.444069	0.2262519	NM_004336	NM_004336	ENST00000302759
NM_004688	NMI	1576.2987	356.47564	-2.444831	0.2261473	NM_004688	NM_004688	ENST00000243346
NM_033255	EPSTI1	72.046384	8.2059926	-2.445057	0.1138987	NM_033255	NM_033255	ENST00000476830
NM_024629	CENPU	2313.907	523.01445	-2.445678	0.2260309	NM_024629	NM_024629	ENST00000502461
NM_207380	C15orf52	14117.93	4437.5835	-2.445761	0.3143225	NM_207380	NM_207380	ENST00000382688
NM_005139	ANXA3	2745.4976	743.76628	-2.448377	0.270904	NM_005139	NM_005139	ENST00000505805
NM_053276	VIT	125.92015	3.4330125	-2.448659	0.0272634	NM_053276	NM_053276	ENST00000379242
NM_175884	CCDC71L	5335.2855	1444.246	-2.44981	0.270697	NM_175884	NM_175884	ENST00000315965
NM_001897	CSPG4	1086.1502	244.63155	-2.451534	0.2252281	NM_001897	NM_001897	ENST00000308508
NM_020987	ANK3	120.54893	3.252676	-2.456125	0.0269822	NM_020987	NM_020987	ENST00000280772
NM_001037161	ACOT1	2844.712	767.0457	-2.457155	0.2696391	NM_001037161	NM_001037161	ENST00000311148
NM_003199	TCF4	1228.2317	275.5961	-2.457712	0.2243845	NM_003199	NM_003199	ENST00000570177
NM_014288	ITGB3BP	1227.6296	275.41269	-2.458	0.2243451	NM_014288	NM_014288	ENST00000489863
NM_021134	MRPL23	5262.918	1417.067	-2.459829	0.269255	NM_021134	NM_021134	ENST00000381519
NR_036444	FENDRR	160.38665	18.03129	-2.459944	0.1124239	NR_036444	NR_036444	ENST00000598996
NM_004230	S1PR2	940.2719	210.26305	-2.463333	0.2236194	NM_004230	NM_004230	ENST00000589757
NM_018136	ASPM	439.0777	71.15203	-2.463441	0.1620488	NM_018136	NM_018136	ENST00000294732
NM_004613	TGM2	131.83495	14.743595	-2.465956	0.1118337	NM_004613	NM_004613	ENST00000469269
NM_001217	CA11	433.18925	69.903145	-2.469164	0.1613686	NM_001217	NM_001217	ENST00000084798
NM_001042517	DIAPH3	1184.6673	263.64015	-2.471271	0.2225436	NM_001042517	NM_001042517	ENST00000400324
NM_002916	RFC4	3140.9267	839.9308	-2.472691	0.267415	NM_002916	NM_002916	ENST00000296273
NM_182776	MCM7	15278.305	4739.586	-2.473314	0.3102167	NM_182776	NM_182776	ENST00000303887
NM_000693	ALDH1A3	36952.745	15293.8	-2.476561	0.4138745	NM_000693	NM_000693	ENST00000329841
NM_005325	HIST1H1A	732.6849	162.378	-2.478113	0.2216205	NM_005325	NM_005325	ENST00000244573
NM_001002029	C4B	1433.284	317.63795	-2.47815	0.2216155	NM_001002029	NM_001002029	ENST00000463249
NM_001072	UGT1A6	216.783	23.97958	-2.478466	0.1106156	NM_001072	NM_001072	ENST00000446481
NM_032977	CASP10	655.4198	104.96053	-2.479542	0.1601424	NM_032977	NM_032977	ENST00000346817
NM_032119	GPR98	228.40025	36.5637	-2.48002	0.1600861	NM_032119	NM_032119	ENST00000405460
NM_024808	BORA	727.66795	161.06995	-2.480117	0.2213509	NM_024808	NM_024808	ENST00000613797
NM_001098722	GNG4	2205.32	487.2408	-2.483184	0.2209388	NM_001098722	NM_001098722	ENST00000391854
NR_033878	LINC00944	619.83625	98.7484	-2.486601	0.1593137	NR_033878	NR_033878	
NM_138689	PPP1R14B	21295.445	6549.207	-2.491472	0.3075403	NM_138689	NM_138689	ENST00000309318
NM_001040708	HEY1	834.62855	183.2471	-2.493524	0.2195553	NM_001040708	NM_001040708	ENST00000337919
NM_017915	PARPBP	1280.37	280.5684	-2.49671	0.2191307	NM_017915	NM_017915	ENST00000327680
NM_001024215	FBLIM1	449.42555	70.86034	-2.500722	0.1576687	NM_001024215	NM_001024215	ENST00000441801
NR_036444	FENDRR	632.33375	99.541125	-2.502882	0.1574186	NR_036444	NR_036444	ENST00000594398
NM_007300	BRCA1	544.98418	85.780926	-2.503036	0.1574008	NM_007300	NM_007300	ENST00000357654
NM_032251	CCDC88B	957.06085	208.7608	-2.504174	0.2181393	NM_032251	NM_032251	ENST00000463837
NM_014959	CARD8	439.4673	69.051775	-2.505413	0.1571261	NM_014959	NM_014959	ENST00000391898
NM_001282936	STIL	890.9135	194.0093	-2.507005	0.2177645	NM_001282936	NM_001282936	ENST00000418131
NM_014791	MELK	2108.2135	458.6422	-2.508626	0.2175502	NM_014791	NM_014791	ENST00000298048
NM_178229	IQGAP3	1957.766	425.8456	-2.508884	0.2175161	NM_178229	NM_178229	ENST00000361170
NM_182513	SPC24	105.32431	11.27235	-2.516157	0.1070251	NM_182513	NM_182513	ENST00000423327
NM_020139	BDH2	98.583467	10.544497	-2.516852	0.1069601	NM_020139	NM_020139	
NM_001733	C1R	39498.015	11996.823	-2.517582	0.3037323	NM_001733	NM_001733	ENST00000602298
NM_004523	KIF11	1351.867	292.43235	-2.51798	0.2163174	NM_004523	NM_004523	ENST00000260731
NM_031958	KRTAP3-1	107.88917	11.521584	-2.51866	0.1067909	NM_031958	NM_031958	ENST00000580358
THC2651904		259.22925	40.33669	-2.518671	0.1556024			
NM_015429	ABI3BP	284.01765	44.082195	-2.522112	0.1552094	NM_015429	NM_015429	ENST00000284322
NM_003522	HIST1H2BF	546.795	84.77482	-2.523601	0.1550395	NM_003522	NM_003522	
NM_001013398	IGFBP3	958.04023	206.06009	-2.527384	0.215085	NM_001013398	NM_001013398	ENST00000275521
NM_006475	POSTN	487.2408	75.25861	-2.528707	0.1544588	NM_006475	NM_006475	ENST00000473823
NM_002221	ITPKB	480.05895	74.109425	-2.529439	0.1543757	NM_002221	NM_002221	ENST00000429204
NM_194313	KIF24	265.27225	40.915745	-2.53063	0.1542406	NM_194313	NM_194313	ENST00000379166
NM_015506	MMACHC	237.72285	36.629015	-2.532022	0.1540829	NM_015506	NM_015506	ENST00000616135
NM_025049	PIF1	681.64005	104.90334	-2.533652	0.1538984	NM_025049	NM_025049	ENST00000559239
NM_018370	DRAM1	17176.295	5174.436	-2.534747	0.3012545	NM_018370	NM_018370	ENST00000258534
NM_022168	IFIH1	1458.1275	311.9661	-2.536095	0.2139498	NM_022168	NM_022168	ENST00000263642
NM_013355	PKN3	289.7129	44.49902	-2.53632	0.153597	NM_013355	NM_013355	ENST00000291906
NM_002497	NEK2	369.51375	56.54629	-2.541361	0.1530289	NM_002497	NM_002497	ENST00000540251
NM_002417	MKI67	506.7961	77.5291	-2.541806	0.1529789	NM_002417	NM_002417	ENST00000617118
NM_005168	RND3	15734.306	4716.7748	-2.545053	0.2997765	NM_005168	NM_005168	ENST00000375734
NM_144647	CAPSL	90.08792	9.392505	-2.546064	0.1042593	NM_144647	NM_144647	ENST00000513623
NM_033066	MPP4	145.42005	15.154455	-2.546587	0.1042116	NM_033066	NM_033066	ENST00000359962
NM_018193	FANCI	1331.5958	283.05005	-2.546787	0.2125645	NM_018193	NM_018193	ENST00000566615
NM_024908	WDR76	93.1429	9.6992695	-2.547447	0.1041332	NM_024908	NM_024908	ENST00000478130
NM_031217	KIF18A	1128.6568	239.7697	-2.547767	0.2124381	NM_031217	NM_031217	ENST00000263181
NM_182751	MCM10	186.44825	19.397515	-2.548503	0.104037	NM_182751	NM_182751	ENST00000378694
NM_001234	CAV3	281.90675	42.906695	-2.548735	0.1522017	NM_001234	NM_001234	ENST00000397368
NM_001013254	LSP1	126.3191	13.13281	-2.549289	0.1039654	NM_001013254	NM_001013254	ENST00000406638
NM_006623	PHGDH	3105.003	797.062	-2.549377	0.2567025	NM_006623	NM_006623	ENST00000369407
NM_004219	PTTG1	35602.52	10638.245	-2.551849	0.2988059	NM_004219	NM_004219	ENST00000520452
NM_024769	CLMP	5647.995	1447.8455	-2.551978	0.2563468	NM_024769	NM_024769	ENST00000448775
NM_002358	MAD2L1	1955.6815	413.19565	-2.556767	0.2112796	NM_002358	NM_002358	ENST00000504707
NM_031157	HNRNPA1	56018.56	22514.785	-2.55968	0.4019165	NM_031157	NM_031157	ENST00000340913
NM_001103161	SH2D5	3117.289	794.89655	-2.561887	0.2549961	NM_001103161	NM_001103161	ENST00000375031
NM_021219	JAM2	84.24946	8.661076	-2.562135	0.1028027	NM_021219	NM_021219	ENST00000312957
NM_203394	E2F7	4573.7485	1165.1857	-2.563661	0.2547551	NM_203394	NM_203394	ENST00000416496
NR_036444	FENDRR	484.608	72.83208	-2.565926	0.1502907	NR_036444	NR_036444	ENST00000599749
NM_002160	TNC	340.14175	51.02681	-2.568412	0.1500163	NM_002160	NM_002160	ENST00000350763
NM_001423	EMP1	11303.491	3347.449	-2.570608	0.2961429	NM_001423	NM_001423	ENST00000256951
NM_001195228	FAM64A	645.11185	96.58977	-2.571051	0.1497256	NM_001195228	NM_001195228	ENST00000405197
NM_000612	IGF2	20476.32	6053.8935	-2.574075	0.2956534	NM_000612	NM_000612	ENST00000300632

NM_199329	SLC43A3	214.4218	21.805875	-2.574497	0.1016962	NM_199329	NM_199329	ENST00000525205
NM_152997	FDCSP	179.68555	18.267295	-2.574874	0.1016626	NM_152997	NM_152997	ENST00000317987
NM_004494	HDGF	7205.1285	1821.9405	-2.577613	0.2528672	NM_004494	NM_004494	ENST00000465180
NM_006113	VAV3	385.06145	56.893295	-2.589112	0.1477512	NM_006113	NM_006113	ENST00000370056
NM_002381	MATN3	86.7697	8.6716545	-2.594409	0.0999387	NM_002381	NM_002381	ENST00000407540
NM_001611	ACP5	7190.7285	1798.998	-2.59763	0.250183	NM_001611	NM_001611	ENST00000592828
NM_001185156	IL24	195.0438	19.38883	-2.600496	0.0994076	NM_001185156	NM_001185156	ENST00000294984
NR_121620	LOC100507420	289.1514	42.353135	-2.600925	0.1464739	NR_121620	NR_121620	ENST00000522718
NM_199327	SPRY1	343.9818	50.33691	-2.602207	0.146336	NM_199327	NM_199327	ENST00000339241
NM_032789	PARP10	4156.8415	1035.5387	-2.605641	0.2491167	NM_032789	NM_032789	ENST00000526007
NM_001001665	CYP27C1	253.92145	25.11095	-2.606429	0.0988926	NM_001001665	NM_001001665	
NM_000043	FAS	83.40461	8.222364	-2.609998	0.098584	NM_000043	NM_000043	ENST00000357339
NM_000186	CFH	254.9774	25.131545	-2.610232	0.0985638	NM_000186	NM_000186	ENST00000466229
NM_002837	PTPRB	126.18245	12.43177	-2.610715	0.0985222	NM_002837	NM_002837	ENST00000261266
NR_024431	ZMIZ1-AS1	818.04755	118.41496	-2.617003	0.1447532	NR_024431	NR_024431	
NM_003318	TTK	668.17785	96.705325	-2.617222	0.1447299	NM_003318	NM_003318	ENST00000504590
NM_001033580	MYO19	2110.125	429.6815	-2.617482	0.2036285	NM_001033580	NM_001033580	ENST00000621344
ENST00000444114		71.956295	7.0439995	-2.618036	0.0978927			ENST00000444114
NM_013277	RACGAP1	2804.542	693.9623	-2.618291	0.2474423	NM_013277	NM_013277	ENST00000454520
NM_182526	TMEM229B	648.60285	93.540255	-2.622042	0.1442181	NM_182526	NM_182526	ENST00000357461
NM_012449	STEAP1	3207.0555	791.5388	-2.623077	0.2468117	NM_012449	NM_012449	ENST00000297205
NM_031910	C1QTNF6	372.4122	53.35632	-2.630994	0.1432722	NM_031910	NM_031910	ENST00000493023
NM_198951	TGM2	725.9063	103.97601	-2.631337	0.1432361	NM_198951	NM_198951	ENST00000468262
NM_001236	CBR3	1199.9674	241.977	-2.633529	0.201653	NM_001236	NM_001236	ENST00000290354
NM_138456	BATF2	247.6025	23.88405	-2.634862	0.0964613	NM_138456	NM_138456	ENST00000527454
		722.98245	103.01445	-2.638487	0.1424854			
NM_002129	HMGB2	3757.332	918.54705	-2.640975	0.2444679	NM_002129	NM_002129	ENST00000511316
NR_121620	LOC100507420	880.0216	125.13352	-2.641275	0.1421937	NR_121620	NR_121620	
NM_014176	UBE2T	8426.519	2050.999	-2.649201	0.2433981	NM_014176	NM_014176	ENST00000367274
NM_016359	NUSAP1	4801.1905	1166.9786	-2.651806	0.2430602	NM_016359	NM_016359	ENST00000414849
NM_024902	DNAJC22	396.75585	55.683275	-2.659066	0.1403464	NM_024902	NM_024902	ENST00000395069
NM_002298	LCP1	524.7982	73.570425	-2.660603	0.140188	NM_002298	NM_002298	ENST00000323076
lnc-DNTTIP2-1:1	lnc-DNTTIP2-1	97.301515	9.1714145	-2.661259	0.0942577		DQ080207	
NM_173576	MKX	78.669555	7.4133915	-2.661539	0.0942346	NM_173576	NM_173576	ENST00000375790
NM_004934	CDH18	99.62678	9.3615205	-2.6648	0.0939659	NM_004934	NM_004934	ENST00000507958
NM_002497	NEK2	518.9178	72.435352	-2.666426	0.1395893	NM_002497	NM_002497	ENST00000462283
NM_058229	FBXO32	9497.6145	2289.7265	-2.667116	0.2410844	NM_058229	NM_058229	ENST00000287396
NM_001216	CA9	361.79715	50.468395	-2.667359	0.1394936	NM_001216	NM_001216	ENST00000617161
NM_018136	ASPM	2439.39	481.19985	-2.669764	0.1972624	NM_018136	NM_018136	ENST00000367408
NM_002417	MKI67	187.21415	17.50075	-2.670724	0.0934798	NM_002417	NM_002417	ENST00000617118
ENST00000334003	GSAP	112.90149	10.498895	-2.676705	0.0929916		AL834358	ENST00000334003
NM_018365	MNS1	141.50415	13.1145	-2.680549	0.0926793	NM_018365	NM_018365	ENST00000260453
NR_038393	LOC100506178	762.28055	105.2879	-2.680801	0.1381222	NR_038393	NR_038393	
NM_207418	FAM72D	803.60845	157.1213	-2.68437	0.1955197	NM_207418	NM_207418	ENST00000584486
NM_198275	MPZL3	217.07575	20.01254	-2.686576	0.0921915	NM_198275	NM_198275	ENST00000278949
NM_003335	UBA7	512.84157	70.532915	-2.686612	0.1375335	NM_003335	NM_003335	ENST00000333486
NM_018284	GBP3	1682.6765	327.9804	-2.689461	0.1949159	NM_018284	NM_018284	ENST00000489444
NM_013989	DIO2	244.96595	22.51754	-2.689931	0.0919211	NM_013989	NM_013989	ENST00000557010
NM_014226	MOK	1141.6705	222.34808	-2.690806	0.1947568	NM_014226	NM_014226	ENST00000193029
NM_007308	SNCA	2977.0707	704.96061	-2.700776	0.2367967	NM_007308	NM_007308	ENST00000508895
NR_027082	SFTA1P	194.9981	17.74963	-2.701126	0.0910246	NR_027082	NR_027082	ENST00000446372
lnc-DNTTIP2-1:1	lnc-DNTTIP2-1	256.80415	23.37307	-2.701245	0.0910152		DQ080207	
NM_001287682	LOC388780	497.36775	67.657525	-2.701556	0.1360312	NM_001287682	NM_001287682	ENST00000411839
NM_001129728	PLEKHG4	1825.8855	352.7711	-2.703969	0.1932055	NM_001129728	NM_001129728	ENST00000379344
NM_018685	ANLN	2014.784	388.72525	-2.706263	0.1929364	NM_018685	NM_018685	ENST00000491782
NM_019076	UGT1A8	202.3919	18.323065	-2.707317	0.0905326	NM_019076	NM_019076	ENST00000450233
NM_018039	KDM4D	86.708795	7.8438745	-2.708205	0.0904623	NM_018039	NM_018039	ENST00000610872
NM_198404	KCTD4	796.86265	106.60408	-2.724263	0.1337797	NM_198404	NM_198404	ENST00000379108
NM_006739	MCM5	602.97135	80.5344	-2.726473	0.1335626	NM_006739	NM_006739	ENST00000216122
NM_152308	RMI2	1520.993	289.41245	-2.729096	0.1902786	NM_152308	NM_152308	ENST00000572173
NM_002915	RFC3	421.95815	56.15894	-2.731283	0.1330913	NM_002915	NM_002915	ENST00000380071
NM_014298	QPRT	400.53515	53.13967	-2.735579	0.1326717	NM_014298	NM_014298	ENST00000219771
NM_182707	PSG8	137.1839	12.11126	-2.736035	0.0882848	NM_182707	NM_182707	ENST00000306511
NM_003544	HIST1H4B	13065.38	3569.1475	-2.739775	0.2731759	NM_003544	NM_003544	ENST00000377745
NM_014831	TRANK1	89.95337	7.9108915	-2.740449	0.0879444	NM_014831		ENST00000429976
NM_032587	CARD6	319.7248	42.2113	-2.742238	0.1320239	NM_032587	NM_032587	ENST00000254691
ENST00000586949	LOC100130938	1214.1054	228.8505	-2.744615	0.1884931	XR_110148	XR_110148	ENST00000586949
NM_018131	CEP55	370.39455	48.814005	-2.744659	0.1317892	NM_018131	NM_018131	ENST00000496302
NR_109804	RBMS3-AS3	89.740225	7.8610755	-2.744955	0.0875981	NR_109804	NR_109804	
NM_018951	HOXA10	259.5691	22.69837	-2.746936	0.0874463	NM_018951	NM_018951	ENST00000521421
NM_002894	RBBP8	3267.79	753.8862	-2.749684	0.2307022	NM_002894	NM_002894	ENST00000327155
NM_001164460	STEAP1B	3806.6825	877.3215	-2.751582	0.2304688	NM_001164460	NM_001164460	ENST00000621434
NM_025130	HKDC1	202.1806	17.60241	-2.751957	0.0870628	NM_025130	NM_025130	ENST00000354624
NM_001256877	KANK1	296.45145	38.758325	-2.755524	0.1307409	NM_001256877	NM_001256877	ENST00000382289
NM_001017420	ESCO2	302.95925	39.590185	-2.756176	0.1306783	NM_001017420	NM_001017420	ENST00000305188
NM_003202	TCF7	80.07604	6.9398225	-2.757182	0.0866654	NM_003202	NM_003202	ENST00000522561
NM_001288819	CPEB1	443.5502	57.547615	-2.765947	0.1297432	NM_001288819	NM_001288819	ENST00000620212
NM_152680	TMEM154	1195.4944	222.41655	-2.766127	0.1860457	NM_152680	NM_152680	ENST00000304385
NM_138555	KIF23	1076.2564	199.9344	-2.768582	0.1857684	NM_138555	NM_138555	ENST00000560125
NM_001258406	IRG1	124.98258	10.704538	-2.770668	0.0856482	NM_001258406	NM_001258406	ENST00000449753
ENST00000586949	LOC100130938	1557.135	288.88435	-2.770758	0.185523	XR_110148	XR_110148	ENST00000586949
NM_022770	GINS3	3569.1475	813.18845	-2.773114	0.2278383	NM_022770	NM_022770	ENST00000328514
NM_002581	PAPPA	513.5031	66.153575	-2.775578	0.128828	NM_002581	NM_002581	ENST00000328252
NM_007174	CIT	3704.8005	842.9019	-2.775768	0.2275161	NM_007174	NM_007174	ENST00000545913
NM_144573	NEXN	1670.0515	308.37537	-2.77852	0.1846502	NM_144573	NM_144573	ENST00000480732
lnc-GOLGA8J-3:2	lnc-GOLGA8J-3	79.84309	6.787178	-2.779259	0.0850065			
NM_001424	EMP2	495.25791	63.50114	-2.782032	0.1282183	NM_001424	NM_001424	ENST00000359543
NM_005098	MSC	194.47285	16.48539	-2.782446	0.0847696	NM_005098	NM_005098	ENST00000325509
NM_020242	KIF15	710.15685	90.61957	-2.788556	0.127605	NM_020242	NM_020242	ENST00000453693
NM_002315	LMO1	139.58285	11.726905	-2.792674	0.0840139	NM_002315	NM_002315	ENST00000335790
NM_005480	TROAP	2853.4445	522.1675	-2.793337	0.1829955	NM_005480	NM_005480	ENST00000257909
NM_000505	F12	1119.2704	204.7904	-2.793587	0.1829678	NM_000505	NM_000505	ENST00000253496

NM_001040147	SERPINB7	91.16154	7.6134595	-2.799463	0.0835161	NM_001040147	NM_001040147	ENST00000398019
NM_001425	EMP3	13705.175	3636.6515	-2.800694	0.2653488	NM_001425	NM_001425	ENST00000597057
NR_036444	FENDRR	932.7714	117.23105	-2.809233	0.1256804	NR_036444	NR_036444	ENST00000599749
ENST00000592540	SPC24	798.0993	100.14209	-2.81145	0.1254757		AK075287	ENST00000592540
NM_002417	MKI67	962.57525	174.12575	-2.812334	0.1808957	NM_002417	NM_002417	ENST00000617118
NR_036488	LINC00673	1090.2103	196.86705	-2.815235	0.1805771	NR_036488	NR_036488	ENST00000457958
ENST00000415536	LOC102723946	498.84675	62.308345	-2.817655	0.1249048			ENST00000415536
ENST00000426023		276.74125	22.72021	-2.81901	0.0820991			ENST00000426023
NM_001409	MEGF6	829.7701	103.31515	-2.821956	0.1245106	NM_001409	NM_001409	
NM_002575	SERPINB2	82.88763	6.7793575	-2.823322	0.0817897	NM_002575	NM_002575	ENST00000457692
NR_125805	TMEM92-AS1	147.21715	12.007265	-2.826513	0.0815616	NR_125805		
NM_016240	SCARA3	1340.38	239.7205	-2.831099	0.1788452	NM_016240	NM_016240	ENST00000301904
NM_003641	IFITM1	14238.71	3722.6335	-2.831756	0.2614446	NM_003641	NM_003641	ENST00000408968
NM_001042353	FAM110A	2938.854	524.1985	-2.835494	0.1783683	NM_001042353	NM_001042353	ENST00000304189
NM_000676	ADORA2B	6834.326	1503.013	-2.839452	0.2199212	NM_000676	NM_000676	ENST00000582124
NM_016235	GPRC5B	641.2144	78.529635	-2.844436	0.1224702	NM_016235	NM_016235	ENST00000569479
NM_014668	GREB1	145.9086	11.706035	-2.845336	0.0802285	NM_014668	NM_014668	ENST00000234142
NM_014831	TRANK1	267.82815	32.729465	-2.847405	0.1222032	NM_014831	NM_014831	ENST00000429976
BC112254	HIST1H2AI	77.633735	6.189856	-2.852434	0.0797315		BC112254	
NM_003258	TK1	58775.7	21280.89	-2.855681	0.3620695	NM_003258	NM_003258	ENST00000301634
ENST00000434919	SFTA1P	156.5218	12.409955	-2.858838	0.0792858			ENST00000434919
NM_002089	CXCL2	8075.6545	1755.8005	-2.860915	0.217419	NM_002089	NM_002089	ENST00000508487
NM_014331	SLC7A11	3123.1505	548.3311	-2.861523	0.1755699	NM_014331	NM_014331	ENST00000280612
NR_036444	FENDRR	183.29655	14.44659	-2.865635	0.0788154	NR_036444	NR_036444	ENST00000599749
NM_002089	CXCL2	6852.6935	1484.0135	-2.868348	0.2165592	NM_002089	NM_002089	ENST00000510048
NM_022047	DEF6	276.89645	33.316975	-2.868503	0.1203229	NM_022047	NM_022047	ENST00000316637
NM_006806	BTG3	3385.1425	731.27255	-2.872987	0.2160242	NM_006806	NM_006806	ENST00000339775
NM_001237	CCNA2	723.92226	86.812012	-2.873078	0.119919	NM_001237	NM_001237	ENST00000618014
NM_152495	CNIH3	155.18075	12.111915	-2.876776	0.0780504	NM_152495	NM_152495	ENST00000272133
NM_024869	FAM110D	296.52605	23.11975	-2.877972	0.0779687	NM_024869	NM_024869	ENST00000374268
NM_004237	TRIP13	3027.324	525.48815	-2.880269	0.1735817	NM_004237	NM_004237	ENST00000166345
NM_031966	CCNB1	2105.6745	365.4961	-2.880317	0.1735767	NM_031966	NM_031966	ENST00000256442
NM_022145	CENPK	277.1726	32.983975	-2.883526	0.1190016	NM_022145	NM_022145	ENST00000242872
NM_033066	MPP4	171.6836	13.297886	-2.885511	0.0774558	NM_033066	NM_033066	ENST00000359962
ENST00000470135	LOC340340	241.977	18.687198	-2.888888	0.0772272	XR_252283		ENST00000470135
NM_001826	CKS1B	23061.72	5865.1375	-2.889625	0.2543235	NM_001826	NM_001826	ENST00000477676
NM_145061	SKA3	983.03075	169.5561	-2.890721	0.172483	NM_145061	NM_145061	ENST00000298260
NM_138555	KIF23	5060.6135	1079.9673	-2.895856	0.2134064	NM_138555	NM_138555	ENST00000260363
NM_198573	ENHO	485.61175	57.10899	-2.89962	0.1176022	NM_198573	NM_198573	ENST00000399775
ENST00000396437	CENPM	1561.3615	267.76195	-2.9002	0.1714926	XR_244383	XR_244383	ENST00000396437
NM_000804	FOLR3	257.39285	19.604805	-2.904679	0.0761669	NM_000804	NM_000804	ENST00000622388
NM_004900	APOBEC3B	291.9594	34.1008	-2.908935	0.1167998	NM_004900	NM_004900	ENST00000335760
lnc-IL6-3:8	lnc-IL6-3	368.214	42.98848	-2.909531	0.1167486			
NR_121621	LOC100507420	96.41402	7.2411185	-2.920724	0.0751044	NR_121621	NR_121621	
NM_001018115	FANCD2	322.6274	37.288745	-2.923238	0.1155784	NM_001018115	NM_001018115	ENST00000581685
NM_003641	IFITM1	30713.33	7683.225	-2.924221	0.2501593	NM_003641	NM_003641	ENST00000408968
NM_005654	NR2F1	8952.969	1877.955	-2.928202	0.2097578	NM_005654	NM_005654	ENST00000615873
NM_005531	IFI16	2822.5985	474.53135	-2.932907	0.1681186	NM_005531	NM_005531	ENST00000562225
NM_001007232	CARD17	266.8197	19.779825	-2.935613	0.0741318	NM_001007232	NM_001007232	ENST00000375707
NM_001786	CDK1	2718.2941	455.58781	-2.937987	0.1676006	NM_001786	NM_001786	ENST00000448257
NM_002192	INHBA	2681.5761	447.91933	-2.943543	0.1670358	NM_002192	NM_002192	ENST00000442711
NM_001011724	HNRNPA1L2	37243.52	13068.58	-2.944554	0.3508954	NM_001011724	NM_001011724	ENST00000504082
NM_018304	PRR11	1307.8235	218.21065	-2.945373	0.1668502	NM_018304	NM_018304	ENST00000614081
NM_033260	FOXQ1	3666.799	761.68265	-2.946476	0.2077241	NM_033260	NM_033260	
NM_001290212	TSPAN10	5471.8255	1135.9911	-2.947531	0.2076073	NM_001290212	NM_001290212	ENST00000621293
NM_001540	HSPB1	52128.37	18267.945	-2.948223	0.3504415	NM_001540	NM_001540	ENST00000447574
NM_001289758	IFIT3	2849.265	473.7423	-2.951125	0.1662682	NM_001289758	NM_001289758	ENST00000371818
NR_036444	FENDRR	530.92335	59.8278	-2.957715	0.1126863	NR_036444	NR_036444	ENST00000594398
NM_016644	PRR16	85.420335	6.126295	-2.973403	0.0717194	NM_016644	NM_016644	ENST00000446965
NM_012310	KIF4A	1187.4933	194.59165	-2.975064	0.1638676	NM_012310	NM_012310	ENST00000374403
NM_014264	PLK4	159.26255	11.389352	-2.976694	0.0715131	NM_014264	NM_014264	ENST00000510192
NM_007280	OIP5	2198.504	359.7889	-2.977234	0.1636517	NM_007280	NM_007280	ENST00000220514
NM_182826	SCARA3	173.44335	12.39349	-2.977613	0.0714556	NM_182826	NM_182826	ENST00000337221
NM_001290050	UHRF1	315.67375	35.025733	-2.978775	0.1109555	NM_001290050	NM_001290050	ENST00000616255
NR_028044	IGF2-AS	413.34625	45.835395	-2.979595	0.1108886	NR_028044	NR_028044	
NM_003546	HIST1H4L	19571.005	4746.7195	-2.989054	0.2425384	NM_003546	NM_003546	
NM_020785	CC2D2A	97.555905	6.885388	-2.991713	0.0705789	NM_020785	NM_020785	ENST00000438599
NM_152649	MLKL	865.64245	94.737525	-2.997463	0.1094419	NM_152649	NM_152649	ENST00000308807
NM_014750	DLGAP5	3278.867	528.64255	-3.001803	0.1612272	NM_014750	NM_014750	ENST00000247191
NM_001142270	DHRS9	96.923115	6.777937	-3.002246	0.0699311	NM_001142270	NM_001142270	ENST00000428522
NM_001013841	STAP2	369.86525	40.331565	-3.002419	0.1090439	NM_001013841	NM_001013841	ENST00000597593
NM_021784	FOXA2	337.57435	36.782	-3.00347	0.1089597	NM_021784	NM_021784	ENST00000419308
NM_178460	SIRPD	314.67305	21.9715	-3.004008	0.0698233	NM_178460	NM_178460	ENST00000381623
NM_022346	NCAPG	326.9537	35.607935	-3.004114	0.1089082	NM_022346	NM_022346	ENST00000251496
NM_000204	CFI	888.94629	143.117	-3.004162	0.1609962	NM_000204	NM_000204	ENST00000512148
NM_033274	ADAM19	4116.8835	828.6171	-3.005653	0.2012729	NM_033274	NM_033274	ENST00000257527
NM_005282	GPR4	399.11925	43.37266	-3.007081	0.1086709	NM_005282	NM_005282	ENST00000323040
NM_014033	METTL7A	467.70475	50.75347	-3.009022	0.108516	NM_014033	NM_014033	ENST00000550097
NM_019055	ROBO4	365.7865	39.528025	-3.014712	0.1080631	NM_019055	NM_019055	ENST00000534407
NM_001540	HSPB1	63169.329	21609.69	-3.016592	0.3420915	NM_001540	NM_001540	ENST00000248553
NM_001114173	CTSC	5594.4765	1119.0132	-3.017355	0.2000211	NM_001114173	NM_001114173	ENST00000393301
NM_001353	AKR1C1	1789.17	285.0966	-3.021124	0.1593457	NM_001353	NM_001353	ENST00000477661
ENST00000414039		98.60344	6.7218545	-3.03137	0.0681706			ENST00000414039
NM_000575	IL1A	313.92179	21.280268	-3.037791	0.0677884	NM_000575	NM_000575	ENST00000263339
NM_003579	RAD54L	1351.328	211.18585	-3.053099	0.1562802	NM_003579	NM_003579	ENST00000371975
NM_000623	BDKRB2	2662.625	415.67785	-3.054832	0.1561158	NM_000623	NM_000623	ENST00000554311
NM_001850	COL8A1	374.11675	39.062025	-3.061484	0.1044113	NM_001850	NM_001850	ENST00000273342
NM_057749	CCNE2	88.401665	5.832831	-3.06866	0.065981	NM_057749	NM_057749	ENST00000611101
NM_199072	MDFIC	670.28145	69.474418	-3.071446	0.1036496	NM_199072	NM_199072	ENST00000257724
NM_052913	TMEM200A	6299.17	1221.3176	-3.075793	0.1938855	NM_052913	NM_052913	ENST00000392429
NM_016315	GULP1	421.88675	43.46565	-3.079646	0.1030268	NM_016315	NM_016315	ENST00000409580
NR_038393	LOC100506178	305.85395	19.931595	-3.082839	0.065167	NR_038393	NR_038393	

NR_036444	FENDRR	1263.0506	193.6139	-3.084892	0.1532907	NR_036444	NR_036444	ENST00000599749
ENST00000402338	CENPM	8659.6685	1661.25	-3.09571	0.1918376	XR_244383	XR_244383	ENST00000402338
NM_001285486	NEURL3	401.92255	40.820915	-3.0991	0.1015641	NM_001285486	NM_001285486	ENST00000451794
NM_005504	BCAT1	2830.7415	429.2001	-3.102919	0.1516211	NM_005504	NM_005504	ENST00000261192
ENST00000562272	PLK1	989.27865	99.8062	-3.10819	0.1008879		AK310544	ENST00000562272
NM_025029	MZT2B	93688.56	30937.015	-3.116798	0.3302112	NM_025029	NM_025029	ENST00000281871
NM_057159	LPAR1	795.86255	79.75778	-3.117287	0.1002155	NM_057159	NM_057159	ENST00000374430
NM_017821	RHBDL2	99.47303	6.2856915	-3.118031	0.0631899	NM_017821	NM_017821	ENST00000372990
NM_138689	PPP1R14B	15561.355	3547.8925	-3.118646	0.2279938	NM_138689	NM_138689	ENST00000309318
NM_000439	PCSK1	563.6531	56.426555	-3.118739	0.1001087	NM_000439	NM_000439	ENST00000311106
NR_036444	FENDRR	581.46265	58.151045	-3.120105	0.1000082	NR_036444	NR_036444	ENST00000598996
NM_018556	SIRPG	306.76035	30.61846	-3.122773	0.0998123	NM_018556	NM_018556	ENST00000381580
NM_006479	RAD51AP1	205.4618	12.85281	-3.129553	0.0625557	NM_006479	NM_006479	ENST00000535558
NM_005978	S100A2	3221.895	602.97135	-3.142132	0.187148	NM_005978	NM_005978	ENST00000368709
NM_001018115	FANCD2	272.7664	16.828675	-3.145354	0.0616963	NM_001018115	NM_001018115	ENST00000421731
NM_001547	IFIT2	4061.7105	756.46635	-3.151222	0.1862433	NM_001547	NM_001547	ENST00000611722
NM_021067	GINS1	4341.6135	808.1355	-3.152292	0.1861371	NM_021067	NM_021067	ENST00000262460
NM_001753	CAV1	13018.142	2905.5677	-3.163236	0.2231937	NM_001753	NM_001753	ENST00000341049
NM_001753	CAV1	9226.344	1706.868	-3.163792	0.1849994	NM_001753	NM_001753	ENST00000451122
NM_006187	OAS3	498.754	48.296025	-3.163997	0.0968334	NM_006187	NM_006187	ENST00000228928
		259.06755	15.717715	-3.164509	0.0606703			
NM_001067	TOP2A	1585.7549	229.67774	-3.178254	0.1448381	NM_001067	NM_001067	ENST00000423485
NM_012291	ESPL1	1303.448	188.0251	-3.184928	0.1442521	NM_012291	NM_012291	ENST00000552462
NM_001083	PDE5A	3261.685	468.62295	-3.191525	0.1436751	NM_001083	NM_001083	ENST00000394439
NM_012484	HMMR	5089.2795	923.54485	-3.199935	0.1814687	NM_012484	NM_012484	ENST00000358715
NM_005483	CHAF1A	3199.1005	580.24285	-3.200885	0.1813769	NM_005483	NM_005483	ENST00000301280
NM_002263	KIFC1	935.69145	133.42615	-3.203931	0.1425963	NM_002263	NM_002263	ENST00000480346
NM_002632	PGF	5204.1925	937.962	-3.212762	0.180232	NM_002632	NM_002632	ENST00000555567
NM_001005176	SP140	312.58485	29.076935	-3.218647	0.0930209	NM_001005176	NM_001005176	ENST00000544128
NM_001809	CENPA	1043.3777	96.118905	-3.231846	0.0921228	NM_001809	NM_001809	ENST00000233505
NM_002466	MYBL2	317.36205	29.127115	-3.236936	0.0917788	NM_002466	NM_002466	ENST00000396863
NM_024420	PLA2G4A	420.33867	38.463647	-3.240982	0.0915063	NM_024420	NM_024420	ENST00000367466
NR_104644	LINC01204	281.49765	15.935565	-3.243633	0.0566099	NR_104644		ENST00000609127
NM_031965	GSG2	146.4414	8.2760945	-3.245555	0.0565147	NM_031965	NM_031965	ENST00000325418
NM_001734	C1S	13832.24	2955.219	-3.254841	0.2136472	NM_001734	NM_001734	ENST00000360817
NM_018131	CEP55	1943.4427	266.8728	-3.265997	0.1373196	NM_018131	NM_018131	ENST00000371485
NM_144573	NEXN	1337.243	183.5207	-3.266974	0.1372381	NM_144573	NM_144573	ENST00000334785
NM_016817	OAS2	277.76135	15.395385	-3.267761	0.0554267	NM_016817	NM_016817	ENST00000620097
NM_001080522	CC2D2A	118.60173	6.5580845	-3.270477	0.055295	NM_001080522	NM_001080522	ENST00000389652
NM_016343	CENPF	12366.53	2614.124	-3.277128	0.211387	NM_016343	NM_016343	ENST00000366955
NM_022748	TNS3	2720.9905	370.56375	-3.279629	0.1361871	NM_022748	NM_022748	ENST00000311160
NM_016095	GINS2	1022.5289	139.13703	-3.281026	0.1360715	NM_016095	NM_016095	ENST00000253462
NM_025045	BAIAP2L2	4239.4296	734.6433	-3.286455	0.1732882	NM_025045	NM_025045	ENST00000381669
NR_103848	CASC9	131.91045	7.1557415	-3.292335	0.054247	NR_103848	NR_103848	ENST00000523313
NM_001424	EMP2	1184.2135	160.00702	-3.292617	0.1351167	NM_001424	NM_001424	ENST00000359543
NM_001009936	PHF19	5886.8605	1012.0538	-3.301353	0.1719174	NM_001009936	NM_001009936	ENST00000312189
NM_001789	CDC25A	345.3627	30.213445	-3.302154	0.0874832	NM_001789	NM_001789	ENST00000302506
NM_144652	LETM2	382.9433	33.46443	-3.303644	0.0873874	NM_144652	NM_144652	ENST00000526075
NM_002463	MX2	701.4739	60.673906	-3.317612	0.0864949	NM_002463	NM_002463	ENST00000330714
NM_006169	NNMT	26358.91	5454.6325	-3.321714	0.206937	NM_006169	NM_006169	ENST00000299964
NM_016448	DTL	1066.6732	141.58265	-3.321916	0.1327329	NM_016448	NM_016448	ENST00000475419
NM_002692	POLE2	376.5244	32.244505	-3.33117	0.0856372	NM_002692	NM_002692	ENST00000556584
NM_014226	MOK	18241.41	3756.3475	-3.331995	0.2059242	NM_014226	NM_014226	ENST00000361847
NM_005573	LMNB1	2642.2615	348.03	-3.334567	0.1317167	NM_005573	NM_005573	ENST00000460265
NM_024745	SHCBP1	1672.4475	219.9482	-3.337117	0.1315128	NM_024745	NM_024745	ENST00000303383
NM_031966	CCNB1	9477.8583	1598.4618	-3.33732	0.1686522	NM_031966	NM_031966	ENST00000256442
NM_001003927	EVI2A	167.0094	8.6435885	-3.346048	0.0517551	NM_001003927	NM_001003927	ENST00000578021
NM_001012409	SGOL1	395.76189	33.4806	-3.347784	0.0845978	NM_001012409	NM_001012409	ENST00000417364
NM_001030059	PPAPDC1A	954.39685	124.46921	-3.350895	0.1304166	NM_001030059	NM_001030059	ENST00000369073
NM_002309	LIF	1919.9185	249.83235	-3.35456	0.1301265	NM_002309	NM_002309	ENST00000249075
NM_001017534	CARD16	720.8127	60.290615	-3.363235	0.0836426	NM_001017534	NM_001017534	ENST00000525374
NM_001548	IFIT1	7926.306	1308.731	-3.377108	0.1651123	NM_001548	NM_001548	ENST00000371804
NR_027916	AKR1C8P	2196.1755	281.7075	-3.378189	0.1282719	NR_027916	NR_027916	ENST00000432689
NM_001978	DMTN	1326.988	170.09095	-3.379392	0.1281782	NM_001978	NM_001978	ENST00000358242
NM_001039670	IFFO1	1545.364	196.1948	-3.395149	0.126957	NM_001039670	NM_001039670	ENST00000396830
NM_153186	KANK1	1333.2605	168.5596	-3.40204	0.1264266	NM_153186	NM_153186	ENST00000382289
NM_003504	CDC45	2669.7	337.21055	-3.403556	0.1263103	NM_003504	NM_003504	ENST00000493724
NM_001511	CXCL1	16967.795	3369.8655	-3.407848	0.1986036	NM_001511	NM_001511	ENST00000395761
NM_198686	RAB15	1431.788	180.02525	-3.411075	0.1257346	NM_198686	NM_198686	ENST00000533601
NM_207315	CMPK2	288.15975	14.025785	-3.416166	0.0486736	NM_207315	NM_207315	ENST00000478738
NM_001856	COL16A1	1037.1835	129.9967	-3.416298	0.1253363	NM_001856	NM_001856	ENST00000488128
NM_016426	GTSE1	1187.8327	148.3929	-3.421676	0.1249274	NM_016426	NM_016426	ENST00000491863
NR_033985	FLJ26245	117.05114	5.6547878	-3.424722	0.0483104	NR_033985	NR_033985	ENST00000562769
NM_182908	DHRS2	4214.4115	676.3797	-3.430344	0.1604921	NM_182908	NM_182908	ENST00000556701
NM_001790	CDC25C	502.4272	39.64524	-3.442524	0.0789074	NM_001790	NM_001790	ENST00000323760
NM_138554	TLR4	1240.6766	152.71375	-3.446078	0.1230891	NM_138554	NM_138554	
NM_002546	TNFRSF11B	340.37205	16.046431	-3.452644	0.0471438	NM_002546	NM_002546	ENST00000521597
ENST00000399148		8893.296	1402.7075	-3.462949	0.1577264			ENST00000399148
NM_004838	HOMER3	52333.26	15246.195	-3.47197	0.291329	NM_004838	NM_004838	ENST00000539827
NM_001145722	HOMER3	72085.225	20947.195	-3.479177	0.2905893	NM_001145722	NM_001145722	ENST00000539827
NM_006074	TRIM22	1121.5078	86.08906	-3.480031	0.0767619	NM_006074	NM_006074	ENST00000379965
NM_004811	LPXN	920.9082	70.65177	-3.480779	0.0767197	NM_004811	NM_004811	ENST00000530561
NM_173843	IL1RN	109.17973	5.011873	-3.483065	0.0459048	NM_173843	NM_173843	ENST00000259206
NM_001166346	MDFIC	495.2065	37.92433	-3.483208	0.0765829	NM_001166346	NM_001166346	
NM_002422	MMP3	1094.1748	131.2442	-3.488626	0.1199481	NM_002422	NM_002422	ENST00000299855
NM_020299	AKR1B10	1225.5331	93.109845	-3.49405	0.075975	NM_020299	NM_020299	ENST00000359579
NM_018181	ZNF532	151.8941	6.8717685	-3.499715	0.0452405	NM_018181	NM_018181	ENST00000591083
NM_006072	CCL26	1519.0855	179.49485	-3.513352	0.1181598	NM_006072	NM_006072	ENST00000394905
NM_001276	CHI3L1	282.4391	12.61676	-3.514193	0.0446707	NM_001276	NM_001276	ENST00000404436
		157.3524	7.014361	-3.516582	0.0445774			
NM_031299	CDCA3	1990.927	233.4255	-3.52615	0.1172446	NM_031299	NM_031299	ENST00000536241
NM_005733	KIF20A	1299.9582	152.29237	-3.527455	0.1171517	NM_005733	NM_005733	ENST00000502338

NM_080668	CDCA5	4838.304	735.66815	-3.531687	0.1520508	NM_080668	NM_080668	ENST00000275517
NM_005429	VEGFC	25434.095	4761.421	-3.531696	0.1872062	NM_005429	NM_005429	ENST00000618562
NM_001211	BUB1B	1790.9955	208.70995	-3.536173	0.1165329	NM_001211	NM_001211	ENST00000412359
NM_133374	ZNF618	744.06795	54.29075	-3.549054	0.0729648	NM_133374	NM_133374	ENST00000615615
ENST00000435431	CKAP2L	1686.247	194.6655	-3.55164	0.1154431		AK097948	ENST00000435431
ENST00000399411		3008.3415	346.29965	-3.55635	0.1151131			ENST00000399411
NM_182687	PKMYT1	4200.873	629.6765	-3.558512	0.1498918	NM_182687	NM_182687	ENST00000574680
NM_001142620	STRA6	543.3325	39.27912	-3.561638	0.072293	NM_001142620	NM_001142620	ENST00000432245
NM_145252	ZG16B	515.21915	37.164225	-3.564655	0.0721328	NM_145252	NM_145252	ENST00000382280
NM_198433	AURKA	6010.503	894.29275	-3.572372	0.1487883	NM_198433	NM_198433	ENST00000347343
NM_032587	CARD6	2625.059	298.51105	-3.576452	0.1137159	NM_032587	NM_032587	ENST00000254691
NM_005127	CLEC2B	803.5596	57.215985	-3.582305	0.0712032	NM_005127	NM_005127	
NM_032997	ZWINT	1419.2385	160.46925	-3.58587	0.1130671	NM_032997	NM_032997	ENST00000318387
NM_138408	GTF3C6	12552.766	1852.1635	-3.588045	0.1475502	NM_138408	NM_138408	ENST00000329970
NM_153478	CSAG1	169.5561	7.078762	-3.591464	0.0417488	NM_153478	NM_153478	ENST00000452779
NM_018181	ZNF532	544.46805	38.397325	-3.595371	0.0705226	NM_018181	NM_018181	ENST00000336078
NM_001080538	AKR1B15	671.6986	47.15011	-3.6017	0.0701953	NM_001080538	NM_001080538	ENST00000423958
NM_001032281	TFPI	1207.9927	84.207385	-3.611168	0.0697085	NM_001032281	NM_001032281	ENST00000339091
NR_110429	LINC01540	615.7013	42.911005	-3.611442	0.0696945	NR_110429	NR_110429	ENST00000578664
NM_001290268	FAM65C	493.04475	33.95138	-3.627819	0.0688606	NM_001290268	NM_001290268	ENST00000045083
NR_045116	C5orf56	1257.293	138.5157	-3.628599	0.1101698	NR_045116	NR_045116	ENST00000378953
NM_001032731	OAS2	734.01554	50.481895	-3.629512	0.068775	NM_001032731	NM_001032731	ENST00000449768
NM_006461	SPAG5	5045.3405	725.3654	-3.636735	0.1437694	NM_006461	NM_006461	ENST00000580676
ENST00000417252		113.60231	4.5119195	-3.648458	0.0397168			ENST00000417252
NM_002875	RAD51	1219.3692	131.93225	-3.65834	0.1081971	NM_002875	NM_002875	ENST00000525066
ENST00000449439		167.454	6.58307	-3.660139	0.0393127			ENST00000449439
NM_017414	USP18	300.8634	11.810225	-3.661833	0.0392544	NM_017414	NM_017414	ENST00000215794
NM_001017535	VDR	1618.3745	174.03175	-3.668445	0.1075349	NM_001017535	NM_001017535	ENST00000229022
NM_012112	TPX2	6600.704	928.3987	-3.677861	0.1406515	NM_012112	NM_012112	ENST00000340513
NM_003500	ACOX2	1639.4197	175.08771	-3.679755	0.1067986	NM_003500	NM_003500	ENST00000460921
NM_198956	SP8	108.11597	4.1592255	-3.684889	0.03847	NM_198956	NM_198956	ENST00000617581
THC2626681		428.50155	28.24612	-3.687231	0.0659184			
NM_001080396	FAM155A	563.0431	37.05006	-3.689609	0.0658032	NM_001080396	NM_001080396	ENST00000375915
NM_014875	KIF14	1065.9058	113.04146	-3.691302	0.106052	NM_014875	NM_014875	ENST00000367350
ENST00000399411		1777.4395	188.1653	-3.694236	0.1058631			ENST00000399411
NM_006845	KIF2C	7378.968	1026.6563	-3.698224	0.1391328	NM_006845	NM_006845	ENST00000372217
NM_003483	HMGA2	10678.192	1484.9705	-3.699128	0.1390657	NM_003483	NM_003483	ENST00000403681
NM_006080	SEMA3A	339.5027	22.1655	-3.700301	0.0652881	NM_006080	NM_006080	ENST00000265362
NR_026934	LOC152225	428.2196	27.861355	-3.704996	0.0650632	NR_026934	NR_026934	ENST00000465215
NM_003873	NRP1	3477.741	365.6135	-3.705681	0.1051296	NM_003873	NM_003873	ENST00000374867
NM_205841	SPINK6	109.94339	4.1460855	-3.707649	0.0377111	NM_205841	NM_205841	ENST00000621437
NM_005322	HIST1H1B	1453.706	152.0462	-3.714118	0.1045921	NM_005322	NM_005322	ENST00000331442
NM_001072	UGT1A6	1395.7605	144.2997	-3.733237	0.1033843	NM_001072	NM_001072	ENST00000373409
NM_212557	AMTN	338.0869	12.46423	-3.733509	0.0368669	NM_212557	NM_212557	ENST00000339336
NM_001012507	CENPW	4024.727	547.9956	-3.738774	0.1361572	NM_001012507	NM_001012507	ENST00000368325
NM_014736	KIAA0101	9018.5729	1197.2697	-3.786224	0.132756	NM_014736	NM_014736	ENST00000300035
NM_031896	CACNG7	353.93005	12.41076	-3.79073	0.0350656	NM_031896	NM_031896	ENST00000391767
NM_003733	OASL	4237.3295	421.2327	-3.797763	0.0994099	NM_003733	NM_003733	ENST00000257570
NM_003820	TNFRSF14	1129.1765	67.71354	-3.815966	0.0599672	NM_003820	NM_003820	ENST00000466750
NM_017556	FBLIM1	3790.9967	494.4852	-3.819282	0.1304367	NM_017556	NM_017556	ENST00000375766
NM_015463	CNRIP1	373.73445	22.28839	-3.823478	0.059637	NM_015463	NM_015463	ENST00000481714
NM_016582	SLC15A3	1090.0223	64.69717	-3.82995	0.059354	NM_016582	NM_016582	ENST00000543406
NR_120307	LOC102724084	140.0989	4.6911165	-3.843436	0.0334843	NR_120307	NR_120307	
NR_038845	LYPLAL1-AS1	197.48525	6.5895705	-3.847432	0.0333674	NR_038845	NR_038845	
NM_030928	CDT1	1163.7542	111.62544	-3.856616	0.0959184	NM_030928	NM_030928	ENST00000301019
NM_001353	AKR1C1	7697.4925	982.4081	-3.860128	0.127627	NM_001353	NM_001353	ENST00000460124
NM_014271	IL1RAPL1	1304.5655	123.10457	-3.883502	0.0943644	NM_014271	NM_014271	ENST00000378993
NM_006739	MCM5	2203.009	207.0878	-3.889832	0.0940022	NM_006739	NM_006739	ENST00000216122
ENST00000504369		225.75985	7.23337	-3.893797	0.0320401			ENST00000504369
NM_003485	GPR68	1421.0355	80.04363	-3.901152	0.0563277	NM_003485	NM_003485	ENST00000535815
NR_125792	LINC01291	606.32261	33.713975	-3.918744	0.055604	NR_125792	NR_125792	ENST00000435984
NM_021158	TRIB3	24700.728	3843.2565	-3.919307	0.1555928	NM_021158	NM_021158	ENST00000217233
NM_002030	FPR3	123.98664	3.811885	-3.940952	0.0307443	NM_002030	NM_002030	ENST00000339223
NM_032117	MND1	1352.632	121.82032	-3.960321	0.0900617	NM_032117	NM_032117	ENST00000622785
NM_152754	SEMA3D	228.0606	6.8819375	-3.962268	0.0301759	NM_152754	NM_152754	ENST00000284136
NM_145697	NUF2	1217.26	109.09216	-3.968394	0.0896211	NM_145697	NM_145697	ENST00000271452
NM_001199042	STRA6	12024.744	1447.983	-3.969203	0.120417	NM_001199042	NM_001199042	ENST00000574439
NM_032997	ZWINT	4209.7321	506.58005	-3.970473	0.1203355	NM_032997	NM_032997	ENST00000489649
NM_025268	TMEM121	537.94915	28.77465	-3.971492	0.0534895	NM_025268	NM_025268	ENST00000392519
NM_153685	PIANP	127.1688	3.731712	-3.994179	0.0293446	NM_153685	NM_153685	ENST00000534837
NM_018410	HJURP	6069.4225	712.5754	-4.01673	0.1174042	NM_018410	NM_018410	ENST00000433484
NM_181801	UBE2C	14926.835	2215.4675	-4.018184	0.1484218	NM_181801	NM_181801	ENST00000617055
NM_001071	TYMS	14510.135	2150.7475	-4.020981	0.1482238	NM_001071	NM_001071	ENST00000581920
NM_000759	CSF3	1875.055	162.7187	-4.021406	0.0867808	NM_000759	NM_000759	ENST00000394148
NM_017523	XAF1	209.8729	5.9805565	-4.027693	0.0284961	NM_017523	NM_017523	ENST00000576724
NM_003328	TXK	170.61815	4.8597225	-4.028217	0.028483	NM_003328	NM_003328	ENST00000514937
NM_013282	UHRF1	2036.3135	174.0381	-4.046511	0.0854672	NM_013282	NM_013282	ENST00000624301
NM_003981	PRC1	5978.7435	686.5188	-4.058369	0.1148266	NM_003981	NM_003981	ENST00000361188
NM_003247	THBS2	129.6228	3.556067	-4.071082	0.027434	NM_003247	NM_003247	ENST00000488355
NM_001167915	VEPH1	545.1668	27.046725	-4.073883	0.0496118	NM_001167915	NM_001167915	ENST00000468233
NM_013358	PADI1	131.10985	3.5877545	-4.073978	0.0273645	NM_013358	NM_013358	ENST00000375471
ENST00000423781		737.31405	36.54034	-4.07534	0.0495587			ENST00000423781
NM_001005464	HIST2H3A	6030.856	686.23905	-4.075411	0.113788	NM_001005464	NM_001005464	ENST00000369158
NM_018492	PBK	4011.903	335.59735	-4.081879	0.0836504	NM_018492	NM_018492	ENST00000301905
NM_004666	VNN1	534.9779	26.079295	-4.097771	0.0487484	NM_004666	NM_004666	ENST00000367928
NM_032578	MYPN	398.49451	10.637151	-4.102342	0.0266933	NM_032578	NM_032578	ENST00000354393
ENST00000561588		1353.924	65.583615	-4.106414	0.0484397	XR_252919	XR_252919	ENST00000561588
NM_003542	HIST1H4C	83636.735	19400.56	-4.118015	0.2319622	NM_003542	NM_003542	ENST00000377803
NM_006737	KIR3DL2	139.15795	3.636307	-4.126672	0.0261308	NM_006737	NM_006737	ENST00000615373
NM_000710	BDKRB1	9057.511	1000.5921	-4.130903	0.110471	NM_000710	NM_000710	ENST00000611804
ENST00000438543		138.55365	3.5583775	-4.146447	0.0256823		AK126057	ENST00000438543
NM_004701	CCNB2	10409.477	1138.919	-4.148974	0.1094117	NM_004701	NM_004701	ENST00000621385

NM_003265	TLR3	296.1764	7.5729925	-4.151489	0.0255692	NM_003265	NM_003265	ENST00000512264
NM_020299	AKR1B10	1429.783	114.11089	-4.15924	0.0798099	NM_020299	NM_020299	ENST00000359579
XR_244444	LOC101927499	176.17225	4.439565	-4.168096	0.0252001	XR_244444	XR_244444	
NM_001291317	MILR1	150.65742	3.785183	-4.171532	0.0251244	NM_001291317	NM_001291317	ENST00000612535
NM_202002	FOXM1	2128.649	168.09145	-4.176732	0.0789663	NM_202002	NM_202002	ENST00000359843
NM_017527	LY6K	56890.745	12879.15	-4.187026	0.2263839	NM_017527	NM_017527	ENST00000292430
NM_020904	PLEKHA4	1246.5304	97.400765	-4.194099	0.0781375	NM_020904	NM_020904	ENST00000263265
ENST00000453951	LINC01293	488.18995	22.11652	-4.197494	0.0453031	XR_245025	XR_245025	ENST00000453951
NM_016293	BIN2	162.1461	3.8853265	-4.225648	0.0239619	NM_016293	NM_016293	ENST00000267012
NM_020675	SPC25	2760.819	211.58875	-4.225955	0.0766398	NM_020675	NM_020675	ENST00000611144
NM_001165252	KRTAP2-3	332.71845	7.964925	-4.226742	0.0239389	NM_001165252	NM_001165252	ENST00000391418
NM_002534	OAS1	353.47225	8.3453715	-4.242561	0.0236097	NM_002534	NM_002534	ENST00000549820
NM_017779	DEPDC1	2476.0345	187.40562	-4.24653	0.0756878	NM_017779	NM_017779	ENST00000489862
NM_033292	CASP1	1521.4805	65.55483	-4.265757	0.0430862	NM_033292	NM_033292	ENST00000534497
NM_002462	MX1	1137.4876	48.5422	-4.278806	0.0426749	NM_002462	NM_002462	ENST00000455164
NM_001159	AOX1	1345.2675	57.05988	-4.28711	0.0424153	NM_001159	NM_001159	ENST00000485106
ENST00000586049		167.9255	3.8020955	-4.290387	0.0226416			ENST00000586049
NM_203380	ACSL5	1596.4233	117.60813	-4.291014	0.0736698	NM_203380	NM_203380	ENST00000354655
NM_001012271	BIRC5	24987.46	3240.6134	-4.300905	0.1296896	NM_001012271	NM_001012271	ENST00000301633
NM_032229	SLITRK6	404.32625	9.043225	-4.304367	0.0223662	NM_032229	NM_032229	ENST00000400286
NM_017523	XAF1	430.8811	18.02545	-4.305886	0.0418339	NM_017523	NM_017523	ENST00000361842
NM_005613	RGS4	163.5782	3.603227	-4.321792	0.0220276	NM_005613	NM_005613	ENST00000527393
NM_000800	FGF1	158.09845	3.4482445	-4.33309	0.0218107	NM_000800	NM_000800	ENST00000441680
NM_007105	SLC22A18AS	1748.67	124.94099	-4.341393	0.0714491	NM_007105	NM_007105	ENST00000613450
NM_181755	HSD11B1	4508.578	320.30055	-4.350789	0.0710425	NM_181755	NM_181755	ENST00000367028
ENST00000594169		151.326	3.1944865	-4.370393	0.02111	XR_109656	XR_109656	ENST00000594169
NM_001032998	KYNU	2805.1545	196.3944	-4.37484	0.070012	NM_001032998	NM_001032998	ENST00000460143
NM_018101	CDCA8	8304.0195	803.8125	-4.378732	0.096798	NM_018101	NM_018101	ENST00000327331
NM_007115	TNFAIP6	1494.0315	103.6695	-4.38955	0.0693891	NM_007115	NM_007115	ENST00000460812
lnc-SLC15A4-12:2	lnc-SLC15A4-12	187.06065	3.790941	-4.417007	0.0202658			
NM_017459	MFAP2	251.2075	5.076367	-4.420279	0.0202079	NM_017459	NM_017459	ENST00000490075
NM_004460	FAP	5998.765	565.2857	-4.429093	0.0942337	NM_004460	NM_004460	ENST00000188790
NM_000361	THBD	5927.388	554.2692	-4.443556	0.0935099	NM_000361	NM_000361	ENST00000377103
NM_016228	AADAT	201.62455	3.917403	-4.465163	0.0194292	NM_016228	NM_016228	ENST00000353187
NM_004585	RARRES3	6653.0535	614.1828	-4.467659	0.0923159	NM_004585	NM_004585	ENST00000354445
NM_001511	CXCL1	137382.7	28113.38	-4.47337	0.2046355	NM_001511	NM_001511	ENST00000395761
NM_005211	CSF1R	528.54561	19.430918	-4.481692	0.036763	NM_005211	NM_005211	ENST00000286301
NM_024621	VEPH1	614.55465	11.63444	-4.494805	0.0189315	NM_024621	NM_024621	ENST00000392832
NM_006101	NDC80	7315.8385	664.4614	-4.498198	0.0908251	NM_006101	NM_006101	ENST00000261597
NM_001615	ACTG2	3235.664	209.60845	-4.50267	0.0647807	NM_001615	NM_001615	ENST00000438902
NM_005130	FGFBP1	524.1048	9.8518365	-4.502921	0.0187975	NM_005130	NM_005130	ENST00000382333
NM_001159	AOX1	602.51115	21.581455	-4.517077	0.0358192	NM_001159	NM_001159	ENST00000465297
NM_006417	IFI44	3929.273	249.67215	-4.534459	0.0635416	NM_006417	NM_006417	ENST00000485662
NM_000759	CSF3	17331.81	1524.856	-4.557889	0.0879802	NM_000759	NM_000759	ENST00000579852
NM_000600	IL6	6816.938	594.4838	-4.574449	0.0872069	NM_000600	NM_000600	ENST00000485300
NM_152899	IL4I1	1231.187	41.600805	-4.596455	0.0337892	NM_152899	NM_152899	ENST00000595948
ENST00000423781		610.4444	10.479658	-4.606543	0.0171673			ENST00000423781
NM_004833	AIM2	1917.578	115.99957	-4.615396	0.0604928	NM_004833	NM_004833	ENST00000612470
ENST00000481132	TFPI	3162.259	185.4236	-4.666699	0.0586364		AB209866	ENST00000481132
ENST00000449712	LINC00704	224.21785	3.6288335	-4.673885	0.0161844			ENST00000449712
NM_006681	NMU	235.5403	3.795435	-4.678884	0.0161137	NM_006681	NM_006681	ENST00000264218
NM_080657	RSAD2	462.4952	7.2918335	-4.703783	0.0157663	NM_080657	NM_080657	ENST00000382040
NM_023915	GPR87	380.91341	5.6363567	-4.776262	0.014797	NM_023915	NM_023915	ENST00000260843
ENST00000515153		233.93325	3.405696	-4.794826	0.0145584			ENST00000515153
NM_001122679	TENM2	1656.5895	87.835945	-4.832366	0.0530222	NM_001122679	NM_001122679	ENST00000518659
NM_001017402	LAMB3	21054.245	2106.1235	-4.844998	0.1000332	NM_001017402	NM_001017402	ENST00000367030
NM_001175	ARHGDIB	6396.8085	480.67435	-4.853727	0.0751428	NM_001175	NM_001175	ENST00000228945
NM_001099294	KIAA1644	273.8665	3.6184615	-4.90563	0.0132125	NM_001099294	NM_001099294	
NM_001465	FYB	873.27095	23.11241	-4.928786	0.0264665	NM_001465	NM_001465	ENST00000351578
NM_002201	ISG20	2181.1185	107.54383	-4.951949	0.0493067	NM_002201	NM_002201	ENST00000558942
NM_024943	TMEM156	281.58825	3.5269265	-4.966658	0.0125251	NM_024943	NM_024943	ENST00000381938
NR_046173	LOC254896	1604.6175	40.576895	-4.990781	0.0252876	NR_046173	NR_046173	
NM_004864	GDF15	2507.0145	118.50173	-5.021454	0.0472681	NM_004864	NM_004864	ENST00000252809
NM_001122679	TENM2	6373.647	288.84525	-5.090778	0.0453187	NM_001122679	NM_001122679	ENST00000545108
NM_014903	NAV3	588.99975	13.38656	-5.135996	0.0227276	NM_014903	NM_014903	ENST00000397909
NM_033439	IL33	21188.765	1812.888	-5.172526	0.0855589	NM_033439	NM_033439	ENST00000456383
NM_006287	TFPI	4579.8675	192.9228	-5.211102	0.0421241	NM_006287	NM_006287	ENST00000233156
NM_173553	TRIML2	3981.989	161.5172	-5.273305	0.0405619	NM_173553	NM_173553	ENST00000512729
NM_031957	KRTAP1-5	1509.4197	30.954195	-5.275857	0.0205073	NM_031957	NM_031957	ENST00000361883
NM_021013	KRT34	1897.021	74.599265	-5.324307	0.0393244	NM_021013	NM_021013	ENST00000394001
NM_007193	ANXA10	1484.0135	27.533885	-5.41207	0.0185537	NM_007193	NM_007193	ENST00000617524
NR_024475	LINC00704	562.40595	4.7592705	-5.414546	0.0084623	NR_024475		ENST00000430998
NM_022147	RTP4	933.9438	16.84139	-5.450831	0.0180326	NM_022147	NM_022147	ENST00000259030
NM_001098845	ANXA8L1	6540.511	222.998	-5.559193	0.0340949	NM_001098845	NM_001098845	ENST00000584982
NR_024475	LINC00704	470.1607	3.4756695	-5.56893	0.0073925	NR_024475	NR_024475	ENST00000430998
NM_000576	IL1B	27737.877	1930.2872	-5.605426	0.0695903	NM_000576	NM_000576	ENST00000263341
NR_033931	LINC01085	599.3517	4.2441745	-5.618063	0.0070813	NR_033931	NR_033931	ENST00000506739
NR_033931	LINC01085	2376.353	34.218185	-5.756939	0.0143995	NR_033931	NR_033931	ENST00000502759
NM_006820	IFI44L	754.00804	10.814731	-5.762284	0.014343	NM_006820	NM_006820	ENST00000476521
NM_004091	E2F2	683.27575	4.0440525	-5.822925	0.0059186	NM_004091	NM_004091	ENST00000361729
NM_000758	CSF2	718.9476	4.1277975	-5.857642	0.0057414	NM_000758	NM_000758	ENST00000296871
NM_144569	SPOCD1	6757.9535	290.9544	-5.898388	0.0430536	NM_144569	NM_144569	ENST00000473361
NM_001042483	NUPR1	19551.775	698.83125	-6.247462	0.0357426	NM_001042483	NM_001042483	ENST00000324873
NM_000963	PTGS2	1232.544	4.8877127	-6.28035	0.0039655	NM_000963	NM_000963	ENST00000367468
NM_002185	IL7R	3498.4334	27.19387	-6.595738	0.0077732	NM_002185	NM_002185	ENST00000303115
NM_000640	IL13RA2	11077.981	301.4331	-6.759068	0.0272101	NM_000640	NM_000640	ENST00000243213
NM_003020	SCG5	6956.0265	36.705505	-8.630414	0.0052768	NM_003020	NM_003020	ENST00000475752
ENST00000425195	LOC102725370	27276.98	135.8275	-9.94447	0.0049796	XR_425587	XR_425587	ENST00000425195
